# Chain Extension of Piperazine in Ethanol: Synthesis of 2-(4-(2-(Phenylthio)ethyl)piperazinyl)acetonitriles and ACAT-1 Inhibitors

**DOI:** 10.3390/molecules29163723

**Published:** 2024-08-06

**Authors:** Ying Huang, Tingyu Zhu, Yinghua Li, Deguang Huang

**Affiliations:** 1 State Key Laboratory of Structural Chemistry, Fujian Institute of Research on the Structure of Matter, University of Chinese Academy of Sciences, Fuzhou 350002, China; huangying@fjirsm.ac.cn (Y.H.); zhutingyu@fjirsm.ac.cn (T.Z.); 2 College of Chemistry and Materials Science, Fujian Normal University, Fuzhou 350007, China; 3 Fujian College, University of Chinese Academy of Sciences, Fuzhou 350002, China

**Keywords:** green solvent, catalyst-free reaction, sulfur-containing ethyl piperazine, ACAT-1 inhibitors, medicinal chemistry

## Abstract

A base-induced synthesis of 2-(4-(2-(phenylthio)ethyl)piperazinyl) acetonitriles by reaction of disulfides, 1-(chloromethyl)-4-aza-1-azonia bicyclo[2.2.2]octane chloride and trimethylsilyl cyanide is reported. The scope of the method is demonstrated with 30 examples. The reaction mechanism research indicates that the three-component reaction would be a SN2 reaction. The products exhibit good activities towards advanced synthesis of aqueous soluble acyl-CoA: cholesterol *O*-acyltransferase-1 (ACAT-1) inhibitors. Our work is superior as it uses less-odor disulfides as carbon sources and EtOH as solvent in a water and dioxygen insensitive reaction system, followed by a simple purification process.

## 1. Introduction

**Figure 1:**
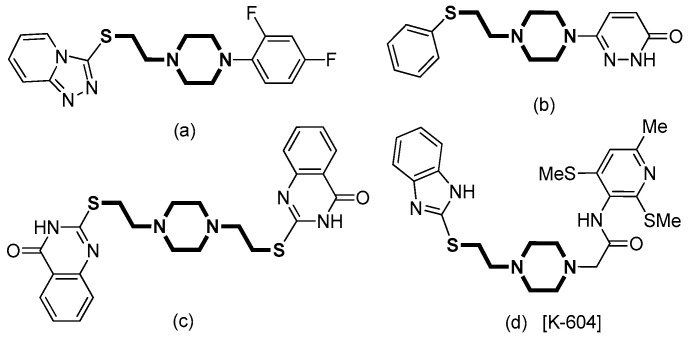
Representative bioactive sulfur-containing ethyl piperazine compounds (**a**) [23], (**b**) [6], (**c**) [1], and (**d**) [8,20].

The incorporation of piperazine into medicinal molecules is proved effective in the increase in aqueous solubility and therefore in the improvement of oral absorption and bioavailability. It is of high importance in medication safety for further development of clinical candidates. Sulfur-containing ethyl piperazine compounds and their derivatives (Figure 1) are important building blocks in pharmaceuticals. Their biochemical activities were discovered and explored in the treatment of cancer [1], glioblastoma [2,3], atherosclerosis [4], anxiety neurosis [5] and the reduction of blood pressure [6]. The 2-mercaptobenzimidazole bound ethyl piperazine is recognized as a targeting moiety for the structural accommodation of 2-(4-(2-((1*H*-Benzo[*d*]imidazol-2-yl)thio)-ethyl)piperazin-1-yl)-*N*-(6-methyl-2,4-bis(methylthio)-pyridin-3-yl)acetamide hydrochloride [K-604], an acyl-CoA: cholesterol *O*-acyltransferase-1 (ACAT-1) inhibitor (Figure 1d). The enzymes catalyze cholesterol esterification with acylcoenzyme A [7]. They have received attention as promising drug targets for the treatment of diseases such as hyperlipidemia [8], neurodegenerative disease [9,10,11,12,13,14], cancer [15,16], leukemia [17], and bleomycin-induced lung injury [18].

**Scheme 1:**
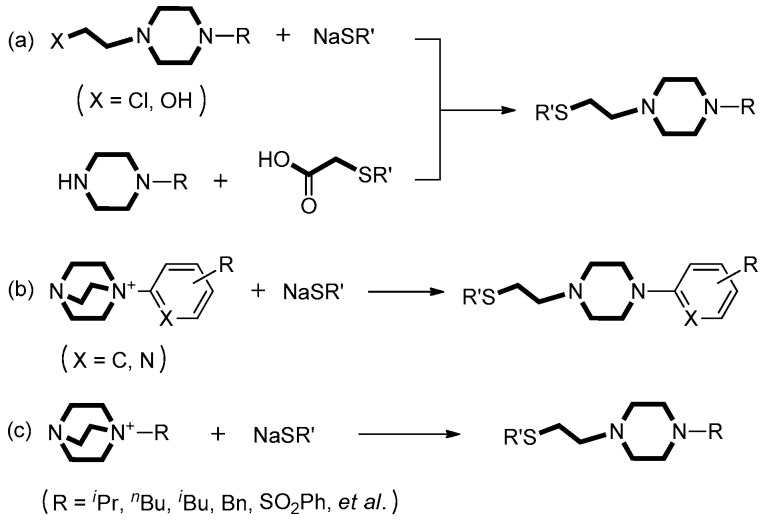
Different strategies (**a**) [8,19,20,21,22], (**b**) [23,24,25], and (**c**) [26,27,28,29] to synthesize sulfur-containing ethyl piperazine compounds.

Sulfur-containing ethyl piperazine compounds were originally obtained by introducing thiol groups on the side chain of the 4-substituted 1-(2-chloroethyl)piperazine or 1-(2-hydroxyethyl) piperazine via nucleophilic substitution reactions [8,19,20,21,22] (Scheme 1). A ring-opening method was developed using cyclic tertiary amines as the ethyl piperazine source enabled by the incorporation of thiolates through facile C-N bond cleavage. Reaction of aromatic halogenated compounds with triethylenediamine (DABCO) in the presence of Na_2_S as the sulfur source at 120 °C afforded the products 1-(2-(pyridin-2-yl)ethyl)-4-(pyridin-2-yl)piperazines in considerable yields [23] (Scheme 1b). Similar products were obtained by reaction of either *o*-silyl aryl triflates or pyridine-*N*-oxides with thiolates in the presence of CsF or trifluoroacetic anhydride as the activating agent, respectively [24,25]. The method was extended using 1-alkyl group bound 4-aza-1-azoniabicyclo[2.2.2]octane as the ethyl piperazine source under alkaline conditions [26,27,28,29]. Although significant advances have been made, the synthesis of sulfur-containing ethyl piperazine compounds is of interest to scientists. Up to now, almost all the works concerning the building of the sulfur-containing ethyl piperazine skeleton have said that the C-N bond cleavage of the cyclic tertiary amines was achieved in two steps: quaternization first, ring-opening second. Synthesis of the sulfur-containing ethyl piperazine compound by a SN2 reaction was rarely reported.

**Scheme 2:**
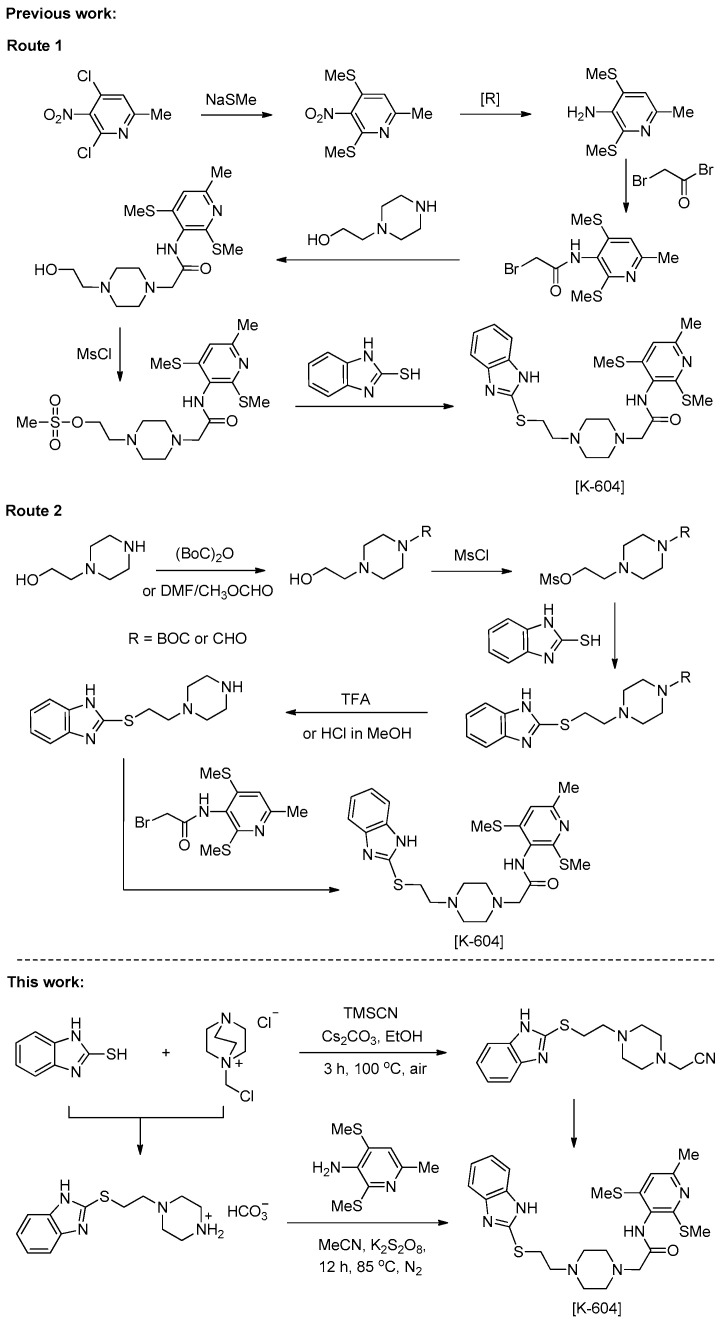
Different strategies (route 1 [8]; route 2 [20,30,31,32]) to synthesize the ACAT-1 inhibitor [K-604].

To date, there are mainly two routes to prepare the sulfur-containing ethyl piperazine compound [K-604] and its derivatives [8,20,30,31,32] (Scheme 2). The non-tertiary amine 1-(2-hydroxyethyl)piperazine was employed as the starting material in the routes, and the target product was prepared by multi-step reactions. Interestingly, the tertiary amine 4-aza-1-azoniabicyclo[2.2.2]octane has not been used for the synthesis of ACAT-1 inhibitors. Therefore, we report here a simple and eco-friendly method for the synthesis of various 2-(4-(2-(phenylthio)ethyl)piperazinyl)acetonitriles (**2**) by a three-component SN2 disubstitution reaction, using 1-(chloromethyl)-4-aza-1-azonia bicyclo[2.2.2]octane chloride (CAABC) as the ethyl piperazine source, disulfide as the thiol source, trimethylsilyl cyanide (TMSCN) as the cyanide source, and EtOH as the solvent. The products were easily obtained by a simple purification process. They can be applied to the preparation of [K-604] and its derivatives in two steps.

## 2. Results and Discussion

Reaction of diphenyl disulfide (**1a**, 0.1 mmol), CAABC (0.2 mmol), TMSCN (0.22 mmol) and Cs_2_CO_3_ (0.6 mmol) under air atmosphere in EtOH (1 mL) for 3 h at 100 °C provided the product 2-(4-(2-(phenylthio)ethyl)piperazinyl)acetonitrile (**2a**) in 90% yield (based on diphenyl disulfide, Table 1, entry 1). Other alkali salts, such as K_2_CO_3_, Na_2_CO_3_, KOH, and *^t^*BuOK, afforded the product in lower yields (entries 2–5). The addition of a trace of water had little effect on production, but a greater amount of water (7:3) would lead to the generation of **2a** in a lower yield (entry 6). Only a trace of product could be found when the reaction was performed in a clear aqueous solution (entry 7). The use of MeOH as the solvent provided **2a** in 75% yield (entry 8). With other polar solvents, such as DMF and DMSO, no corresponding product could be obtained (entries 9 and 10). Higher temperature would not help to improve the yield, while a lower temperature would decrease the reaction (entries 11 and 12). A period of 3 h would be enough for the completeness of the reaction. A longer or shorter time is of no advantage to the reaction (entries 13 and 14).

**Figure 2:**
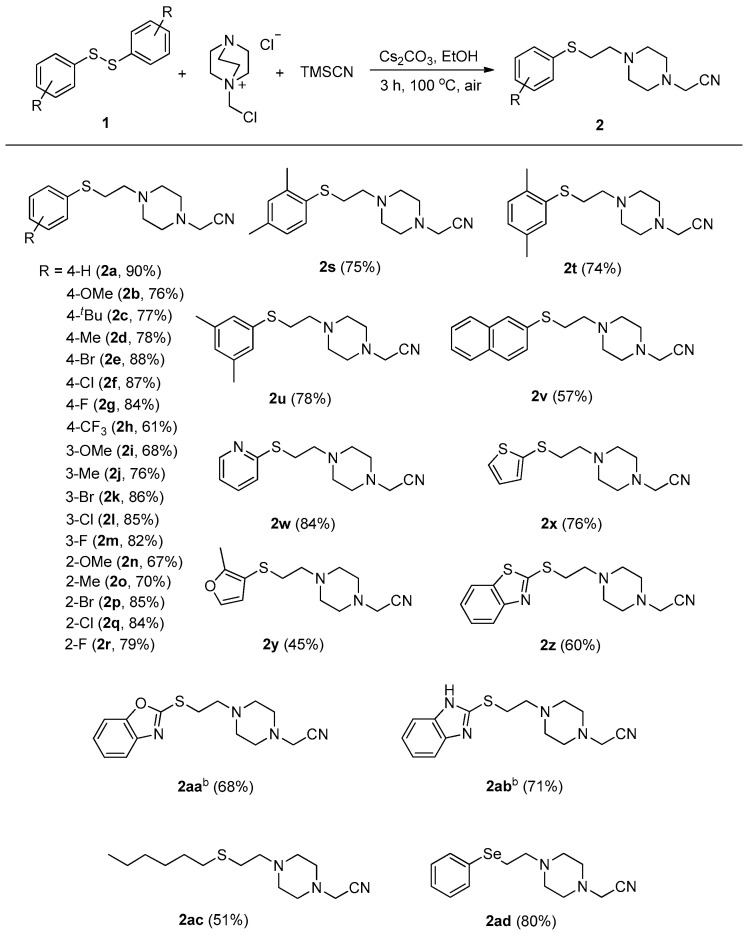
Scope of disulfides with respect to compounds **2** ^a,b^; ^a^ Reaction conditions: **1** (0.1 mmol), CAABC (0.2 mmol), TMSCN (0.22 mmol), Cs_2_CO_3_ (0.6 mmol), EtOH (1 mL), air, 100 °C, 3 h, isolated yields. ^b^ Reactions were conducted using 2-mercaptobenzoxazole (0.2 mmol) and 2-mercapto benzimidazole (0.2 mmol) as substrates.

At the optimized conditions, the scope of the substrates was investigated for the production of **2** (Figure 2). Thirty compounds were prepared in terms of the electronic effect and steric effect of the functional groups on the substrates. It was found that both the electron-donating groups and the electron-withdrawing groups on the benzene ring of diphenyl sulfides would lead to the reduction of yields. By contrast, the electron-donating groups (**2b**–**2d**) might have a larger effect than the electron-withdrawing groups (**2e**–**2g**), except for the strong electron-withdrawing groups CF_3_ by which the yield decreased dramatically, down to 61%. This inference was consistent with the experimental results obtained from the comparison of compounds **2i** and **2j** with **2k**–**2m**, and **2n** and **2o** with **2p**–**2r**. The influence of steric hindrance on the reaction was studied by the employment of methoxyl-(**2b**, **2i** and **2n**), methyl-(**2d**, **2j** and **2o**), Br-(**2e**, **2k** and **2p**), Cl-(**2f**, **2l** and **2q**), and F-(**2g**, **2m** and **2r**) groups at the *para*, *meta*, and *ortho* positions of the benzene rings. The results showed that the steric effect had little impact on the production of the target products. It was in accordance with the reaction of the disubstituted diphenyl disulfide under the same conditions (**2s**–**2u**, 74–78%). Our reaction exhibited good compatibility with other cyclic thiol sources, such as 2-naphthalenethiol (**2v**), 2-mercaptopyridine (**2w**), thiophenethiol (**2x**), 2-methyl-3-furanthiol (**2y**), 2-benzothiazolethiol (**2z**), 2-benzoxazolethiol (**2aa**), and 2-mercaptobenzimidazole (**2ab**). The corresponding products were obtained in yields of 57–84%. In addition, the reaction of *n*-hexyl disulfide (**2ac**) or diphenyl diselenide (**2ad**) under the standard conditions also produced the desired products in yields of 51% and 80%, respectively. These results implicate a relatively broad range of substrates in our reaction. In addition, the synthetic utility of reaction was checked by performing the experiments on the gram scale. The reaction of diphenyl disulfide (**1a**) and CAABC on a 1.5 g scale produced compound **2a** in 90% yield (Supporting Information, Section S2.4), which implies that the amount of the starting material did not directly influence the quality of reaction, and our reaction was suitable for the production of sulfur-containing ethyl piperazine compounds for further synthesis.

**Scheme 3:**
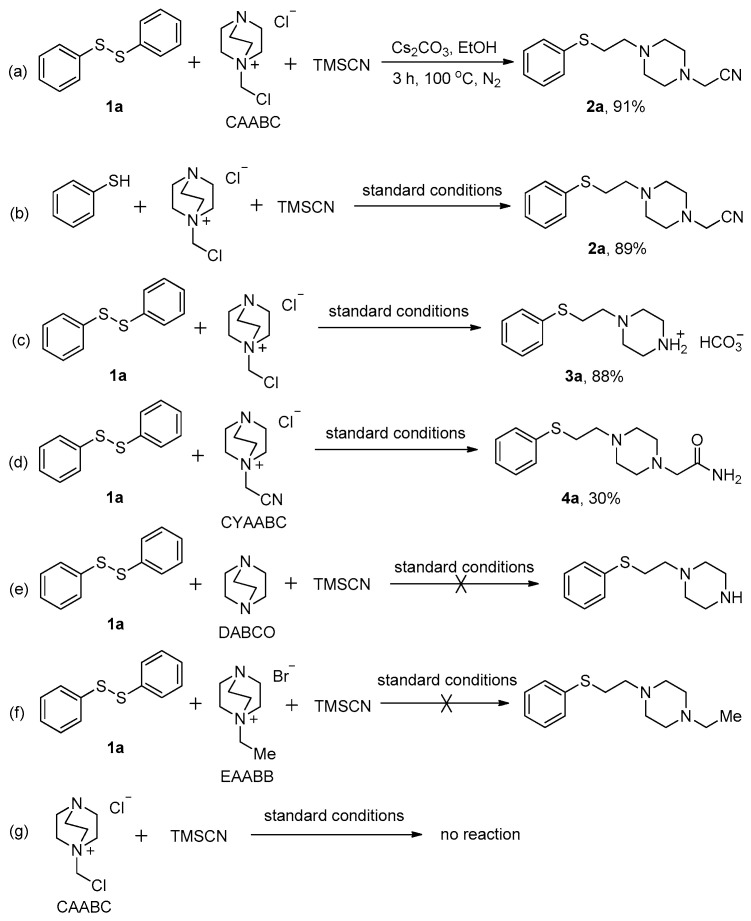
Control experiments: (**a**) Study the influence of O_2_ on the yield; (**b**) Examine the reaction using thiophene as the thiolate source; (**c**) Examine the reaction in the absence of TMSCN; (**d**) Examine the reaction by the replacement of CAABC with CYAABC; (**e**) Examine the reaction by the replacement of CAABC with DABCO; (**f**) Examine the reaction by the replacement of CAABC with EAABB; (**g**) Check the standard reaction without the presence of PhSSPh.

With the progress of research on compounds **2**, a number of experiments were conducted to study the reaction mechanism. The reaction of diphenyl disulfide (**1a**) with CAABC, TMSCN and Cs_2_CO_3_ under N_2_ atmosphere in EtOH afforded the product **2a** in 91% yield (Scheme 3a). The reaction of thiophenol with CAABC and TMSCN under the standard conditions also provided the product **2a** in similar yield (Scheme 3b). It meant that the reaction was independent of dioxygen and the thiophenol could be an intermediate of reaction. Compound **3a** was obtained when the reaction was repeated in the absence of TMSCN. The result was checked using TMSCF_3_ and Et_4_NCN as the nucleophiles. The reaction of **1a**, CAABC and TMSCF_3_ under the standard conditions afforded **3a** in a similar yield. With the replacement of TMSCF_3_ by Et_4_NCN, compound **2a** was obtained in 60% yield. It indicated that cyanide anion was the right nucleophile for the substitution of the chloride group, and the chloromethyl group might work as a leaving group without the presence of CN^−^. Meanwhile, the reaction of **1a** with 1-(cyanomethyl)-4-aza-1-azonia bicyclo[2.2.2]octane chloride (CYAABC) produced a piperazine amide compound **4a** rather than the desired compound **2a**. They seemed to be SN2 reactions with the attack of sulfide (obtained by the reduction of diphenyl disulfide) on the ethylene group of the DABCO ring on one side, and the attack of the hydroxyl ion (generated by the alkalization of H_2_O in EtOH) on the cyanomethyl groups of the other side. The speculation was supported by the reaction of **1a** and TMSCN with triethylenediamine (DABCO) or 1-ethyl-4-aza-1-azonia bicyclo[2.2.2]octane bromide (EAABB), from which no desired product could be observed (Scheme 3e,f). At last, there was no reaction between CAABC and TMSCN under the standard conditions.

**Scheme 4:**
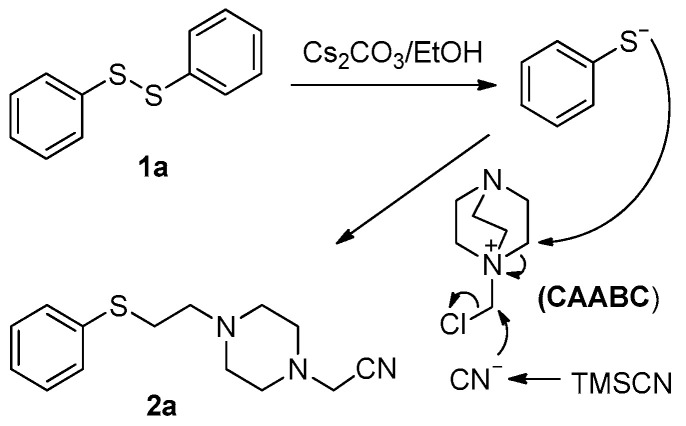
Proposed reaction mechanism.

Based on the above results, a possible reaction mechanism was proposed in Scheme 4. Diphenyl disulfide is reduced to thiophenolate in the presence of Cs_2_CO_3_ in EtOH when heated. The PhS^−^ anion attacks CAABC on the ethylene with the attack of CN^−^ on the chloromethyl group to yield the desired compound **2a**.

**Scheme 5:**
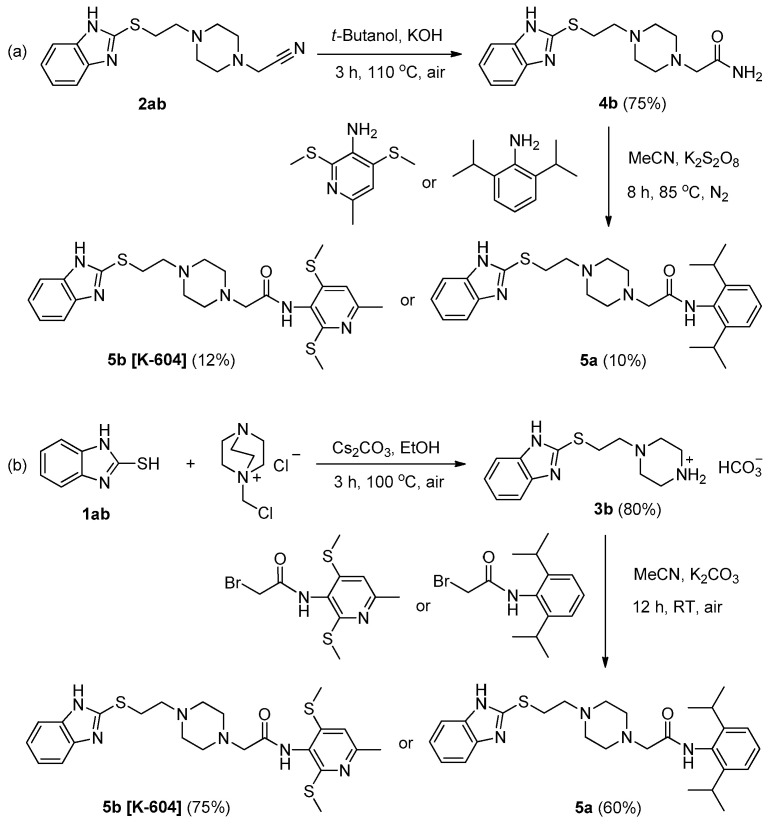
Exploration of the synthesis of ACAT-1 inhibitors **5a** and **5b**: (**a**) Our method 1 to prepare [K-604]; (**b**) Our method 2 to prepare [K-604].

**Figure 3:**
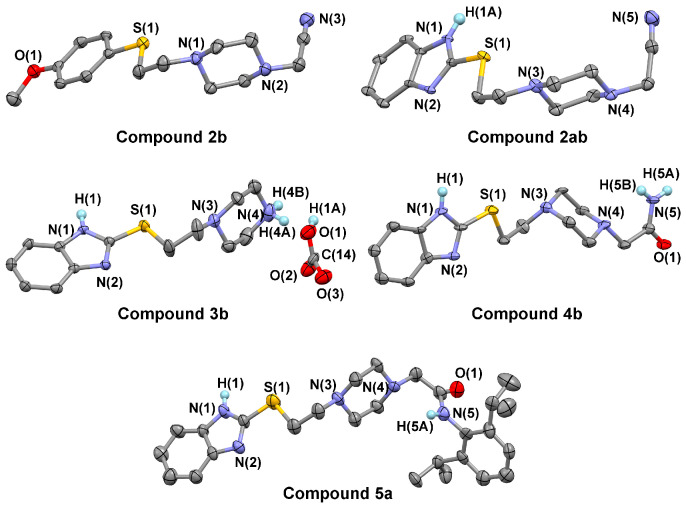
Crystal structures of compounds **2b** (CCDC 2328305), **2ab** (CCDC 2328309), **3b** (CCDC 2328313), **4b** (CCDC 2328311) and **5a** showing thermal ellipsoids of 50% probability surfaces. The structure of **5a** was solved by fast-data collection with the crystallographic data shown in Supplementary Materials.

Our reaction exhibited good tolerance towards the transformation of aromatic thiols and/or disulfides to ACAT-1 inhibitors. Two routes were discovered in this work, one by acylation of compounds **2**, the other by cutting the chloromethyl group off from the piperazine (Scheme 5). The stirring of compound **2ab** in the presence of KOH under air atmosphere in *^t^*BuOH for 3 h at 110 °C afforded compound **4b** in 75% yield. Reaction of **4b** with 2,6-diisopropylaniline and 6-methyl-2,4-bis(methylthio)pyridin-3-amine provided the desired products **5a** and **5b** in yields of 10 and 12%, respectively. The poor solubility of **4b** in MeCN restricted its application. Other polar solvents, such as MeOH, H_2_O and DMF, would cause the increase in by-products. Although the method had its disadvantage, it offered a chance to improve the rate of production. The reaction of 2-mercaptobenzimidazole (**1ab**) with CAABC in the absence of TMSCN under the standard conditions produced compound **3b** in 80% yield, which was further reacted with 2-bromo-*N*-(2,6-diisopropylphenyl)acetamide and 2-bromo-*N*-(6-methyl-2,4-bis(methylthio)pyridin-3-yl)acetamide to yield compounds **5a** and **5b** in yields of 60% and 75%. In comparison to what has been described in the literatures, our method is superior in offering fewer reaction steps with a similar yield in EtOH (first step). The structures of compounds were determined by X-ray crystallography and are shown in Figure 3 (**2b**, **2ab**, **3b**, **4b**, and **5a**) and Figure S1 (**2w**, **2z** and **2ab**), respectively.

## 3. Experimental Section

### 3.1. General Preparations

Chemicals: Unless otherwise stated, all commercial-grade chemicals were used without further purification. 6-Methyl-2,4-bis(methylthio)-pyridin-3-amine, 2-bromo-*N*-(2,6-diisopropylphenyl) acetamide and 2-bromo-*N*-(6-methyl-2,4-bis(methylthio)pyridin-3-yl)acetamide were prepared according to the reported methods (see Supporting Information).

### 3.2. Synthesis Procedures

#### 3.2.1. General Procedure for the Synthesis of Compounds **2**

Disulfides **1** (0.1 mmol), 1-(chloromethyl)-4-aza-1-azonia bicyclo[2.2.2]octane chloride (CAABC) (0.2 mmol), trimethylsilyl cyanide (0.22 mmol), Cs_2_CO_3_ (0.6 mmol) and EtOH (1 mL) were mixed in a 50 mL Teflon screw-cap sealed tube. The mixture was vigorously stirred under air atmosphere for 3 h at 100 °C (oil bath). After cooling to room temperature, the reaction mixture was filtered. The precipitate was washed with EtOH (2 mL). The organic layers were combined and flashed through a pad of silica gel (3 mL) in pipette eluted with petroleum ether/EtOH (10:1 to 5:1 *v*/*v*) (10 mL) to yield the products **2**. The yields and the characterization data of products are shown on pages S11–S17; ^1^H NMR spectra are presented on pages S31–S60 (SI).

2-(4-(2-(phenylthio)ethyl)piperazinyl)acetonitrile (**2a**): yield, 90% (47.1 mg); light yellow oil. ^1^H NMR (400 MHz, CDCl_3_) δ 7.32 (d, J = 7.3 Hz, 2H), 7.26 (t, *J* = 7.6 Hz, 2H), 7.15 (t, *J* = 7.2 Hz, 1H), 3.46 (s, 2H), 3.02 (t, *J* = 7.2 Hz, 2H), 2.65–2.47 (m, 10H). ^13^C NMR (101 MHz, CDCl_3_) δ 136.3, 129.0, 128.97, 126.0, 114.8, 57.4, 52.5, 51.7, 45.9, 30.8. HRMS (ESI) m/z [M + H]^+^ calcd for C_14_H_20_N_3_S, 262.1372; found, 262.1375.

2-(4-(2-((4-methoxyphenyl)thio)ethyl)piperazinyl)acetonitrile (**2b**): yield, 76% (44.3 mg); light yellow solid. M.p. 88–89 °C. ^1^H NMR (400 MHz, CDCl_3_) δ 7.32 (d, *J* = 8.7 Hz, 2H), 6.81 (d, *J* = 8.7 Hz, 2H), 3.76 (s, 3H), 3.46 (s, 2H), 2.90 (t, *J* = 7.6 Hz, 2H), 2.59–2.42 (m, 10H). ^13^C NMR (101 MHz, CDCl_3_) δ 159.0, 133.3, 126.1, 114.8, 114.6, 57.7, 55.4, 52.5, 51.7, 45.9, 32.8. HRMS (ESI) *m*/*z* [M + H]^+^ calcd for C_15_H_22_N_3_OS, 292.1484; found, 292.1487.

2-(4-(2-((4-(*tert*-butyl)phenyl)thio)ethyl)piperazinyl)acetonitrile (**2c**): yield, 77% (48.9 mg); light yellow oil. ^1^H NMR (400 MHz, CDCl_3_) δ 7.33–7.26 (m, 4H), 3.49 (s, 2H), 3.02 (t, *J* = 7.2 Hz, 2H), 2.66–2.49 (m, 10H), 1.30 (s, 9H). ^13^C NMR (101 MHz, CDCl_3_) δ 149.4, 132.6, 129.3, 126.0, 114.8, 57.5, 52.5, 51.7, 45.9, 34.5, 31.3, 31.2. HRMS (ESI) *m*/*z* [M + H]^+^ calcd for C_18_H_28_N_3_S, 318.2004; found, 318.2007.

2-(4-(2-(*p*-tolylthio)ethyl)piperazinyl)acetonitrile (**2d**): yield, 78% (43.0 mg); light yellow oil. ^1^H NMR (400 MHz, CDCl_3_) δ 7.23 (d, *J* = 8.1 Hz, 2H), 7.07 (d, *J* = 8.0 Hz, 2H), 3.46 (s, 2H), 2.97 (t, *J* = 7.6 Hz, 2H), 2.64–2.45 (m, 10H), 2.29 (s, 3H). ^13^C NMR (101 MHz, CDCl_3_) δ 136.3, 132.3, 130.0, 114.8, 57.5, 52.5, 51.7, 45.9, 31.4, 21.1. HRMS (ESI) *m*/*z* [M + H]^+^ calcd for C_15_H_22_N_3_S, 276.1534; found, 276.1538.

2-(4-(2-((4-bromophenyl)thio)ethyl)piperazinyl)acetonitrile (**2e**): yield, 88% (60.0 mg); light yellow solid. M.p. 102–103 °C. ^1^H NMR (400 MHz, CDCl_3_) δ 7.36 (d, *J* = 8.5 Hz, 2H), 7.16 (d, *J* = 8.5, 2H), 3.47 (s, 2H), 2.98 (t, *J* = 7.2, 2H), 2.63–2.48 (m, 10H). ^13^C NMR (101 MHz, CDCl_3_) δ 135.6, 132.0, 130.6, 119.8, 114.8, 57.1, 52.5, 51.6, 45.9, 30.9. HRMS (ESI) *m*/*z* [M + H]^+^ calcd for C_14_H_19_BrN_3_S, 340.0483; found, 340.0487.

2-(4-(2-((4-chlorophenyl)thio)ethyl)piperazinyl)acetonitrile (**2f**): yield, 87% (51.5 mg); white solid. M.p. 91–92 °C. ^1^H NMR (400 MHz, CDCl_3_) δ 7.29–7.23 (m, 4H), 3.51 (s, 2H), 3.02 (t, *J* = 7.2 Hz, 2H), 2.65–2.50 (m, 10H). ^13^C NMR (101 MHz, CDCl_3_) δ 134.8, 132.0, 130.5, 129.1, 114.8, 57.2, 52.5, 51.7, 45.9, 31.2. HRMS (ESI) *m*/*z* [M + H]^+^ calcd for C_14_H_19_ClN_3_S, 296.0988; found, 296.0992.

2-(4-(2-((4-fluorophenyl)thio)ethyl)piperazinyl)acetonitrile (**2g**): yield, 84% (47.0 mg); light yellow oil. ^1^H NMR (400 MHz, CDCl_3_) δ 7.36 (dd, *J* = 8.4, 5.3 Hz, 2H), 7.00 (t, *J* = 8.6 Hz, 2H), 3.50 (s, 2H), 2.99 (t, *J* = 7.2 Hz, 2H), 2.64–2.35 (m, 10H). ^13^C NMR (101 MHz, CDCl_3_) δ 161.7 (d, *J* = 246.3 Hz), 132.3 (d, *J* = 8.0 Hz), 131.0 (d, *J* = 3.3 Hz), 116.1 (d, *J* = 21.8 Hz), 114.8, 57.4, 52.4, 51.6, 45.8, 32.1. HRMS (ESI) *m*/*z* [M + H]^+^ calcd for C_14_H_19_FN_3_S, 280.1284; found, 280.1287.

2-(4-(2-((4-(trifluoromethyl)phenyl)thio)ethyl)piperazinyl)acetonitrile (**2h**): yield, 61% (40.2 mg); light yellow oil. ^1^H NMR (400 MHz, CDCl_3_) δ 7.48 (d, *J* = 8.0 Hz, 2H), 7.33 (d, *J* = 8.0 Hz), 3.48 (s, 2H), 3.08 (t, *J* = 7.3 Hz), 2.68–2.48 (m, 10H). ^13^C NMR (101 MHz, CDCl_3_) δ 141.1, 126.3 (q, *J* = 32.8 Hz), 126.3, 124.7 (q, *J* = 3.7 Hz), 123.1 (q, *J* = 271.8 Hz), 113.7, 55.7, 51.4, 50.6, 44.8, 28.9. HRMS (ESI) *m*/*z* [M + H]^+^ calcd for C_15_H_19_F_3_N_3_S, 330.1252; found, 330.1255.

2-(4-(2-((3-methoxyphenyl)thio)ethyl)piperazinyl)acetonitrile (**2i**): yield, 68% (39.6 mg); light yellow oil. ^1^H NMR (400 MHz, CDCl_3_) δ 7.16 (t, *J* = 8.0 Hz, 1H), 6.87 (d, *J* = 7.9 Hz, 1H), 6.84 (s, 1H), 6.68 (d, *J* = 8.2 Hz, 1H), 3.75 (s, 3H), 3.46 (s, 2H), 3.01 (t, *J* = 7.2 Hz, 2H), 2.67–2.47 (m, 10H). ^13^C NMR (101 MHz, CDCl_3_) δ 159.8, 137.6, 129.8, 120.9, 114.8, 114.3, 111.6, 57.3, 55.3, 52.5, 51.6, 45.8, 30.5. HRMS (ESI) *m*/*z* [M + H]^+^ calcd For C_15_H_22_N_3_OS, 292.1484; found, 292.1487.

2-(4-(2-(*m*-tolylthio)ethyl)piperazinyl)acetonitrile (**2j**): yield, 76% (41.9 mg); light yellow oil. ^1^H NMR (400 MHz, CDCl_3_) δ 7.17–7.08 (m, 3H), 6.97 (d, *J* = 7.0 Hz), 3.47 (s, 2H), 3.01 (t, *J* = 7.6 Hz, 2H), 2.65–2.48 (m, 10H), 2.30 (s, 3H). ^13^C NMR (101 MHz, CDCl_3_) δ 138.7, 135.9, 129.7, 128.8, 126.9, 126.0, 114.8, 57.4, 52.5, 51.7, 45.9, 30.7, 21.4. HRMS (ESI) *m*/*z* [M + H]^+^ calcd for C_15_H_22_N_3_S, 276.1534; found, 276.1538.

2-(4-(2-((3-bromophenyl)thio)ethyl)piperazinyl)acetonitrile (**2k**): yield, 86% (58.6 mg); light yellow oil. ^1^H NMR (400 MHz, CDCl_3_) δ 7.38 (s, 1H), 7.21 (d, *J* = 7.8 Hz, 1H), 7.17 (d, *J* = 7.9 Hz, 1H), 7.06 (t, *J* = 7.9 Hz, 1H), 3.43 (s, 2H), 2.97 (t, *J* = 7.2 Hz, 2H), 2.61–2.44 (m, 10H). ^13^C NMR (101 MHz, CDCl_3_) δ 139.0, 131.0, 130.3, 128.9, 127.2, 122.8, 114.8, 57.0, 52.4, 51.6, 45.9, 30.6. HRMS (ESI) *m*/*z* [M + H]^+^ calcd for C_14_H_19_BrN_3_S, 340.0483; found, 340.0487.

2-(4-(2-((3-chlorophenyl)thio)ethyl)piperazinyl)acetonitrile (**2l**): yield, 85% (50.3 mg); light yellow oil. ^1^H NMR (400 MHz, CDCl_3_) δ 7.23–7.21 (m, 1H), 7.13–7.10 (m, 2H), 7.07–7.04 (m, 1H), 3.42 (s, 2H), 2.97 (t, *J* = 7.2 Hz, 2H), 2.61–2.41 (m, 10H). ^13^C NMR (400 MHz, CDCl_3_) δ 138.7, 134.6, 130.0, 128.1, 126.6, 125.9, 114.8, 57.0, 52.4, 51.6, 45.9, 30.6. HRMS (ESI) *m*/*z* [M + H]^+^ calcd for C_14_H_19_ClN_3_S, 296.0988; found, 296.0992.

2-(4-(2-((3-fluorophenyl)thio)ethyl)piperazinyl)acetonitrile (**2m**): yield, 82% (45.8 mg); light yellow oil. ^1^H NMR (400 MHz, CDCl_3_) δ 7.21 (td, *J* = 8.0, 6.2 Hz, 1H), 7.05 (d, *J* = 7.9 Hz, 1H), 6.99 (t, *J* = 8.4 Hz, 1H), 6.83 (t, *J* = 8.4 Hz, 1H), 3.48 (s. 2H), 3.03 (t, *J* = 7.2 Hz, 2H), 2.67–2.44 (m, 10H). ^13^C NMR (101 MHz, CDCl_3_) δ162.9 (d, *J* = 247.9 Hz), 139.0 (d, *J* = 7.9 Hz), 130.2 (d, *J* = 8.6 Hz), 124.0 (d, *J* = 2.9 Hz), 115.1 (d, *J* = 23.1 Hz), 114.8, 112.7 (d, *J* = 21.2 Hz), 57.0, 52.5, 51.7, 45.9, 30.5. HRMS (ESI) *m*/*z* [M + H]^+^ calcd for C_14_H_19_FN_3_S, 280.1284; found, 280.1287.

2-(4-(2-((2-methoxyphenyl)thio)ethyl)piperazinyl)acetonitrile (**2n**): yield, 67% (39.0 mg); light yellow oil. ^1^H NMR (400 MHz, CDCl_3_) δ 7.20 (d, *J* = 7.6 Hz, 1H), 7.11 (t, *J* = 7.8 Hz, 1H), 6.84 (t, *J* = 7.5 Hz, 1H), 6.77 (d, *J* = 8.2 Hz, 1H), 3.80 (s, 3H), 3.41 (s, 2H), 2.94 (t, *J* = 7.2 Hz), 2.71–2.29 (m, 10H). ^13^C NMR (101 MHz, CDCl_3_) δ 157.4, 129.5, 127.3, 124.2, 121.0, 114.8, 110.5, 57.3, 55.8, 52.5, 51.7, 45.8, 29.2. HRMS (ESI) *m*/*z* [M + H]^+^ calcd for C_15_H_22_N_3_OS, 292.1484; found, 292.1487.

2-(4-(2-(*o*-tolylthio)ethyl)piperazinyl)acetonitrile (**2o**): yield, 70% (38.6 mg); light yellow oil. ^1^H NMR (400 MHz, CDCl_3_) δ 7.28 (d, *J* = 7.2 Hz, 1H), 7.19–7.12 (m, 2H), 7.09 (t, *J* = 7.2 Hz, 1H), 3.50 (s, 2H), 3.02 (t, *J* = 7.2 Hz), 2.71–2.47 (m, 10H), 2.37 (s, 3H). ^13^C NMR (101 MHz, CDCl_3_) δ 137.6, 135.5, 130.2, 127.8, 126.5, 125.8, 114.8, 57.2, 52.5, 51.7, 45.9, 30.1, 20.5. HRMS (ESI) *m*/*z* [M + H]^+^ calcd for C_15_H_22_N_3_S, 276.1534; found, 276.1538.

2-(4-(2-((2-bromophenyl)thio)ethyl)piperazinyl)acetonitrile (**2p**): yield, 85% (57.8 mg); light yellow oil. ^1^H NMR (400 MHz, CDCl_3_) δ 7.46 (d, *J* = 7.9 Hz, 1H), 7.21–7.18 (m, 2H), 6.99–6.92 (m, 1H), 3.43 (s, 2H), 2.99 (t *J* = 7.2 Hz, 2H), 2.66–2.44 (m, 10H). ^13^C NMR (101 MHz, CDCl_3_) δ 137.8, 133.0, 128.0, 127.9, 126.7, 123.6, 114.8, 56.7, 52.5, 51.6, 45.9, 30.2. HRMS (ESI) *m*/*z* [M + H]^+^ calcd for C_16_H_11_N_3_NaO_2_, C_14_H_19_BrN_3_S, 340.0483; found, 340.0487.

2-(4-(2-((2-chlorophenyl)thio)ethyl)piperazinyl)acetonitrile (**2q**): yield, 84% (49.7 mg); light yellow oil. ^1^H NMR (400 MHz, CDCl_3_) δ 7.28 (d, *J* = 7.8 Hz, 1H), 7.22 (d, *J* = 8.2 Hz, 1H), 7.14 (t, *J* = 7.5 Hz, 1H), 7.04 (t, *J* = 7.6 Hz, 1H), 3.42 (s, 2H), 2.99 (t, *J* = 7.2 Hz, 2H), 2.68–2.41 (m, 10H). ^13^C NMR (101 MHz, CDCl_3_) δ 135.7, 133.5, 129.7, 128.4, 127.2, 126.6, 114.8, 56.8, 52.5, 51.7, 45.9, 29.8. HRMS (ESI) *m*/*z* [M + H]^+^ calcd for C_14_H_19_ClN_3_S, 296.0988; found, 296.0992.

2-(4-(2-((2-fluorophenyl)thio)ethyl)piperazinyl)acetonitrile (**2r**): yield, 79% (44.1 mg); light yellow oil. ^1^H NMR (400 MHz, CDCl_3_) δ 7.38 (t, *J* = 7.6 Hz, 1H), 7.20 (dd, *J* = 13.1, 7.5 Hz, 1H), 7.12–7.00 (m, 2H), 3.48 (s, 2H), 3.01 (t, *J* = 7.2 Hz, 2H), 2.67–2.43 (m, 10H). ^13^C NMR (101 MHz, CDCl_3_) δ161.6 (d, *J* = 245.1 Hz), 132.3 (d, *J* = 1.8 Hz), 128.5 (d, *J* = 7.9 Hz), 123.0 (d, *J* = 17.6 Hz), 115.7 (d, *J* = 22.5 Hz), 114.8, 57.5, 52.4, 51.7, 45.9, 30.7. HRMS (ESI) *m*/*z* [M + H]^+^ calcd for C_14_H_19_FN_3_S, 280.1284; found, 280.1287.

2-(4-(2-((2,4-dimethylphenyl)thio)ethyl)piperazinyl)acetonitrile (**2s**): yield, 75% (43.4 mg); light yellow oil. ^1^H NMR (400 MHz, CDCl_3_) δ 7.21 (d, *J* = 7.9 Hz, 1H), 7.00 (s, 1H), 6.96 (d, *J* = 8.0 Hz, 1H), 3.49 (s, 2H), 2.96 (t, *J* = 7.2 Hz, 2H), 2.65–2.50 (m, 10H), 2.35 (s, 3H), 2.28 (s, 3H). ^13^C NMR (101 MHz, CDCl_3_) δ 138.3, 136.1, 131.6, 131.2, 129.4, 127.2, 114.8, 57.4, 52.5, 51.7, 45.9, 30.8, 20.9, 20.5. HRMS (ESI) *m*/*z* [M + H]^+^ calcd for C_16_H_24_N_3_S, 290.1691; found, 290.1694.

2-(4-(2-((2,5-dimethylphenyl)thio)ethyl)piperazinyl)acetonitrile (**2t**): yield, 74% (42.8 mg); light yellow oil. ^1^H NMR (400 MHz, CDCl_3_) δ 7.09 (s, 1H), 7.04 (d, *J* = 7.6 Hz, 1H), 6.89 (d, *J* = 7.6 Hz, 1H), 3.48 (s, 2H), 3.00 (t, *J* = 7.2 Hz, 2H), 2.67–2.51 (m, 10H), 2.32 (s, 3H), 2.29 (s, 3H). ^13^C NMR (101 MHz, CDCl_3_) δ 136.0, 135.1, 134.6, 130.0, 128.7, 126.6, 114.8, 57.2, 52.5, 51.7, 45.9, 30.2, 21.1, 20.0. HRMS (ESI) *m*/*z* [M + H]^+^ calcd for C_16_H_24_N_3_S, 290.1691; found, 290.1694.

2-(4-(2-((3,5-dimethylphenyl)thio)ethyl)piperazinyl)acetonitrile (**2u**): yield, 78% (45.2 mg); light yellow iol. ^1^H NMR (400 MHz, CDCl_3_) δ 6.94 (s, 2H), 6.78 (s, 1H), 3.47 (s, 2H), 3.00 (t, *J* = 7.6 Hz, 2H), 2.65–2.46 (m, 10H), 2.26 (s, 6H). ^13^C NMR (101 MHz, CDCl_3_) δ 138.5, 135.7, 127.9, 126.7, 114.8, 57.5, 52.5, 51.7, 45.9, 30.7, 21.3. HRMS (ESI) *m*/*z* [M + H]^+^ calcd for C_16_H_24_N_3_S, 290.1691; found, 290.1694.

2-(4-(2-(naphthalen-2-ylthio)ethyl)piperazinyl)acetonitrile (**2v**): yield, 57%, (35.5 mg), light yellow oil. ^1^H NMR (400 MHz, CDCl_3_) δ 7.79–7.70 (m, 4H), 7.49–7.39 (m, 3H), 3.46 (s, 2H), 3.13 (t, *J* = 7.6 Hz, 2H), 2.71–2.48 (m, 10H). ^13^C NMR (101 MHz, CDCl_3_) δ 133.8, 131.7, 128.5, 127.8, 127.3, 127.1, 126.7, 125.7, 114.8, 57.3, 52.5, 51.7, 45.9, 30.7. HRMS (ESI) *m*/*z* [M + H]^+^ calcd for C_18_H_22_N_3_S, 312.1534; found, 312.1538.

2-(4-(2-(pyridin-2-ylthio)ethyl)piperazinyl)acetonitrile (**2w**): yield, 84% (44.1 mg); light yellow oil. ^1^H NMR (400 MHz, CDCl_3_) δ 8.40 (d, *J* = 4.0 Hz, 1H), 7.46 (t, *J* = 7.5 Hz, 1H), 7.17 (d, *J* = 8.0 Hz, 1H), 6.96 (t, *J* = 5.6 Hz, 1H), 3.50 (s, 2H), 3.31 (t, *J* = 7.3 Hz, 2H), 2.74–2.66 (m, 2H), 2.61 (br s, 8H). ^13^C NMR (101 MHz, CDCl_3_) δ 158.6, 149.4, 135.9, 122.2, 119.4, 114.9, 57.5, 52.4, 51.6, 45.8, 26.9. HRMS (ESI) *m*/*z* [M + H]^+^ calcd for C_13_H_19_N_4_S, 263.1331; found, 263.1334.

2-(4-(2-(thiophen-2-ylthio)ethyl)piperazinyl)acetonitrile (**2x**): yield, 76% (40.6 mg); light yellow oil. ^1^H NMR (400 MHz, CDCl_3_) δ 7.32 (d, *J* = 5.3 Hz, 1H), 7.11 (d, *J* = 3.5 Hz, 1H), 6.95 (dd, *J* = 5.3, 3.6 Hz, 1H), 3.48 (s, 2H), 2.89 (t, *J* = 7.2 Hz, 2H), 2.64–2.45 (m, 10H). ^13^C NMR (101 MHz, CDCl_3_) δ 134.2, 133.7, 129.3, 127.6, 114.8, 57.5, 52.5, 51.7, 45.9, 35.8. HRMS (ESI) *m*/*z* [M + H]^+^ calcd For C_12_H_18_N_3_S_2_, 268.0942; found, 268.0946.

2-(4-(2-((2-methylfuran-3-yl)thio)ethyl)piperazinyl)acetonitrile (**2y**): yield, 45% (23.9 mg); light yellow oil. ^1^H NMR (400 MHz, CDCl_3_) δ 7.21 (d, *J* = 2.0 Hz, 1H), 6.27 (d, *J* = 1.8 Hz, 1H), 3.43 (s, 2H), 2.66 (t, *J* = 7.2 Hz, 2H), 2.57–2.41 (m, 10H), 2.27 (s, 3H). ^13^C NMR (101 MHz, CDCl_3_) δ 154.9, 140.6, 115.0, 114.7, 110.0, 57.9, 52.5, 51.7, 45.9, 32.8, 11.9. HRMS (ESI) *m*/*z* [M + H]^+^ calcd for C_13_H_20_N_3_OS, 266.1327; found, 266.1331.

2-(4-(2-(benzo[*d*]thiazol-2-ylthio)ethyl)piperazinyl)acetonitrile (**2z**): yield, 60% (38.2 mg); light yellow solid. M.p. 77–78 °C. ^1^H NMR (400 MHz, CDCl_3_) δ 7.77 (d, *J* = 8.0 Hz, 1H), 7.67 (d, *J* = 7.9 Hz, 1H), 7.32 (t, *J* = 7.7 Hz, 1H), 7.20 (t, *J* = 7.6 Hz, 1H), 3.44 (t, *J* = 7.1 Hz, 2H), 3.42 (s, 2H), 2.74 (t, *J* = 7.2 Hz, 2H), 2.54 (br s, 8H). **^13^C** NMR (101 MHz, CDCl_3_) δ 167.0, 153.2, 135.2, 126.1, 124.3, 121.4, 121.0, 114.8, 56.7, 52.4, 51.7, 45.9, 30.8. HRMS (ESI) *m*/*z* [M + H]^+^ calcd for C_15_H_19_N_4_S_2_, 319.1051; found, 319.1055.

2-(4-(2-(benzo[*d*]oxazol-2-ylthio)ethyl)piperazinyl)acetonitrile (**2aa**): yield, 68% (41.1 mg); light yellow solid. M.p. 57–58 °C. ^1^H NMR (400 MHz, CDCl_3_) δ 7.50 (d, *J* = 7.3 Hz, 1H), 7.35 (d, *J* = 7.5 Hz, 1H), 7.17 (tt, *J* = 7.5, 6.4 Hz, 2H), 3.41 (s, 2H), 3.38 (t, *J* = 6.9 Hz, 2H), 2.74 (t, *J* = 6.9 Hz, 2H), 2.54 (br s, 8H). ^13^C NMR (101 MHz, CDCl_3_) δ 165.2, 151.8, 141.9, 124.3, 123.9, 118.3, 114.8, 109.9, 56.5, 52.3, 51.7, 45.9, 30.0. HRMS (ESI) *m*/*z* [M + H]^+^ calcd for C_15_H_19_N_4_OS, 303.1280; found, 303.1283.

2-(4-(2-((1*H*-benzo[*d*]imidazol-2-yl)thio)ethyl)piperazinyl)acetonitrile (**2ab**): yield, 71% (42.8 mg); light yellow solid. M.p. 139–140 °C. ^1^H NMR (400 MHz, CDCl_3_) δ 10.66 (s, 1H), 7.54 (dd, *J* = 6.0, 3.2 Hz, 2H), 7.10 (dd, *J* = 6.0, 3.2 Hz, 2H), 3.48 (s, 2H), 3.14 (t, *J* = 5.2 Hz, 2H), 2.81 (t, *J* = 5.6 Hz, 2H), 2.63 (br s, 8H). ^13^C NMR (101 MHz, CDCl_3_) δ 151.0, 139.7, 122.1, 114.7, 114.3, 60.0, 52.8, 51.4, 45.8, 29.7. HRMS (ESI) *m*/*z* [M + H]^+^ calcd for C_15_H_20_N_5_S, 302.1439; found, 302.1443.

2-(4-(2-(hexylthio)ethyl)piperazinyl)acetonitrile (**2ac**): yield, 51%, (27.5 mg), light yellow oil. ^1^H NMR (400 MHz, CDCl_3_) δ 3.45 (s, 2H), 2.60–2.43 (m, 14H), 1.55–1.47 (m, 2H), 1.34–1.18 (m, 6H), 0.82 (t, *J* = 6.8 Hz, 3H). ^13^C NMR (101 MHz, CDCl_3_) δ 114.8, 58.2, 52.5, 51.7, 45.8, 32.4, 31.4, 29.7, 29.1, 28.5, 22.5, 14.1. HRMS (ESI) *m*/*z* [M + H]^+^ calcd for C_14_H_28_N_3_S, 270.2004; found, 270.2007.

2-(4-(2-(phenylselanyl)ethyl)piperazinl)acetonitrile (**2ad**): yield, 80%, (49.3 mg), light yellow oil. ^1^H NMR (400 MHz, CDCl_3_) δ 7.49 (d, *J* = 7.8 Hz, 2H), 7.29–7.20 (m, 3H), 3.48 (s, 2H), 3.02 (t, *J* = 7.2 Hz, 2H), 2.70 (t, *J* = 8.0 Hz, 2H), 2.63–2.49 (m, 8H). ^13^C NMR (101 MHz, CDCl_3_) δ 132.4, 130.3, 129.1, 126.9, 114.8, 58.1, 52.3, 51.7, 45.9, 24.8. HRMS (ESI) *m*/*z* [M + H]^+^ calcd for C_14_H_20_N_3_Se, 310.0817; found, 310.0819.

#### 3.2.2. Experimental Procedures for the Synthesis of ACAT-1 Inhibitors **5a** and **5b**

(a) Method 1:

Compound **2ab** (60.3 mg, 0.2 mmol), KOH (44.9 mg, 0.8 mmol) and tert-butanol (2.0 mL) were mixed in a 50 mL Teflon screw-cap sealed tube. The mixture was vigorously stirred under N_2_ atmosphere for 1 h at 110 °C (oil bath). The reaction mixture was diluted with CH_2_Cl_2_/MeOH (10 mL, 1/1) and filtered. Solvent was removed, and the crude product was purified on a silica gel column eluted with CH_2_Cl_2_/MeOH (3:1 *v*/*v*) to afford the product **4b** in 75% yield (47.9 mg). White solid. M.p. 208–209 °C. ^1^H NMR (400 MHz, CD_3_OD/CDCl_3_ (1:1)) δ 7.49 (dd, *J* = 5.8, 3.1 Hz, 2H), 7.21 (dd, *J* = 6.0, 3.1 Hz, 2H), 3.33 (t, *J* = 6.4 Hz, 2H), 3.07 (s, 2H), 2.88 (t, *J* = 6.4 Hz, 2H), 2.68 (s, 8H).

Compound **4b** (6.4 mg, 0.02 mmol), 2,6-diisopropylaniline (3.5 mg, 0.02 mmol), K_2_S_2_O_8_ (16.2 mg, 0.06 mmol) and MeCN (3 mL) were mixed in a 50 mL Teflon screw-cap sealed tube. The mixture was vigorously stirred under N_2_ atmosphere for 8 h at 85 °C (oil bath). The reaction mixture was diluted with CH_2_Cl_2_/MeOH (10 mL, 1/1) and filtered. Solvent was removed, and the crude product was purified on a silica gel column eluted with CH_2_Cl_2_/MeOH (10:1 *v*/*v*) to afford product **5a** as a white solid (10%, 1.0 mg). ^1^H NMR (400 MHz, DMSO-d_6_) δ 12.57 (s, 1H), 9.13 (s, 1H), 7.40 (s, 2H), 7.24–7.19 (m, 1H), 7.12 (s, 1H), 7.10 (s, 1H), 7.08 (dd, *J* = 5.9, 3.2 Hz, 2H), 3.41 (t, *J* = 6.9 Hz, 2H), 3.11 (s, 2H), 3.04–2.93 (m, 2H), 2.68 (t, *J* = 6.9 Hz, 2H), 2.55 (br s, 8H), 1.08 (d, *J* = 6.9 Hz, 12H). ^13^C NMR (101 MHz, DMSO-d_6_) δ 169.9, 151.1, 146.4, 133.0, 128.0, 123.3, 121.8, 61. 9, 57.8, 53.6, 52.9, 29.3, 28.6, 24.0.

Compound **4b** (6.4 mg, 0.02 mmol), 6-methyl-2,4-bis(methylthio)pyridin-3-amine (4.0 mg, 0.02 mmol), K_2_S_2_O_8_ (16.2 mg, 0.06 mmol), and MeCN (3 mL) were mixed in a 50 mL Teflon screw-cap sealed tube. The mixture was vigorously stirred under N_2_ atmosphere for 8 h at 85 °C (oil bath). The reaction mixture was diluted with CH_2_Cl_2_/MeOH (10 mL, 1/1) and filtered. Solvent was removed, and the crude product was purified on a silica gel column eluted with CH_2_Cl_2_/MeOH (10:1 *v*/*v*) to afford the product **5b** as a white solid (12%, 1.2 mg). ^1^H NMR (400 MHz, CDCl_3_) δ 8.53 (s, 1H), 7.48 (dd, *J* = 5.4, 2.9 Hz, 2H), 7.16 (dd, *J* = 5.9, 3.1 Hz, 2H), 6.61 (s, 1H), 3.33–3.25 (m, 4H), 2.92–2.70 (m, 10H), 2.51 (s, 3H), 2.46 (s, 3H), 2.38 (s, 3H). ^13^C NMR (101 MHz, CDCl_3_) δ169.2, 156.9, 156.2, 151.0, 148.4, 139.8, 122.9, 121.9, 114.2, 113.7, 61.6, 59.4, 53.5, 53.2, 29.7, 24.5, 14.0, 12.9.

(b) Method 2:

1*H*-benzo[d]imidazole-2-thiol **1ab** (30.0 mg, 0.2 mmol), CAABC (59.1 mg, 0.3 mmol), Cs_2_CO_3_ (195.5 mg, 0.6mmol) and EtOH (1 mL) were mixed in a 50 mL Teflon screw-cap sealed tube. The mixture was vigorously stirred under air atmosphere for 3 h at 100 °C (oil bath). The reaction mixture was diluted with CH_2_Cl_2_/MeOH (10 mL, 1/1) and filtered. Solvent was removed, and the crude product was purified on a silica gel column eluted with CH_2_Cl_2_/MeOH (6:1 *v*/*v*) to afford product **3b** in 80% yield (51.9 mg). White solid. M.p. 117–118 oC. ^1^H NMR (400 MHz, CD_3_OD/CDCl_3_ (1:1)) δ 7.56 (d, *J* = 2.8 Hz, 2H), 7.31–7.25 (m, 2H), 3.51–3.45 (m, 2H), 3.21–3.18 (m, 4H), 2.95–2.89 (m, 2H), 2.84 (br s, 4H). ^13^C NMR (101 MHz, CD_3_OD/CDCl_3_ (1:1)) δ 154.6, 143.2, 126.1, 117.9, 62.5, 56.1, 48.5, 33.3.

A mixture of compound **3b** (162.2 mg, 0.5 mmol), 2-bromo-*N*-(2,6-diisopropylphenyl) acetamide (149.1 mg, 0.5 mmol), and K_2_CO_3_ (414.6 mg, 3 mmol) in MeCN (10 mL) was stirred under air atmosphere for 12 h at room temperature. The reaction mixture was filtered, and solvent was removed under reduced pressure. The crude product was purified on a silica gel column eluted with CH_2_Cl_2_/MeOH (10:1 *v*/*v*) to afford compound **5a** in 60% yield (143.9 mg).

A mixture of compound **3b** (162.2 mg, 0.5 mmol), 2-bromo-*N*-(6-methyl-2,4-bis(methylthio)pyridin-3-yl)acetamide (160.6 mg, 0.5 mmol) and K_2_CO_3_ (414.6 mg, 3 mmol) in MeCN (10 mL) was stirred under air atmosphere for 12 h at room temperature. The reaction mixture was filtered, and solvent was removed under reduced pressure. The crude product was purified on a silica gel column eluted with CH_2_Cl_2_/MeOH (6:1 *v*/*v*) to afford compound **5b** in 75% yield (188.5 mg).

## 4. Conclusions

In summary, we have described a simple and eco-friendly method for the synthesis of 2-(4-(2-(phenylthio)ethyl)piperazinyl)acetonitriles (**2**) in one-pot. Further reactions produced aqueous soluble acyl-CoA:cholesterol *O*-acyltransferase-1 (ACAT-1) inhibitors such as [K-604]. The advantage of this method lies in green solvent, water and dioxygen insensitivity, less-odor disulfide source, and easy purification. Gram level of reaction with purity over 90% makes this method more practical. The methodology would prove very useful in the area of medicinal chemistry.

## Figures and Tables

**Table 1:** Optimization of the formation of compound **2a** ^a^.

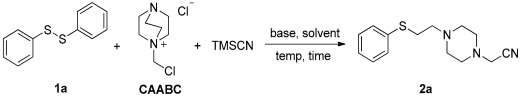					
En	Base	Solvent	Temp/°C	Time/h	Yield ^b^
1	**Cs_2_CO_3_**	**EtOH**	**100**	**3**	**90%**
2	**K_2_CO_3_**	EtOH	100	3	73%
3	**Na_2_CO_3_**	EtOH	100	3	57%
4	**KOH**	EtOH	100	3	35%
5	***^t^*BuOK**	EtOH	100	3	30%
6	Cs_2_CO_3_	**EtOH/H_2_O** (**7:3**)	100	3	55%
7	Cs_2_CO_3_	**H_2_O**	100	3	trace
8	Cs_2_CO_3_	**MeOH**	100	3	75%
9	Cs_2_CO_3_	**DMF**	100	3	none
10	Cs_2_CO_3_	**DMSO**	100	3	none
11	Cs_2_CO_3_	EtOH	**120**	3	88%
12	Cs_2_CO_3_	EtOH	**80**	3	82%
13	Cs_2_CO_3_	EtOH	100	**6**	87%
14	Cs_2_CO_3_	EtOH	100	**1**	72%

^a^ Reaction conditions: **1a** (0.1 mmol), 1-(chloromethyl)-4-aza-1-azoniabicyclo[2.2.2]octane chloride (0.2 mmol), TMSCN (0.22 mmol), base (0.6 mmol), solvent (1 mL), air atmosphere. ^b^ Yields were determined by ^1^H NMR analysis of the crude product using 1,3,5-trimethoxybenzene as the internal standard; Temp = temperature; En = entry.

## Data Availability

The data underlying this study are available in the published article and its online Supplementary Material.

## Supplementary Materials

The following supporting information can be downloaded at: https://www.mdpi.com/article/10.3390/molecules29163723/s1. Experimental procedures, Characterization data of products, X-ray structure determinations, Crystallographic data of compounds, and ^1^H NMR and ^13^C NMR spectra of compounds. CCDC 2328305, CCDC 2328309, CCDC 2,328,313 and CCDC 2,328,311 contain the supplementary crystallographic data for this paper. These data can be obtained free of charge via www.ccdc.cam.ac.uk/data_request/cif (accessed on 5 June 2024), or by emailing da-ta_request@ccdc.cam.ac.uk, or by contacting The Cambridge Crystallographic Data Centre, 12 Union Road, Cambridge CB2 1EZ, UK; fax: +44-1223-336033.
