# Supplementary material for: Chain Extension of Piperazine in Ethanol: Synthesis of 2-(4-(2-(Phenylthio)ethyl)piperazinyl)acetonitriles and ACAT-1 Inhibitors
Source: Molecules. 2024 Aug 6;29(16):3723. doi: 10.3390/molecules29163723 (PMC11356844; doi:10.3390/molecules29163723)

Supporting Information

Chain Extension of Piperazine in Ethanol: Synthesis of 2-(4-(2-(Phenylthio)ethyl)piperazinyl)acetonitriles and ACAT-1 Inhibitors

Ying Huang,[a,b,c] Tingyu Zhu[a,b,c], Yinghua Li,[a,*] and Deguang Huang*,[a]

[a] *State Key Laboratory of Structural Chemistry, Fujian Institute of Research on the Structure of Matter, Chinese Academy of Sciences, Fuzhou, Fujian 350002, China*

[b] *Fujian Normal University, College of Chemistry and Materials Science, Fuzhou 350007, China.*

[c] *Fujian College, University of Chinese Academy of Science, Fuzhou, Fujian 350002, China*

**To whom correspondence should be addressed. E-mail: dhuang@fjirsm.ac.cn*

Table of contents:

1. General information ·· S2

2. Experimental procedures ·· S2

3. Characterization data of products ·· S8

4. X-ray structure determinations ·· S18

5. Crystallographic data of compounds ·· S19

6. References ·· S20

7. ^{1}H NMR and ^{13}C NMR spectra of compounds ·· S21

---

1. General information

Chemicals. Unless otherwise stated, all commercial-grade chemicals were used without further purification. 6-Methyl-2,4-bis(methylthio)-pyridin-3-amine,[1] 2-bromo-*N*-(2,6-diisopropylphenyl) acetamide[2] and 2-bromo-*N*-(6-methyl-2,4-bis(methylthio)pyridin-3-yl)acetamide[2] were prepared according to the reported methods.

General Physical Measurements. ^{1}H NMR and ^{13}C NMR spectra were recorded on Bruker Avance III (400 MHz) and chemical shifts were expressed in δ ppm values with reference to tetramethylsilane (TMS) as internal standard. HR-MS (ESI) spectra were obtained on a Bruker Impact II quardrupole time off light mass spectrometer. The single crystal diffraction data were collected on an Oxford Diffraction Supernova dual diffractometer equipped with an Oxford Cryostream 700 low-temperature apparatus.

2. Experimental procedures

2.1 General procedure[3] for the synthesis of disulfides 1.

A pad of silica gel (200 - 300 mesh) (5.5 g) with water (2.5 mL) in a 200 mL flask was stirred for 10 minutes. CH_2Cl_2 (30 mL) was added and the mixture was stirred for a further 5 minutes. Thiols (5 mmol) was added, followed by an addition of Br_2 (2.5 mmol) in a period of 30 minutes. The mixture was stirred for 12 h, filtered and the silica gel was washed with CH_2Cl_2 (3 × 20 mL). The organic solutions were combined and dried over $MgSO_4$. Solvent was removed *in vacuo* to afford the products as pale yellow oil or solid. The yields of products are shown in Table S1. The characterization data are given in pages S8-S11; ^{1}H NMR spectra are presented in pages S21-S30.

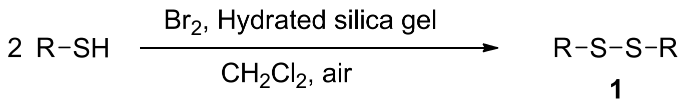

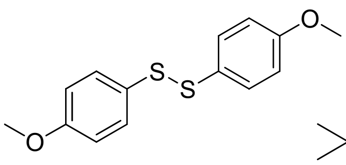

1b (98%)

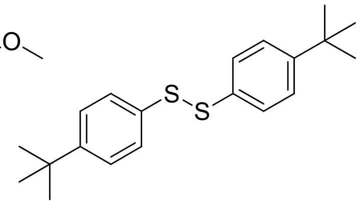

1c (97%)

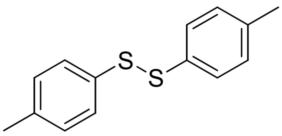

1d (98%)

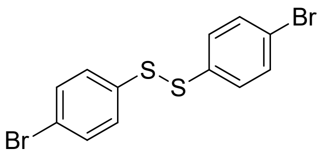

1e (96%)

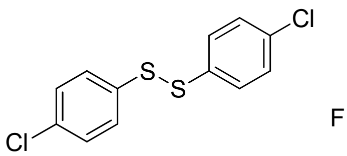

1f (97%)

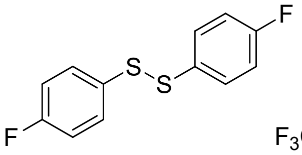

1g (96%)

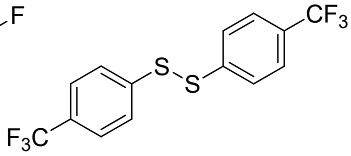

1h (93%)

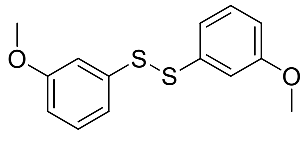

1i (95%)

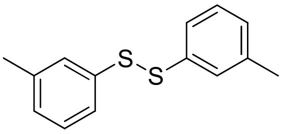

1j (94%)

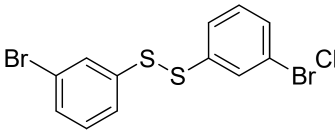

1k (93%)

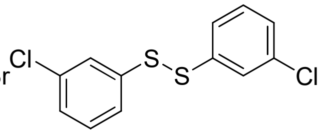

1l (94%)

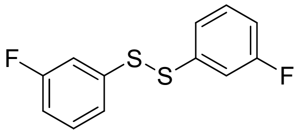

1m (92%)

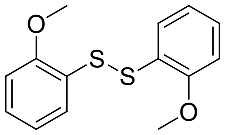

1n (94%)

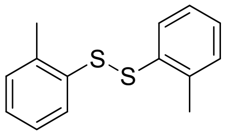

1o (96%)

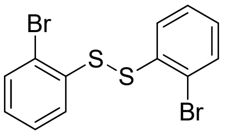

1p (93%)

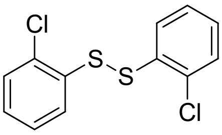

1q (94%)

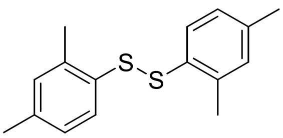

1s (93%)

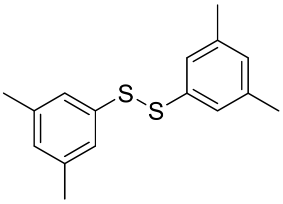

1u (95%)

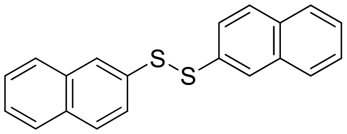

1v (92%)

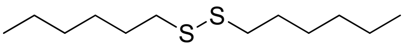

1ac (91%)

Table S1. Preparation of disulfides **1**.

2.2 Experimental procedures[4] for the synthesis of 1-(chloromethyl)-4-aza-1-azonia bicyclo[2.2.2]octane chloride (CAABC), 1-(cyanomethyl)-4-aza-1-azonia bicyclo[2.2.2]octane chloride (CYAABC), and 1-ethyl-4-aza-1-azoniabicyclo[2.2.2]octane bromide (EAABB).

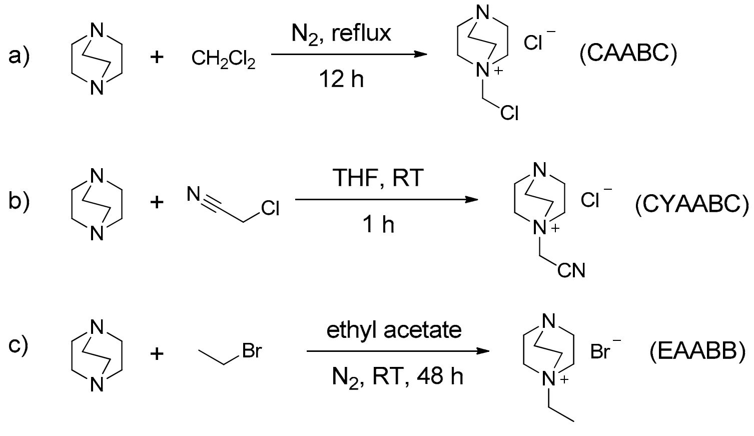

a) A solution of 1,4-diazabicyclo[2.2.2]octane (DABCO) (11.2 g, 0.1 mol) in CH_2Cl_2 (30 mL) was refluxed for 12 h under N_2 atmosphere. The resulting precipitate was collected, washed with CH_2Cl_2 (2 × 20 mL), and dried *in vacuo* to yield the product 1-(chloromethyl)-4-aza-1-azonia bicyclo[2.2.2]octane chloride in 96% yield (18.9 g). ^{1}H NMR (400 MHz, D_2O) δ 5.03 (s, 2H), 3.46 (t, J = 7.2 Hz, 6H), 3.16 (t, J = 7.6 Hz, 6H). ^{13}C NMR (101 MHz, D_2O) δ 68.3, 51.2, 44.0.

b) A mixture of DABCO (1.12 g, 10 mmol) and chloroacetonitrile (0.755 g, 10 mmol) in THF (50 mL) was stirred for 1 h at room temperature. The resulting precipitate was collected, washed with petroleum ether (2 × 10 mL), and dried *in vacuo* to yield the product 1-(cyanomethyl)-4-aza-1-azonia bicyclo[2.2.2]octane chloride in 96% yield (1.8 g). ^{1}H NMR (400 MHz, DMSO-d_6) δ 5.12 (s, 2H), 3.48 (t, J = 7.2 Hz, 6H), 3.06 (t, J = 7.6 Hz, 6H). **^{13}C** NMR (101 MHz, DMSO-d_6) δ 112.3, 53.0, 50.6, 45.0.

c) To a solution of DCBCO (560.9 mg, 5 mmol) in ethyl acetate (5 mL) was added 1-bromoethane (544.9 mg, 5 mol) slowly under N_2 atmosphere. The solution was stirred at room temperature for 48 h. The resulting precipitate was collected, washed with ethyl acetate (2 × 10 mL), and dried *in vacuo* to yield the product 1-ethyl-4-aza-1-azonia bicyclo[2.2.2]octane chloride in 95% yield (1.05 g). ^{1}H NMR (400 MHz, DMSO-d_6) δ 3.34 - 3.20 (m, 8H), 3.10 - 2.90 (m, 6H), 1.21 (t, J = 5.5 Hz, 3H). ^{13}C NMR (101 MHz, DMSO-d_6) δ 59.1, 51.4, 45.2, 7.8.

2.3 General procedure for the synthesis of compounds 2.

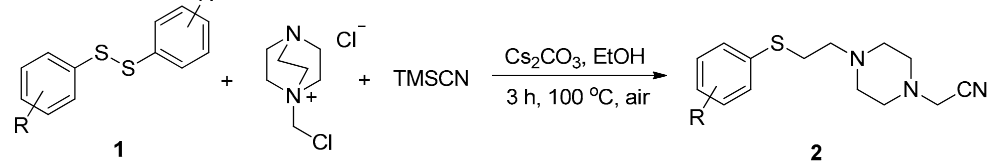

Disulfides **1** (0.1 mmol), 1-(chloromethyl)-4-aza-1-azonia bicyclo[2.2.2]octane chloride (CAABC) (0.2 mmol), trimethylsilyl cyanide (0.22 mmol), Cs_2CO_3 (0.6 mmol) and EtOH (1 mL) were mixed in a 50 mL Teflon screw-cap sealed tube. The mixture was vigorously stirred under air atmosphere for 3 h at 100 °C (oil bath). After cooling to room temperature, the reaction mixture was filtered. The precipitate was washed with EtOH (2 mL). The organic layers were combined and

flashed through a pad of silica gel (3 mL) in pipette eluted with petroleum ether/EtOH (10:1 to 5:1 v/v) (10 mL) to yield the products **2**. The yields and the characterization data of products are shown in pages S11-S17; ^{1}H NMR spectra are presented in pages S31-S60.

2.4 Experimental procedure for the scale-up reaction.

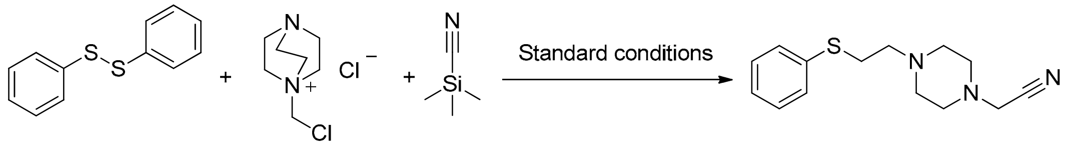

1a (0.55 g, 2.5 mmol) (0.99 g, 5 mmol) **2a** (90%, 1.18 g)

Diphenyl disulfide **1a** (0.55 g, 2.5 mmol), 1-(chloromethyl)-4-aza-1-azonia bicyclo[2.2.2]octane chloride (CAABC) (0.99 g, 5.0 mmol), trimethylsilyl cyanide (TMSCN, 0.69 mL, 5.5 mmol), Cs_2CO_3 (4.89 g, 15 mmol) and EtOH (25 mL) were mixed in a 250 mL flask equipped with a condenser. The mixture was refluxed under N_2 atmosphere for 3 h (oil bath). After cooling to room temperature, the reaction mixture was filtered. The filtrate was concentrated to 3 mL under reduced pressure. The concentrated solution was flashed through a silica gel column (10 g), and the column was eluted with petroleum ether/EtOH (10:1 to 5:1 v/v) to yield product **2a** in 90% (1.18 g).

2.5 Experimental procedures for the reactions shown in the Scheme 3.

a) Diphenyl disulfide **1a** (21.8 mg, 0.1 mmol), CAABC (39.4 mg, 0.2 mmol), trimethylsilyl cyanide (TMSCN, 28 μL, 0.22 mmol), Cs_2CO_3 (195.5 mg, 0.6 mmol) and EtOH (1 mL) were mixed in a 50 mL Teflon screw-cap sealed tube. The mixture was vigorously stirred for 3 h at 100 °C (oil bath) under N_2 atmosphere. After cooling to room temperature, the reaction mixture was filtered. The precipitate was washed with EtOH (2 mL). The organic layers were combined and flashed through a pad of silica gel (3 mL) in pipette eluted with petroleum ether/EtOH (5:1 v/v) (10 mL) to yield product **2a** in 91% yield (47.6 mg).

b) Thiophenol (22.0 mg, 0.2 mmol), CAABC (39.4 mg, 0.2 mmol), trimethylsilyl cyanide (TMSCN, 28 μL, 0.22 mmol), Cs_2CO_3 (195.5 mg, 0.6 mmol) and EtOH (1 mL) were mixed in a 50 mL Teflon screw-cap sealed tube. The mixture was vigorously stirred under air atmosphere for 3 h at 100 °C (oil bath). After cooling to room temperature, the reaction mixture was filtered. The precipitate was washed with EtOH (2 mL). The organic layers were combined and flashed through a pad of silica gel (3 mL) in pipette, eluted with petroleum ether/EtOH (5:1 *v/v*) to afford product **2a** in 89% yield (46.5 mg).

c) Diphenyl disulfide **1a** (21.8 mg, 0.1 mmol), CAABC (59.1 mg, 0.3 mmol), Cs_2CO_3 (195.5 mg, 0.6 mmol) and EtOH (1 mL) were mixed in a 50 mL Teflon screw-cap sealed tube. The mixture was vigorously stirred under air atmosphere for 3 h at 100 °C (oil bath). The reaction mixture was diluted with CH_2Cl_2/MeOH (10 mL, 1/1) and filtered. Solvent was removed and the crude product was purified on a silica gel column eluted with CH_2Cl_2/MeOH (6:1 *v/v*) to afford product **3a** as a white solid (50.0 mg, 88%). M.p. 109 - 110 °C. ^{1}H NMR (400 MHz, $CDCl_3$) δ 7.35 (d, J = 7.6 Hz, 2H), 7.30 (t, J = 7.2 Hz, 2H), 7.20 (t, J = 7.2 Hz, 1H), 5.89 (s, 2H), 3.15 - 3.10 (m, 4H), 3.05 (t, J = 7.2 Hz, 2H), 2.71 - 2.65 (m, 6H). ^{13}C NMR (101 MHz, $CDCl_3$) δ 135.9, 129.5, 129.0, 126.3, 57.0, 49.4, 43.6, 31.0.

d) Diphenyl disulfide **1a** (21.8 mg, 0.1 mmol), CYAABC (37.5 mg, 0.2 mmol) and EtOH (1 mL) were mixed in a 50 mL Teflon screw-cap sealed tube. The mixture was vigorously stirred at 100 °C (oil bath) under air atmosphere for 3 h. After cooling to room temperature, the reaction mixture was filtered and solvent was removed under reduced pressure. The crude product was purified on a silica gel column eluted with CH_2Cl_2/MeOH (10:1 *v/v*) to afford product **4a** as a white solid (16.8 mg, 30%). M.p. 120 - 121 °C. ^1H NMR (400 MHz, $CDCl_3$) δ 7.34 (d, *J* = 7.4 Hz, 2H), 7.28 (t, *J* = 7.6 Hz, 2H), 7.18 (t, *J* = 7.2 Hz, 1H), 7.00 (s, 1H), 6.17 (s, 1H), 3.06 (t, *J* = 7.2 Hz, 2H), 3.00 (s, 2H), 2.68 - 2.52 (m, 10H).

e) Diphenyl disulfide **1a** (21.8 mg, 0.1 mmol), DABCO (22.4 mg, 0.2 mmol), trimethylsilyl cyanide (TMSCN, 28 μL, 0.22 mmol), Cs_2CO_3 (195.5 mg, 0.6 mmol) and EtOH (1 mL) were mixed in a 50 mL Teflon screw-cap sealed tube. The mixture was vigorously stirred under air atmosphere for 3 h at 100 °C (oil bath). No desired product was found on the TLC plate.

f) Diphenyl disulfide **1a** (21.8 mg, 0.1 mmol), EAABC (44.2 mg, 0.2 mmol), trimethylsilyl cyanide (TMSCN, 28 μL, 0.22 mmol), Cs_2CO_3 (195.5 mg, 0.6 mmol) and EtOH (1 mL) were mixed in a 50 mL Teflon screw-cap sealed tube. The mixture was vigorously stirred under air atmosphere for 3 h at 100 °C (oil bath). No desired product was found on the TLC plate.

g) CAABC (39.4 mg, 0.2 mmol), trimethylsilyl cyanide (TMSCN, 28 μL, 0.22 mmol), Cs_2CO_3 (195.5 mg, 0.6 mmol) and EtOH (1 mL) were mixed in a 50 mL Teflon screw-cap sealed tube. The mixture was vigorously stirred under air atmosphere for 3 h at 100 °C (oil bath). No new product was found with the recovery of CAABC checked by ^1H NMR measurement.

2.6 Experimental procedures for the synthesis of ACAT-1 inhibitors 5a and 5b

a) Synthesis of compound 4b[5]: compound **2ab** (60.3 mg, 0.2 mmol), KOH (44.9 mg, 0.8 mmol) and *tert*-butanol (2.0 mL) were mixed in a 50 mL Teflon screw-cap sealed tube. The mixture was vigorously stirred under N_2 atmosphere for 1 h at 110 °C (oil bath). The reaction mixture was diluted with CH_2Cl_2/MeOH (10 mL, 1/1) and filtered. Solvent was removed and the crude product was purified on a silica gel column eluted with CH_2Cl_2/MeOH (3:1 *v/v*) to afford the product **4b** in 75% yield (47.9 mg). White solid. M.p. 208 - 209 °C. ^1H NMR (400 MHz, $CD_3OD/CDCl_3$ (1:1)) δ 7.49 (dd, *J* = 5.8, 3.1 Hz, 2H), 7.21 (dd, *J* = 6.0, 3.1 Hz, 2H), 3.33 (t, *J* = 6.4 Hz, 2H), 3.07 (s, 2H), 2.88 (t, *J* = 6.4 Hz, 2H), 2.68 (s, 8H).

Synthesis of compound 5a[6]: compound **4b** (6.4 mg, 0.02 mmol), 2,6-diisopropylaniline (3.5 mg, 0.02 mmol), $K_2S_2O_8$ (16.2 mg, 0.06 mmol) and MeCN (3 mL) were mixed in a 50 mL Teflon screw-cap sealed tube. The mixture was vigorously stirred under N_2 atmosphere for 8 h at 85 °C (oil bath). The reaction mixture was diluted with CH_2Cl_2/MeOH (10 mL, 1/1) and filtered. Solvent was removed and the crude product was purified on a silica gel column eluted with CH_2Cl_2/MeOH (10:1 *v/v*) to afford product **5a** as a white solid (10%, 1.0 mg). ^1H NMR (400 MHz, DMSO-d_6) δ 12.57 (s, 1H), 9.13 (s, 1H), 7.40 (s, 2H), 7.24 - 7.19 (m, 1H), 7.12 (s, 1H), 7.10 (s, 1H), 7.08 (dd, *J* = 5.9, 3.2 Hz, 2H), 3.41 (t, *J* = 6.9 Hz, 2H), 3.11 (s, 2H), 3.04 - 2.93 (m, 2H), 2.68 (t, *J* = 6.9 Hz, 2H), 2.55 (br s, 8H), 1.08 (d, *J* = 6.9 Hz, 12H). ^{13}C NMR (101 MHz, DMSO-d_6) δ 169.9, 151.1, 146.4, 133.0, 128.0, 123.3, 121.8, 61. 9, 57.8, 53.6, 52.9, 29.3, 28.6, 24.0.

Synthesis of compound 5b: compound **4b** (6.4 mg, 0.02 mmol), 6-methyl-2,4-bis(methylthio) pyridin-3-amine (4.0 mg, 0.02 mmol), $K_2S_2O_8$ (16.2 mg, 0.06 mmol), and MeCN (3 mL) were mixed in a 50 mL Teflon screw-cap sealed tube. The mixture was vigorously stirred under N_2 atmosphere for 8 h at 85 °C (oil bath). The reaction mixture was diluted with CH_2Cl_2/MeOH (10 mL, 1/1) and filtered. Solvent was removed and the crude product was purified on a silica gel column eluted with CH_2Cl_2/MeOH (10:1 *v/v*) to afford the product **5b** as a white solid (12%, 1.2 mg). ^1H NMR (400 MHz, $CDCl_3$) δ 8.53 (s, 1H), 7.48 (dd, *J* = 5.4, 2.9 Hz, 2H), 7.16 (dd, *J* = 5.9, 3.1 Hz, 2H), 6.61 (s, 1H), 3.33 - 3.25 (m, 4H), 2.92 - 2.70 (m, 10H), 2.51 (s, 3H), 2.46 (s, 3H), 2.38 (s, 3H). ^{13}C NMR (101 MHz, $CDCl_3$) δ169.2, 156.9, 156.2, 151.0, 148.4, 139.8, 122.9, 121.9, 114.2, 113.7, 61.6, 59.4, 53.5, 53.2, 29.7, 24.5, 14.0, 12.9.

b) Synthesis of compound 3b: 1*H*-benzo[*d*]imidazole-2-thiol **1ab** (30.0 mg, 0.2 mmol), CAABC (59.1 mg, 0.3 mmol), Cs_2CO_3 (195.5 mg, 0.6mmol) and EtOH (1 mL) were mixed in a 50 mL Teflon screw-cap sealed tube. The mixture was vigorously stirred under air atmosphere for 3 h at 100 °C (oil bath). The reaction mixture was diluted with CH_2Cl_2/MeOH (10 mL, 1/1) and filtered. Solvent was removed and the crude product was purified on a silica gel column eluted with CH_2Cl_2/MeOH (6:1 *v/v*) to afford product **3b** in 80% yield (51.9 mg). White solid. M.p. 117 - 118 ºC. ^1H NMR (400 MHz, $CD_3OD/CDCl_3$ (1:1)) δ 7.56 (d, *J* = 2.8 Hz, 2H), 7.31 - 7.25 (m, 2H), 3.51 - 3.45 (m, 2H), 3.21 - 3.18 (m, 4H), 2.95 - 2.89 (m, 2H), 2.84 (br s, 4H). ^{13}C NMR (101 MHz, $CD_3OD/CDCl_3$ (1:1)) δ 154.6, 143.2, 126.1, 117.9, 62.5, 56.1, 48.5, 33.3.

Synthesis of compound 5a[7]: A mixture of compound **3b** (162.2 mg, 0.5 mmol), 2-bromo-*N*-(2,6-diisopropylphenyl) acetamide (149.1 mg, 0.5 mmol), and K_2CO_3 (414.6 mg, 3 mmol) in MeCN (10 mL) was stirred under air atmosphere for 12 hours at room temperature. The reaction mixture was filtered and solvent was removed under reduced pressure. The crude product was purified on a silica gel column eluted with CH_2Cl_2/MeOH (10:1 *v/v*) to afford compound **5a** in 60% yield (143.9 mg).

Synthesis of compound 5b: A mixture of compound **3b** (162.2 mg, 0.5 mmol), 2-bromo-*N*-(6-methyl-2,4-bis(methylthio)pyridin-3-yl)acetamide (160.6 mg, 0.5 mmol) and K_2CO_3 (414.6 mg, 3 mmol) in MeCN (10 mL) was stirred under air atmosphere for 12 hours at room temperature. The reaction mixture was filtered and solvent was removed under reduced pressure. The crude product was purified on a silica gel column eluted with CH_2Cl_2/MeOH (6:1 *v/v*) to afford compound **5b** in 75% yield (188.5 mg).

3. Characterization Data of Products

3.1 Characterization data of the products 1

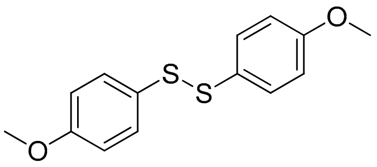

Bis(4-methoxyphenyl) disulfide (**1b**): yield, 98% (0.68 g); light yellow solid. ^{1}H NMR (400 MHz, $CDCl_3$) δ 7.42 (d, J = 8.8 Hz, 4H), 6.85 (d, J = 8.7 Hz, 4H), 3.80 (s, 6H).

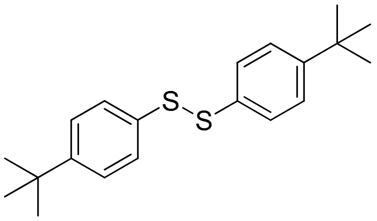

Bis(4-tert-butylphenyl) disulfide (**1c**): yield, 95% (0.79 g); light yellow solid. ^{1}H NMR (400 MHz, $CDCl_3$) δ 7.47 (d, J = 8.5 Hz, 4H), 7.35 (d, J = 8.5 Hz, 4H), 1.32 (s, 18H).

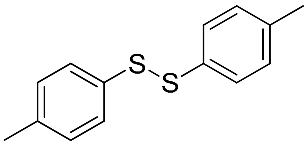

Bis(4-methylphenyl) disulfide (**1d**): yield, 98% (0.60 g); light yellow solid. ^{1}H NMR (400 MHz, $CDCl_3$) δ 7.36 (d, J = 8.2 Hz, 4H), 7.07 (d, J = 8.0 Hz, 4H), 2.28 (s, 6H).

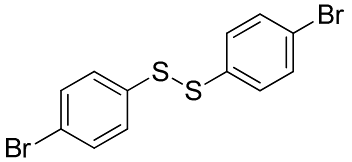

Bis(4-bromophenyl) disulfide (**1e**): yield, 96% (0.90 g); light yellow solid. ^{1}H NMR (400 MHz, $CDCl_3$) δ 7.33 (d, J = 8.5 Hz, 4H), 7.24 (d, J = 8.5 Hz, 4H).

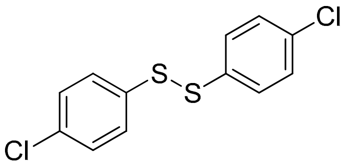

Bis(4-chlorophenyl) disulfide (**1f**): yield, 97% (0.70 g); light yellow solid. ^{1}H NMR (400 MHz, $CDCl_3$) δ 7.31 (d, J = 8.5 Hz, 4H), 7.18 (d, J = 8.5 Hz, 4H).

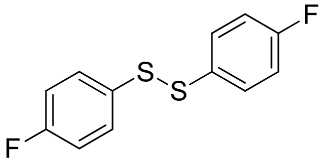

Bis(4-fluorophenyl)disulfide (**1g**): yield, 96% (0.61 g); light yellow, solid. ^{1}H NMR (400 MHz, $CDCl_3$) δ 7.48 - 7.42 (m, 4H), 7.02 (t, J = 8.6 Hz, 4H).

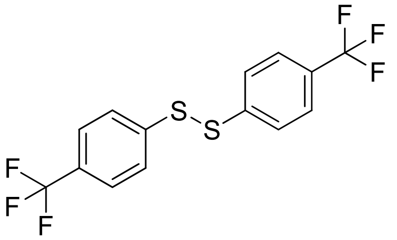

Bis(4-(trifluoromethyl)phenyl) disulfide (**1h**): yield, 93% (0.82 g); light yellow solid. ^{1}H NMR (400 MHz, $CDCl_3$) δ 7.60 - 7.53 (m, 8H).

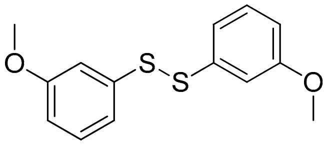

Bis(3-methoxyphenyl) disulfide (**1i**): yield, 95% (0.66 g); light yellow solid. ^{1}H NMR (400 MHz, $CDCl_3$) δ 7.17 (t, J = 8.2 Hz, 2H), 7.08 - 7.04 (m, 4H), 6.72 (d, J = 7.3 Hz, 2H), 3.71 (s, 6H).

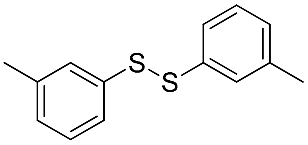

Bis(3-methylphenyl) disulfide (**1j**): yield, 94% (0.58 g); light yellow solid. ^{1}H NMR (400 MHz, $CDCl_3$) δ 7.33 - 7.30 (m, 4H), 7.20 (t, J = 8.0 Hz, 2H), 7.04 (d, J = 7.6 Hz, 2H), 2.33 (s, 6H).

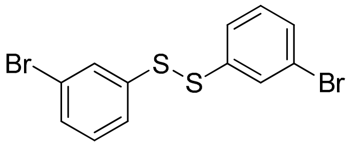

Bis(3-bromophenyl)disulfide (**1k**): yield, 93% (0.87 g); light yellow oil. ^{1}H NMR (400 MHz, $CDCl_3$) δ 7.62 (s, 2H), 7.41 - 7.35 (m, 4H), 7.18 (t, J = 7.9 Hz, 2H).

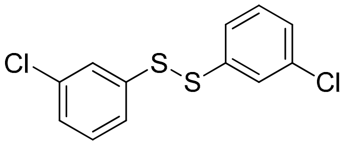

Bis(3-chlorophenyl) disulfide (**1l**): yield, 94% (0.67 g); light yellow solid. ^{1}H NMR (400 MHz, $CDCl_3$) δ 7.38 (s, 2H), 7.25 (d, J = 7.2 Hz, 2H), 7.16 - 7.09 (m, 4H).

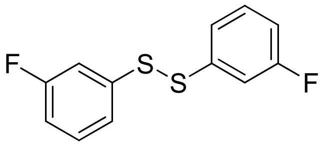

Bis(3-fluorophenyl)disulfide (**1m**): yield, 92% (0.58 g); light yellow solid. ^{1}H NMR (400 MHz, $CDCl_3$) δ 7.30 - 7.20 (m, 6H), 6.94 - 6.89 (m, 2H).

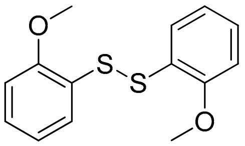

Bis(2-methoxyphenyl) disulfide (**1n**): yield, 94% (0.65 g); light yellow solid. ^{1}H NMR (400 MHz, $CDCl_3$) δ 7.44 (d, *J* = 7.7 Hz, 2H), 7.09 (t, *J* = 7.7 Hz, 2H), 6.82 (t, *J* = 7.6 Hz, 2H), 6.76 (d, *J* = 8.1 Hz, 2H), 3.80(s, 6H).

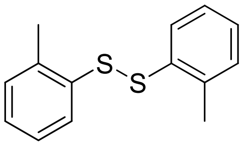

Bis(2-methylphenyl) disulfide (**1o**): yield, 96% (0.59 g); light yellow solid. ^{1}H NMR (400 MHz, $CDCl_3$) δ 7.56 - 7.52 (m, 2H), 7.21 - 7.12 (m, 6H), 2.46 (s, 6H).

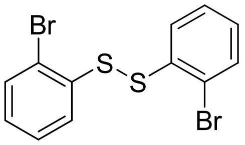

Bis(2-bromophenyl)disulfide (**1p**): yield, 93% (0.87 g); light yellow solid. ^{1}H NMR (400 MHz, $CDCl_3$) δ 7.47 - 7.42 (m, 4H), 7.19 (t, *J* = 7.7 Hz, 2H), 7.00 (t, *J* = 7.6 Hz, 2H).

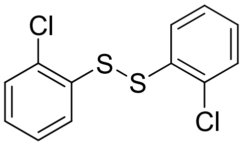

Bis(2-chlorophenyl) disulfide (**1q**): yield, 94% (0.67 g); light yellow solid. ^{1}H NMR (400 MHz, $CDCl_3$) δ 7.54 (dd, *J* = 7.9, 1.4 Hz, 2H), 7.35 (dd, *J* = 7.8, 1.2 Hz, 2H), 7.25 - 7.10 (m, 4H).

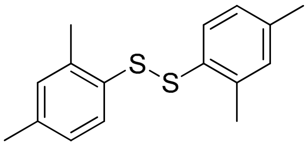

Bis(2,4-dimethylphenyl)disulfide (**1s**): yield, 93% (0.64 g); light yellow solid. ^{1}H NMR (400 MHz, $CDCl_3$) δ 7.28 (d, *J* = 7.9 Hz, 2H), 6.89 (s, 2H), 6.83 (d, *J* = 7.9 Hz, 2H), 2.28 (s, 6H), 2.19 (s, 6H).

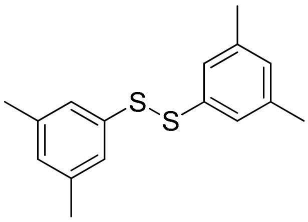

Bis(3,5-dimethylphenyl)disulfide (**1u**): yield, 95% (0.65 g); light yellow solid. ^{1}H NMR (400 MHz, $CDCl_3$) δ 7.11 (s, 4H), 6.83 (s, 2H), 2.26 (s, 12H).

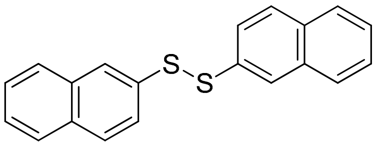

Bis(2-naphthyl)disulfide (**1v**): yield, 92% (0.73 g); light yellow solid. ^{1}H NMR (400 MHz, $CDCl_3$) δ 7.90 (s, 2H), 7.72 - 7.68 (m, 4H), 7.65 (dd, *J* = 6.1, 2.9 Hz, 2H), 7.54 (dd, *J* = 8.7, 1.8 Hz, 2H), 7.41 - 7.34 (m, 4H).

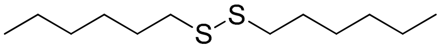

dihexyl disulfide (**1ac**): yield, 91% (0.53 g); light yellow oil. ^{1}H NMR (400 MHz, $CDCl_3$) δ 2.67 (t, *J* = 7.6 Hz, 4H), 1.70 - 1.62 (m, 4H), 1.42 - 1.24 (m, 12H), 0.88 (t, *J* = 6.8 Hz, 6H).

3.2 Characterization data of the products 2

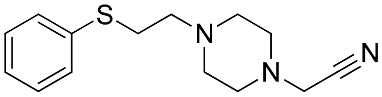

2-(4-(2-(phenylthio)ethyl)piperazinyl)acetonitrile (**2a**): yield, 90% (47.1 mg); light yellow oil. ^{1}H NMR (400 MHz, $CDCl_3$) δ 7.32 (d, *J* = 7.3 Hz, 2H), 7.26 (t, *J* = 7.6 Hz, 2H), 7.15 (t, *J* = 7.2 Hz, 1H), 3.46 (s, 2H), 3.02 (t, *J* = 7.2 Hz, 2H), 2.65 - 2.47 (m, 10H). ^{13}C NMR (101 MHz, $CDCl_3$) δ 136.3, 129.0, 128.97, 126.0, 114.8, 57.4, 52.5, 51.7, 45.9, 30.8. HRMS (ESI) m/z $[M+H]^+$ calcd For $C_{14}H_{20}N_3S$, 262.1372; found, 262.1375.

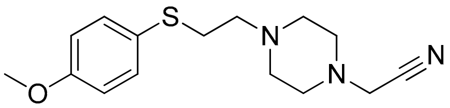

2-(4-(2-((4-methoxyphenyl)thio)ethyl)piperazinyl)acetonitrile (**2b**): yield, 76% (44.3 mg); light yellow solid. M.p. 88 - 89 ºC. ^{1}H NMR (400 MHz, $CDCl_3$) δ 7.32 (d, *J* = 8.7 Hz, 2H), 6.81 (d, *J* = 8.7 Hz, 2H), 3.76 (s, 3H), 3.46 (s, 2H), 2.90 (t, *J* = 7.6 Hz, 2H), 2.59 - 2.42 (m, 10H). ^{13}C NMR (101 MHz, $CDCl_3$) δ 159.0, 133.3, 126.1, 114.8, 114.6, 57.7, 55.4, 52.5, 51.7, 45.9, 32.8. HRMS (ESI) *m/z* $[M+H]^+$ calcd For $C_{15}H_{22}N_3OS$, 292.1484; found, 292.1487.

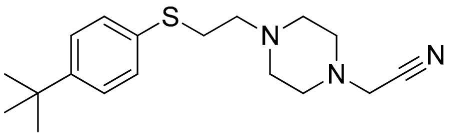

2-(4-(2-((4-(*tert*-butyl)phenyl)thio)ethyl)piperazinyl)acetonitrile (**2c**): yield, 77% (48.9 mg); light yellow oil. ^{1}H NMR (400 MHz, $CDCl_3$) δ 7.33 - 7.26 (m, 4H), 3.49 (s, 2H), 3.02 (t, *J* = 7.2 Hz, 2H), 2.66 - 2.49 (m, 10H), 1.30 (s, 9H). ^{13}C NMR (101 MHz, $CDCl_3$) δ 149.4, 132.6, 129.3, 126.0, 114.8, 57.5, 52.5, 51.7, 45.9, 34.5, 31.3, 31.2. HRMS (ESI) *m/z* $[M+H]^+$ calcd For $C_{18}H_{28}N_3S$, 318.2004; found, 318.2007.

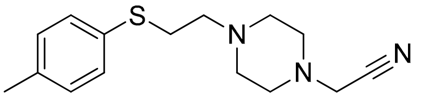

2-(4-(2-(*p*-tolylthio)ethyl)piperazinyl)acetonitrile (**2d**): yield, 78% (43.0 mg); light yellow oil. ^{1}H NMR (400 MHz, CDCl$_3$) δ 7.23 (d, *J* = 8.1 Hz, 2H), 7.07 (d, *J* = 8.0 Hz, 2H), 3.46 (s, 2H), 2.97 (t, *J* = 7.6 Hz, 2H), 2.64 - 2.45 (m, 10H), 2.29 (s, 3H). ^{13}C NMR (101 MHz, CDCl$_3$) δ 136.3, 132.3, 130.0, 114.8, 57.5, 52.5, 51.7, 45.9, 31.4, 21.1. HRMS (ESI) *m/z* [M+H]$^+$ calcd For $C_{15}H_{22}N_3S$, 276.1534; found, 276.1538.

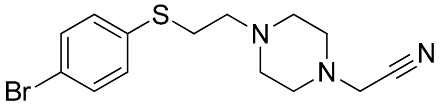

2-(4-(2-((4-bromophenyl)thio)ethyl)piperazinyl)acetonitrile (**2e**): yield, 88% (60.0 mg); light yellow solid. M.p. 102 - 103 ºC. ^{1}H NMR (400 MHz, CDCl$_3$) δ 7.36 (d, *J* = 8.5 Hz, 2H), 7.16 (d, *J* = 8.5, 2H), 3.47 (s, 2H), 2.98 (t, *J* = 7.2, 2H), 2.63 - 2.48 (m, 10H). ^{13}C NMR (101 MHz, CDCl$_3$) δ 135.6, 132.0, 130.6, 119.8, 114.8, 57.1, 52.5, 51.6, 45.9, 30.9. HRMS (ESI) *m/z* [M+H]$^+$ calcd For $C_{14}H_{19}BrN_3S$, 340.0483; found, 340.0487.

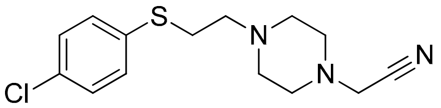

2-(4-(2-((4-chlorophenyl)thio)ethyl)piperazinyl)acetonitrile (**2f**): yield, 87% (51.5 mg); white solid. M.p. 91 - 92 ºC. ^{1}H NMR (400 MHz, CDCl$_3$) δ 7.29 - 7.23 (m, 4H), 3.51 (s, 2H), 3.02 (t, *J* = 7.2 Hz, 2H), 2.65 - 2.50 (m, 10H). ^{13}C NMR (101 MHz, CDCl$_3$) δ 134.8, 132.0, 130.5, 129.1, 114.8, 57.2, 52.5, 51.7, 45.9, 31.2. HRMS (ESI) *m/z* [M+H]$^+$ calcd For $C_{14}H_{19}ClN_3S$, 296.0988; found, 296.0992.

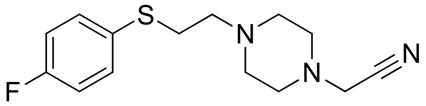

2-(4-(2-((4-fluorophenyl)thio)ethyl)piperazinyl)acetonitrile (**2g**): yield, 84% (47.0 mg); light yellow oil. ^{1}H NMR (400 MHz, CDCl$_3$) δ 7.36 (dd, *J* = 8.4, 5.3 Hz, 2H), 7.00 (t, *J* = 8.6 Hz, 2H), 3.50 (s, 2H), 2.99 (t, *J* = 7.2 Hz, 2H), 2.64 - 2.35 (m, 10H). ^{13}C NMR (101 MHz, CDCl$_3$) δ 161.7 (d, *J* = 246.3 Hz), 132.3 (d, *J* = 8.0 Hz), 131.0 (d, *J* = 3.3 Hz), 116.1 (d, *J* = 21.8 Hz), 114.8, 57.4, 52.4, 51.6, 45.8, 32.1. HRMS (ESI) *m/z* [M+H]$^+$ calcd For $C_{14}H_{19}FN_3S$, 280.1284; found, 280.1287.

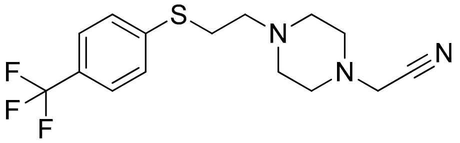

2-(4-(2-((4-(trifluoromethyl)phenyl)thio)ethyl)piperazinyl)acetonitrile (**2h**): yield, 61% (40.2 mg); light yellow oil. ^{1}H NMR (400 MHz, CDCl$_3$) δ 7.48 (d, *J* = 8.0 Hz, 2H), 7.33 (d, *J* = 8.0 Hz), 3.48 (s, 2H), 3.08 (t, *J* = 7.3 Hz), 2.68 - 2.48 (m, 10H). ^{13}C NMR (101 MHz, CDCl$_3$) δ 141.1, 126.3 (q, *J* = 32.8 Hz), 126.3, 124.7 (q, *J* = 3.7 Hz), 123.1 (q, *J* = 271.8 Hz), 113.7, 55.7, 51.4, 50.6, 44.8, 28.9. HRMS (ESI) *m/z* [M+H]$^+$ calcd For $C_{15}H_{19}F_3N_3S$, 330.1252; found, 330.1255.

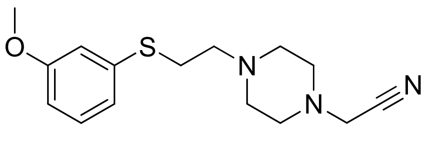

2-(4-(2-((3-methoxyphenyl)thio)ethyl)piperazinyl)acetonitrile (**2i**): yield, 68% (39.6 mg); light yellow oil. ^{1}H NMR (400 MHz, $CDCl_3$) δ 7.16 (t, *J* = 8.0 Hz, 1H), 6.87 (d, *J* = 7.9 Hz, 1H), 6.84 (s, 1H), 6.68 (d, *J* = 8.2 Hz, 1H), 3.75 (s, 3H), 3.46 (s, 2H), 3.01 (t, *J* = 7.2 Hz, 2H), 2.67 - 2.47 (m, 10H). ^{13}C NMR (101 MHz, $CDCl_3$) δ 159.8, 137.6, 129.8, 120.9, 114.8, 114.3, 111.6, 57.3, 55.3, 52.5, 51.6, 45.8, 30.5. HRMS (ESI) *m/z* $[M+H]^+$ calcd For $C_{15}H_{22}N_3OS$, 292.1484; found, 292.1487.

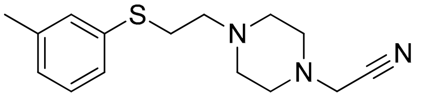

2-(4-(2-(*m*-tolylthio)ethyl)piperazinyl)acetonitrile (**2j**): yield, 76% (41.9 mg); light yellow oil. ^{1}H NMR (400 MHz, $CDCl_3$) δ 7.17 - 7.08 (m, 3H), 6.97 (d, *J* = 7.0 Hz), 3.47 (s, 2H), 3.01 (t, *J* = 7.6 Hz, 2H), 2.65 - 2.48 (m, 10H), 2.30 (s, 3H). ^{13}C NMR (101 MHz, $CDCl_3$) δ 138.7, 135.9, 129.7, 128.8, 126.9, 126.0, 114.8, 57.4, 52.5, 51.7, 45.9, 30.7, 21.4. HRMS (ESI) *m/z* $[M+H]^+$ calcd For $C_{15}H_{22}N_3S$, 276.1534; found, 276.1538.

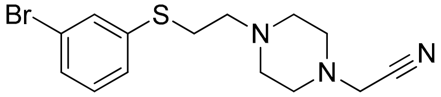

2-(4-(2-((3-bromophenyl)thio)ethyl)piperazinyl)acetonitrile (**2k**): yield, 86% (58.6 mg); light yellow oil. ^{1}H NMR (400 MHz, $CDCl_3$) δ 7.38 (s, 1H), 7.21 (d, *J* = 7.8 Hz, 1H), 7.17 (d, *J* = 7.9 Hz, 1H), 7.06 (t, *J* = 7.9 Hz, 1H), 3.43 (s, 2H), 2.97 (t, *J* = 7.2 Hz, 2H), 2.61 - 2.44 (m, 10H). ^{13}C NMR (101 MHz, $CDCl_3$) δ 139.0, 131.0, 130.3, 128.9, 127.2, 122.8, 114.8, 57.0, 52.4, 51.6, 45.9, 30.6. HRMS (ESI) *m/z* $[M+H]^+$ calcd For $C_{14}H_{19}BrN_3S$, 340.0483; found, 340.0487.

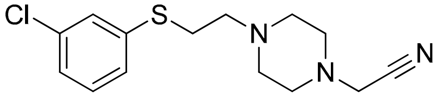

2-(4-(2-((3-chlorophenyl)thio)ethyl)piperazinyl)acetonitrile (**2l**): yield, 85% (50.3 mg); light yellow oil. ^{1}H NMR (400 MHz, $CDCl_3$) δ 7.23 - 7.21 (m, 1H), 7.13 - 7.10 (m, 2H), 7.07 - 7.04 (m, 1H), 3.42 (s, 2H), 2.97 (t, *J* = 7.2 Hz, 2H), 2.61 -2.41 (m, 10H). ^{13}C NMR (400 MHz, $CDCl_3$) δ 138.7, 134.6, 130.0, 128.1, 126.6, 125.9, 114.8, 57.0, 52.4, 51.6, 45.9, 30.6. HRMS (ESI) *m/z* $[M+H]^+$ calcd For $C_{14}H_{19}ClN_3S$, 296.0988; found, 296.0992.

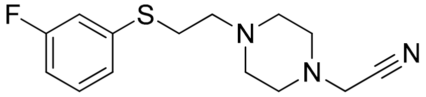

2-(4-(2-((3-fluorophenyl)thio)ethyl)piperazinyl)acetonitrile (**2m**): yield, 82% (45.8 mg); light yellow oil. ^{1}H NMR (400 MHz, $CDCl_3$) δ 7.21 (td, *J* = 8.0, 6.2 Hz, 1H), 7.05 (d, *J* = 7.9 Hz, 1H), 6.99 (t, *J* = 8.4 Hz, 1H), 6.83 (t, *J* = 8.4 Hz, 1H), 3.48 (s. 2H), 3.03 (t, *J* = 7.2 Hz, 2H), 2.67 - 2.44 (m, 10H). ^{13}C NMR (101 MHz, $CDCl_3$) δ162.9 (d, *J* = 247.9 Hz), 139.0 (d, *J* = 7.9 Hz), 130.2 (d, *J*

= 8.6 Hz), 124.0 (d, J = 2.9 Hz), 115.1 (d, J = 23.1 Hz), 114.8, 112.7 (d, J = 21.2 Hz), 57.0, 52.5, 51.7, 45.9, 30.5. HRMS (ESI) *m/z* $[M+H]^+$ calcd For $C_{14}H_{19}FN_3S$, 280.1284; found, 280.1287.

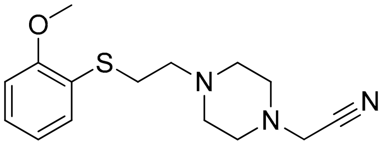

2-(4-(2-((2-methoxyphenyl)thio)ethyl)piperazinyl)acetonitrile (**2n**): yield, 67% (39.0 mg); light yellow oil. ^{1}H NMR (400 MHz, $CDCl_3$) δ 7.20 (d, J = 7.6 Hz, 1H), 7.11 (t, J = 7.8 Hz, 1H), 6.84 (t, J = 7.5 Hz, 1H), 6.77 (d, J = 8.2 Hz, 1H), 3.80 (s, 3H), 3.41 (s, 2H), 2.94 (t, J = 7.2 Hz), 2.71 - 2.29 (m, 10H). ^{13}C NMR (101 MHz, $CDCl_3$) δ 157.4, 129.5, 127.3, 124.2, 121.0, 114.8, 110.5, 57.3, 55.8, 52.5, 51.7, 45.8, 29.2. HRMS (ESI) *m/z* $[M+H]^+$ calcd For $C_{15}H_{22}N_3OS$, 292.1484; found, 292.1487.

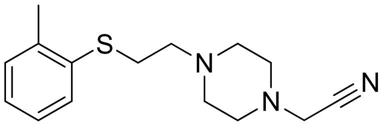

2-(4-(2-(*o*-tolylthio)ethyl)piperazinyl)acetonitrile (**2o**): yield, 70% (38.6 mg); light yellow oil. ^{1}H NMR (400 MHz, $CDCl_3$) δ 7.28 (d, J = 7.2 Hz, 1H), 7.19 - 7.12 (m, 2H), 7.09 (t, J = 7.2 Hz, 1H), 3.50 (s, 2H), 3.02 (t, J = 7.2 Hz), 2.71 - 2.47 (m, 10H), 2.37 (s, 3H). ^{13}C NMR (101 MHz, $CDCl_3$) δ 137.6, 135.5, 130.2, 127.8, 126.5, 125.8, 114.8, 57.2, 52.5, 51.7, 45.9, 30.1, 20.5. HRMS (ESI) *m/z* $[M+H]^+$ calcd For $C_{15}H_{22}N_3S$, 276.1534; found, 276.1538.

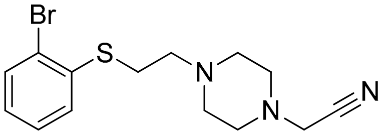

2-(4-(2-((2-bromophenyl)thio)ethyl)piperazinyl)acetonitrile (**2p**): yield, 85% (57.8 mg); light yellow oil. ^{1}H NMR (400 MHz, $CDCl_3$) δ 7.46 (d, J = 7.9 Hz, 1H), 7.21 - 7.18 (m, 2H), 6.99 - 6.92 (m, 1H), 3.43 (s, 2H), 2.99 (t J = 7.2 Hz, 2H), 2.66 - 2.44 (m, 10H). ^{13}C NMR (101 MHz, $CDCl_3$) δ 137.8, 133.0, 128.0, 127.9, 126.7, 123.6, 114.8, 56.7, 52.5, 51.6, 45.9, 30.2. HRMS (ESI) *m/z* $[M+H]^+$ calcd For $C_{16}H_{11}N_3NaO_2$, $C_{14}H_{19}BrN_3S$, 340.0483; found, 340.0487.

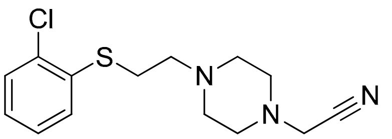

2-(4-(2-((2-chlorophenyl)thio)ethyl)piperazinyl)acetonitrile (**2q**): yield, 84% (49.7 mg); light yellow oil. ^{1}H NMR (400 MHz, $CDCl_3$) δ 7.28 (d, J = 7.8 Hz, 1H), 7.22 (d, J = 8.2 Hz, 1H), 7.14 (t, J = 7.5 Hz, 1H), 7.04 (t, J = 7.6 Hz, 1H), 3.42 (s, 2H), 2.99 (t, J = 7.2 Hz, 2H), 2.68 - 2.41 (m, 10H). ^{13}C NMR (101 MHz, $CDCl_3$) δ 135.7, 133.5, 129.7, 128.4, 127.2, 126.6, 114.8, 56.8, 52.5, 51.7, 45.9, 29.8. HRMS (ESI) *m/z* $[M+H]^+$ calcd For $C_{14}H_{19}ClN_3S$, 296.0988; found, 296.0992.

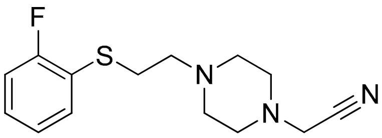

2-(4-(2-((2-fluorophenyl)thio)ethyl)piperazinyl)acetonitrile (**2r**): yield, 79% (44.1 mg); light yellow oil. ^{1}H NMR (400 MHz, $CDCl_3$) δ 7.38 (t, *J* = 7.6 Hz, 1H), 7.20 (dd, *J* = 13.1, 7.5 Hz, 1H), 7.12 - 7.00 (m, 2H), 3.48 (s, 2H), 3.01 (t, *J* = 7.2 Hz, 2H), 2.67 - 2.43 (m, 10H). ^{13}C NMR (101 MHz, $CDCl_3$) δ161.6 (d, *J* = 245.1 Hz), 132.3 (d, *J* = 1.8 Hz), 128.5 (d, *J* = 7.9 Hz), 123.0 (d, *J* = 17.6 Hz), 115.7 (d, *J* = 22.5 Hz), 114.8, 57.5, 52.4, 51.7, 45.9, 30.7. HRMS (ESI) *m/z* $[M+H]^+$ calcd For $C_{14}H_{19}FN_3S$, 280.1284; found, 280.1287.

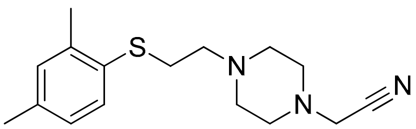

2-(4-(2-((2,4-dimethylphenyl)thio)ethyl)piperazinyl)acetonitrile (**2s**): yield, 75% (43.4 mg); light yellow oil. ^{1}H NMR (400 MHz, $CDCl_3$) δ 7.21 (d, *J* = 7.9 Hz, 1H), 7.00 (s, 1H), 6.96 (d, *J* = 8.0 Hz, 1H), 3.49 (s, 2H), 2.96 (t, *J* = 7.2 Hz, 2H), 2.65 - 2.50 (m, 10H), 2.35 (s, 3H), 2.28 (s, 3H). ^{13}C NMR (101 MHz, $CDCl_3$) δ 138.3, 136.1, 131.6, 131.2, 129.4, 127.2, 114.8, 57.4, 52.5, 51.7, 45.9, 30.8, 20.9, 20.5. HRMS (ESI) *m/z* $[M+H]^+$ calcd For $C_{16}H_{24}N_3S$, 290.1691; found, 290.1694.

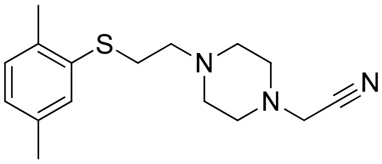

2-(4-(2-((2,5-dimethylphenyl)thio)ethyl)piperazinyl)acetonitrile (**2t**): yield, 74% (42.8 mg); light yellow oil. ^{1}H NMR (400 MHz, $CDCl_3$) δ 7.09 (s, 1H), 7.04 (d, *J* = 7.6 Hz, 1H), 6.89 (d, *J* = 7.6 Hz, 1H), 3.48 (s, 2H), 3.00 (t, *J* = 7.2 Hz, 2H), 2.67 - 2.51 (m, 10H), 2.32 (s, 3H), 2.29 (s, 3H). ^{13}C NMR (101 MHz, $CDCl_3$) δ 136.0, 135.1, 134.6, 130.0, 128.7, 126.6, 114.8, 57.2, 52.5, 51.7, 45.9, 30.2, 21.1, 20.0. HRMS (ESI) *m/z* $[M+H]^+$ calcd For $C_{16}H_{24}N_3S$, 290.1691; found, 290.1694.

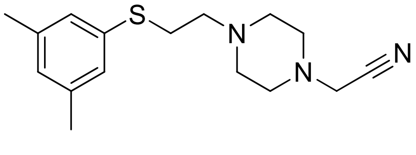

2-(4-(2-((3,5-dimethylphenyl)thio)ethyl)piperazinyl)acetonitrile (**2u**): yield, 78% (45.2 mg); light yellow iol. ^{1}H NMR (400 MHz, $CDCl_3$) δ 6.94 (s, 2H), 6.78 (s, 1H), 3.47 (s, 2H), 3.00 (t, *J* = 7.6 Hz, 2H), 2.65 - 2.46 (m, 10H), 2.26 (s, 6H). ^{13}C NMR (101 MHz, $CDCl_3$) δ 138.5, 135.7, 127.9, 126.7, 114.8, 57.5, 52.5, 51.7, 45.9, 30.7, 21.3. HRMS (ESI) *m/z* $[M+H]^+$ calcd For $C_{16}H_{24}N_3S$, 290.1691; found, 290.1694.

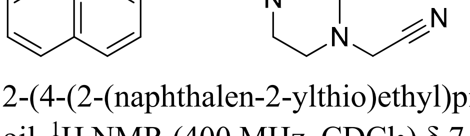

2-(4-(2-(naphthalen-2-ylthio)ethyl)piperazinyl)acetonitrile (**2v**): yield, 57%, (35.5 mg), light yellow oil. ^{1}H NMR (400 MHz, $CDCl_3$) δ 7.79 - 7.70 (m, 4H), 7.49 - 7.39 (m, 3H), 3.46 (s, 2H), 3.13 (t, *J* =

7.6 Hz, 2H), 2.71 - 2.48 (m, 10H). ^{13}C NMR (101 MHz, CDCl$_3$) δ 133.8, 131.7, 128.5, 127.8, 127.3, 127.1, 126.7, 125.7, 114.8, 57.3, 52.5, 51.7, 45.9, 30.7. HRMS (ESI) *m/z* $[M+H]^+$ calcd For $C_{18}H_{22}N_3S$, 312.1534; found, 312.1538.

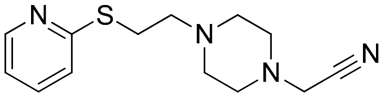

2-(4-(2-(pyridin-2-ylthio)ethyl)piperazinyl)acetonitrile (**2w**): yield, 84% (44.1 mg); light yellow solid. M.p. 97 - 98 °C. ^{1}H NMR (400 MHz, CDCl$_3$) δ 8.40 (d, *J* = 4.0 Hz, 1H), 7.46 (t, *J* = 7.5 Hz, 1H), 7.17 (d, *J* = 8.0 Hz, 1H), 6.96 (t, *J* = 5.6 Hz, 1H), 3.50 (s, 2H), 3.31 (t, *J* = 7.3 Hz, 2H), 2.74 - 2.66 (m, 2H), 2.61 (br s, 8H). ^{13}C NMR (101 MHz, CDCl$_3$) δ 158.6, 149.4, 135.9, 122.2, 119.4, 114.9, 57.5, 52.4, 51.6, 45.8, 26.9. HRMS (ESI) *m/z* $[M+H]^+$ calcd For $C_{13}H_{19}N_4S$, 263.1331; found, 263.1334.

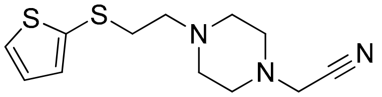

2-(4-(2-(thiophen-2-ylthio)ethyl)piperazinyl)acetonitrile (**2x**): yield, 76% (40.6 mg); light yellow oil. ^{1}H NMR (400 MHz, CDCl$_3$) δ 7.32 (d, *J* = 5.3 Hz, 1H), 7.11 (d, *J* = 3.5 Hz, 1H), 6.95 (dd, *J* = 5.3, 3.6 Hz, 1H), 3.48 (s, 2H), 2.89 (t, *J* = 7.2 Hz, 2H), 2.64 - 2.45 (m, 10H). ^{13}C NMR (101 MHz, CDCl$_3$) δ 134.2, 133.7, 129.3, 127.6, 114.8, 57.5, 52.5, 51.7, 45.9, 35.8. HRMS (ESI) *m/z* $[M+H]^+$ calcd For $C_{12}H_{18}N_3S_2$, 268.0942; found, 268.0946.

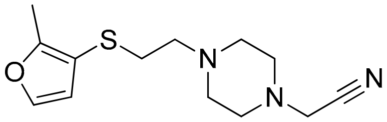

2-(4-(2-((2-methylfuran-3-yl)thio)ethyl)piperazinyl)acetonitrile (**2y**): yield, 45% (23.9 mg); light yellow oil. ^{1}H NMR (400 MHz, CDCl$_3$) δ 7.21 (d, *J* = 2.0 Hz, 1H), 6.27 (d, *J* = 1.8 Hz, 1H), 3.43 (s, 2H), 2.66 (t, *J* = 7.2 Hz, 2H), 2.57 - 2.41 (m, 10H), 2.27 (s, 3H). ^{13}C NMR (101 MHz, CDCl$_3$) δ 154.9, 140.6, 115.0, 114.7, 110.0, 57.9, 52.5, 51.7, 45.9, 32.8, 11.9. HRMS (ESI) *m/z* $[M+H]^+$ calcd For $C_{13}H_{20}N_3OS$, 266.1327; found, 266.1331.

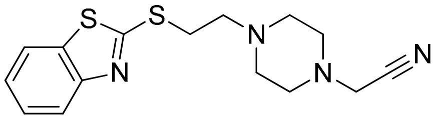

2-(4-(2-(benzo[*d*]thiazol-2-ylthio)ethyl)piperazinyl)acetonitrile (**2z**): yield, 60% (38.2 mg); light yellow solid. M.p. 77 - 78 °C. ^{1}H NMR (400 MHz, CDCl$_3$) δ 7.77 (d, *J* = 8.0 Hz, 1H), 7.67 (d, *J* = 7.9 Hz, 1H), 7.32 (t, *J* = 7.7 Hz, 1H), 7.20 (t, *J* = 7.6 Hz, 1H), 3.44 (t, *J* = 7.1 Hz, 2H), 3.42 (s, 2H), 2.74 (t, *J* = 7.2 Hz, 2H), 2.54 (br s, 8H). **^{13}C** NMR (101 MHz, CDCl$_3$) δ 167.0, 153.2, 135.2, 126.1, 124.3, 121.4, 121.0, 114.8, 56.7, 52.4, 51.7, 45.9, 30.8. HRMS (ESI) *m/z* $[M+H]^+$ calcd For $C_{15}H_{19}N_4S_2$, 319.1051; found, 319.1055.

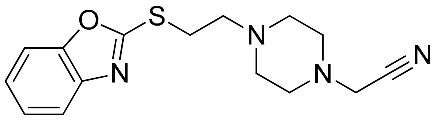

2-(4-(2-(benzo[*d*]oxazol-2-ylthio)ethyl)piperazinyl)acetonitrile (**2aa**): yield, 68% (41.1 mg); light yellow solid. M.p. 57 - 58 °C. ^{1}H NMR (400 MHz, CDCl$_3$) δ 7.50 (d, *J* = 7.3 Hz, 1H), 7.35 (d, *J* = 7.5 Hz, 1H), 7.17 (tt, *J* = 7.5, 6.4 Hz, 2H), 3.41 (s, 2H), 3.38 (t, *J* = 6.9 Hz, 2H), 2.74 (t, *J* = 6.9 Hz, 2H), 2.54 (br s, 8H). ^{13}C NMR (101 MHz, CDCl$_3$) δ 165.2, 151.8, 141.9, 124.3, 123.9, 118.3, 114.8, 109.9, 56.5, 52.3, 51.7, 45.9, 30.0. HRMS (ESI) *m/z* [M+H]$^+$ calcd For $C_{15}H_{19}N_4OS$, 303.1280; found, 303.1283.

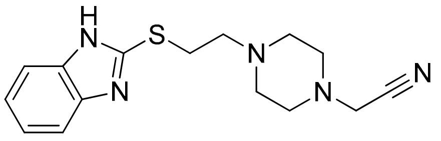

2-(4-(2-((1*H*-benzo[*d*]imidazol-2-yl)thio)ethyl)piperazinyl)acetonitrile (**2ab**): yield, 71% (42.8 mg); light yellow solid. M.p. 139 - 140 °C. ^{1}H NMR (400 MHz, CDCl$_3$) δ 10.66 (s, 1H), 7.54 (dd, *J* = 6.0, 3.2 Hz, 2H), 7.10 (dd, *J* = 6.0, 3.2 Hz, 2H), 3.48 (s, 2H), 3.14 (t, *J* = 5.2 Hz, 2H), 2.81 (t, *J* = 5.6 Hz, 2H), 2.63 (br s, 8H). ^{13}C NMR (101 MHz, CDCl$_3$) δ 151.0, 139.7, 122.1, 114.7, 114.3, 60.0, 52.8, 51.4, 45.8, 29.7. HRMS (ESI) *m/z* [M+H]$^+$ calcd For $C_{15}H_{20}N_5S$, 302.1439; found, 302.1443.

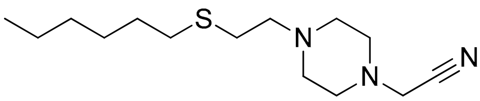

2-(4-(2-(hexylthio)ethyl)piperazinyl)acetonitrile (**2ac**): yield, 51%, (27.5 mg), light yellow oil. ^{1}H NMR (400 MHz, CDCl$_3$) δ 3.45 (s, 2H), 2.60 - 2.43 (m, 14H), 1.55 - 1.47 (m, 2H), 1.34 - 1.18 (m, 6H), 0.82 (t, *J* = 6.8 Hz, 3H). ^{13}C NMR (101 MHz, CDCl$_3$) δ 114.8, 58.2, 52.5, 51.7, 45.8, 32.4, 31.4, 29.7, 29.1, 28.5, 22.5, 14.1. HRMS (ESI) *m/z* [M+H]$^+$ calcd For $C_{14}H_{28}N_3S$, 270.2004; found, 270.2007.

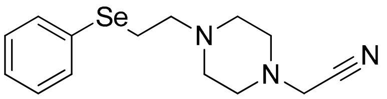

2-(4-(2-(phenylselanyl)ethyl)piperazinl)acetonitrile (**2ad**): yield, 80%, (49.3 mg), light yellow oil. ^{1}H NMR (400 MHz, CDCl$_3$) δ 7.49 (d, *J* = 7.8 Hz, 2H), 7.29 - 7.20 (m, 3H), 3.48 (s, 2H), 3.02 (t, *J* = 7.2 Hz, 2H), 2.70 (t, *J* = 8.0 Hz, 2H), 2.63 - 2.49 (m, 8H). ^{13}C NMR (101 MHz, CDCl$_3$) δ 132.4, 130.3, 129.1, 126.9, 114.8, 58.1, 52.3, 51.7, 45.9, 24.8. HRMS (ESI) *m/z* [M+H]$^+$ calcd For $C_{14}H_{20}N_3Se$, 310.0817; found, 310.0819.

4. X-ray Structure Determinations

Crystals were obtained from the following solvents: CH_2Cl_2/hexane (**2b**, **2ab, 2w, 2z, 2ab**), CH_2Cl_2/MeOH (**3b**, **4b**), CH_2Cl_2/THF (**5a**). Diffraction data were collected on an Oxford Diffraction Supernova dual diffractometer equipped with an Oxford Cryostream 700 low-temperature apparatus. Cu K\a radiation source (λ = 1.54184 Å) was used for the data collection. Single crystals were coated with Paratone-N oil and mounted on a Nylon loop for diffraction. The data reduction and cell refinement were processed using CrysAlisPro software.[8] Structures were solved by direct methods using the SHELXTL program packages.[9] All non-hydrogen atoms were refined anisotropically and hydrogen atoms were added geometrically. Crystal data and refinement details were shown in Table S2.

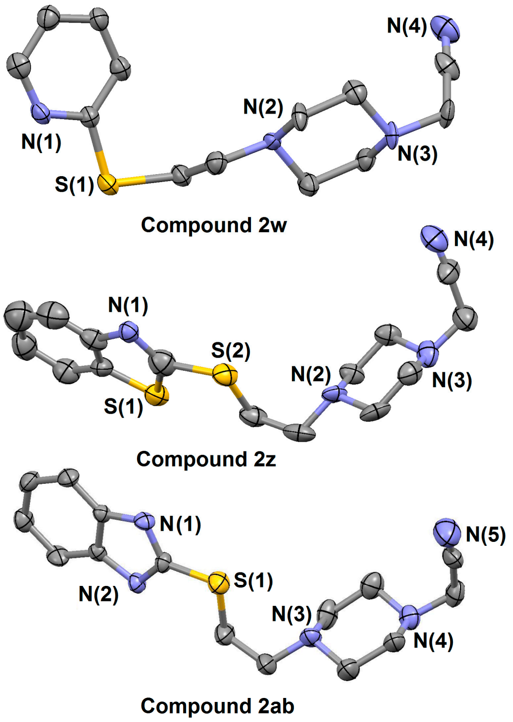

Figure S1. Crystal structures of compounds **2w** (7.2330(2) 7.6600(3) 15.5330(5) 79.930 83.990 88.860, V = 842.70(5), space group P-1, fast data), **2z** (15.4151(4) 5.7350(2) 18.0257(7) 90 90 90, V = 1593.57(10), space group $Pna2_1$, fast data) and **2ab** (11.6990(2) 10.2567(8) 26.3120(2) 90 90 90, V = 3157.30(6), space group Pbca, fast data).

5. Crystallographic data of compounds

Table S2. Crystallographic data[a] for compounds **2b**, **2ab**, **3b**, **4b**, and **5a** (fast data).

	**2b**	**2ab**	**3b**	**4b**	**5a·0.5 THF**
formula	$C_{15}H_{21}N_3O_1S_1$	$C_{15}H_{19}N_5S_1$	$C_{14}H_{20}N_4O_3S_1$	$C_{15}H_{21}N_5O_1S_1$	$C_{29}H_{41}N_5O_{1.5}S_1$
M	291.41	301.41	324.40	319.43	515.73
crystal system	monoclinic	orthorhombic	orthorhombic	orthorhombic	monoclinic
space group	$P2_1$	Pbca	$Pca2_1$	Pbca	I2/a
a, Å	7.5004(4)	11.6534(3)	8.1136(2)	7.6388(6)	30.270(3)
b, Å	5.4857(3)	10.2429(2)	19.3247(5)	9.9296(7)	10.5566(7)
c, Å	18.5116(9)	26.2434(5)	9.9989(2)	41.256(4)	35.497(4)
α, deg	90.000	90.000	90.000	90.000	90.000
β, deg	92.709	90.000	90.000	90.000	99.867
γ, deg	90.000	90.000	90.000	90.000	90.000
V, $Å^3$	760.81(7)	3132.53(12)	1567.76(6)	3129.3(4)	11175.0(18)
Z	2	8	4	8	16
μ, mm^{-1}	1.881	1.838	2.002	1.918	1.278
independent data	2337	2925	2236	5300	4999
refined parameters	182	190	199	187	666
$R_1{}^b$, $wR_2{}^c(I>2\sigma(I))$	0.0519, 0.1276	0.0976, 0.2914	0.0680, 0.1844	0.1131, 0.2796	0.0947, 0.1991
R_1,wR_2 (all data)	0.0587, 0.1314	0.1176, 0.3037	0.0720, 0.1921	0.1305, 0.2866	0.2026, 0.2517

[a]T = 150(2) K, Cu Kα radiation (λ = 1.54178 Å). [b]$R_1 = \Sigma||F_o| - |F_c||/\Sigma|F_o|$. [c]$wR_2 = \{\Sigma[w(F_o^2 - F_c^2)^2/(F_o^2)^2]\}^{1/2}$.

6. References

1. McCarthy, P. A.; Hamanaka, E. S.; Marzetta, C. A.; Bamberger, M. J.; Gaynor, B. J.; Chang, G.; Kelly, S. E.; Inskeep, P. B.; Mayne, J. T.; Beyer, T. A.; Walker, F. J.; Goldberg, D. I.; Savoy, Y. E.; Davis, K. M.; Diaz, C. L.; Freeman, A. M.; Johnson, D. A.; LaCour, T. G.; Long, C. A.; Maloney, M. E.; Martingano, R. J.; Pettini, J. L.; Sand, T. M.; Wint, L. T. *J. Med. Chem.* **1994**, *37*, 1252-1255.
2. Majdecki, M.; Niedbala, P.; Jurczak, J. *Org. Lett.* **2019**, *21*, 8085-8090.
3. Ali, M. H.; McDermott, M. *Tetrahedron Lett.* **2002**, *43*, 6271-6273.
4. (a) Laali, K. K.; Jamalian, A.; Zhao, C. Q. *Tetrahedron Lett.* **2014**, *55*, 6643-6646. (b) Yang, Z. J.; Fan, M. J.; Mu, R. Z.; Liu, W. M.; Liang, Y. M. *Tetrahedron Lett.* **2005**, *61*, 9140-9146. (c) Lauw, Y.; Rüther, T.; Horne, M. D.; Wallwork, K. S.; Skelton, B. W.; Madsen, I. C.; Rodopoulos, T. *Cryst. Growth Des.* **2012**, *12*, 2803-2813.
5. Xu, J. M.; Shi, S. Z.; Liu, G.; Xie, X. P.; Li, J.; Bolinger, A. A.; Chen, H. Y.; Zhang, W. B.; Shiand, P. Y.; Liu, H. *Eur. J. Med.Chem.* **2023**, *246*, 114998.
6. Srinivas, M.; Hudwekar, A. D.; Venkateswarlu, V.; Reddy, G. L.; Kumar, K. A. A.; Vishwakarma, R. A.; Sawant, S. D. *Tetrahedron Lett.* **2015**, *56*, 4775-4779.
7. Shibuya, K.; Miura, T.; Ohgiya, T.; Omichi, K.; Tsunenari, Y. *Bioorgan. Med. Chem.* **2020**, *28*, 115457.
8. CrysAlisPro, Oxford Diffraction (Poland), **2010**.
9. (a) Sheldrick, G. M. SHELXS-97, Program for the Solution of Crystal Structure. University of Göttingen, Germany **1997**. (b) Sheldrick, G. M. *Acta Crystallogr.*, **2015**, *C71*, 3.

7. ^{1}H NMR and ^{13}C NMR spectra of compounds

^{1}H NMR spectrum (400 MHz, $CDCl_3$) of Bis(4-methoxyphenyl) disulfide (1b)

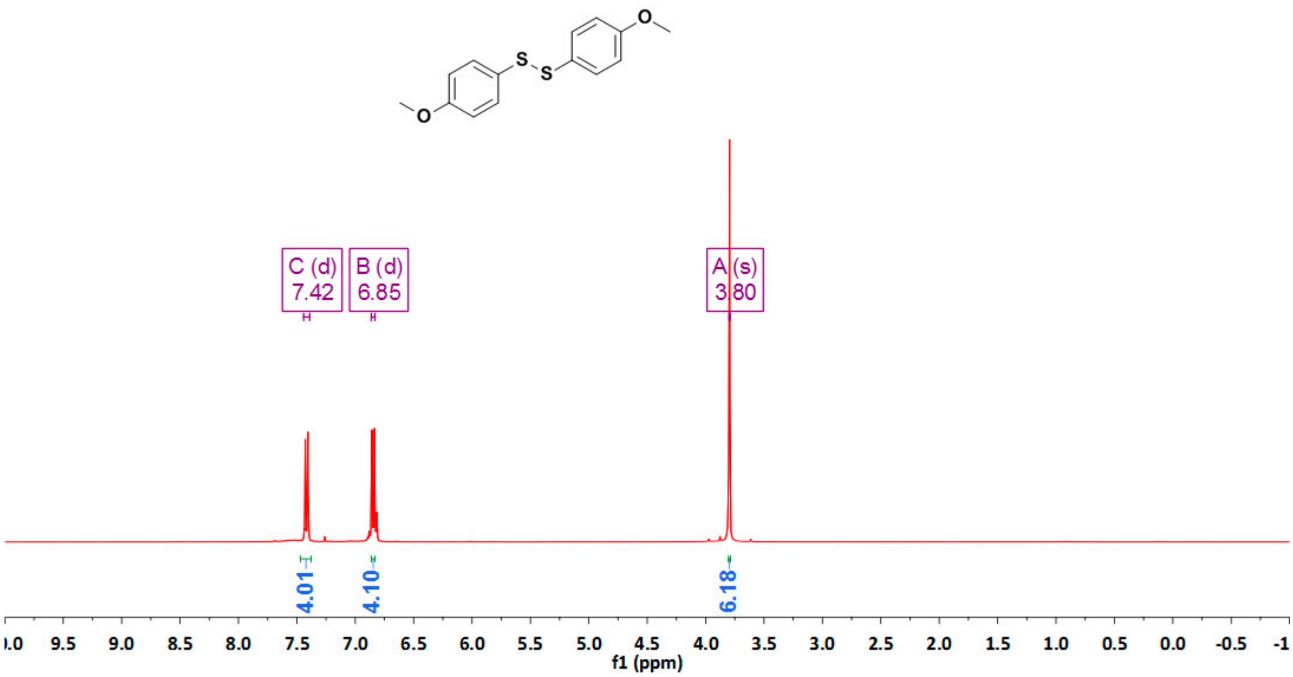

^{1}H NMR spectrum (400 MHz, $CDCl_3$) of Bis(4-*tert*-butylphenyl) disulfide (1c)

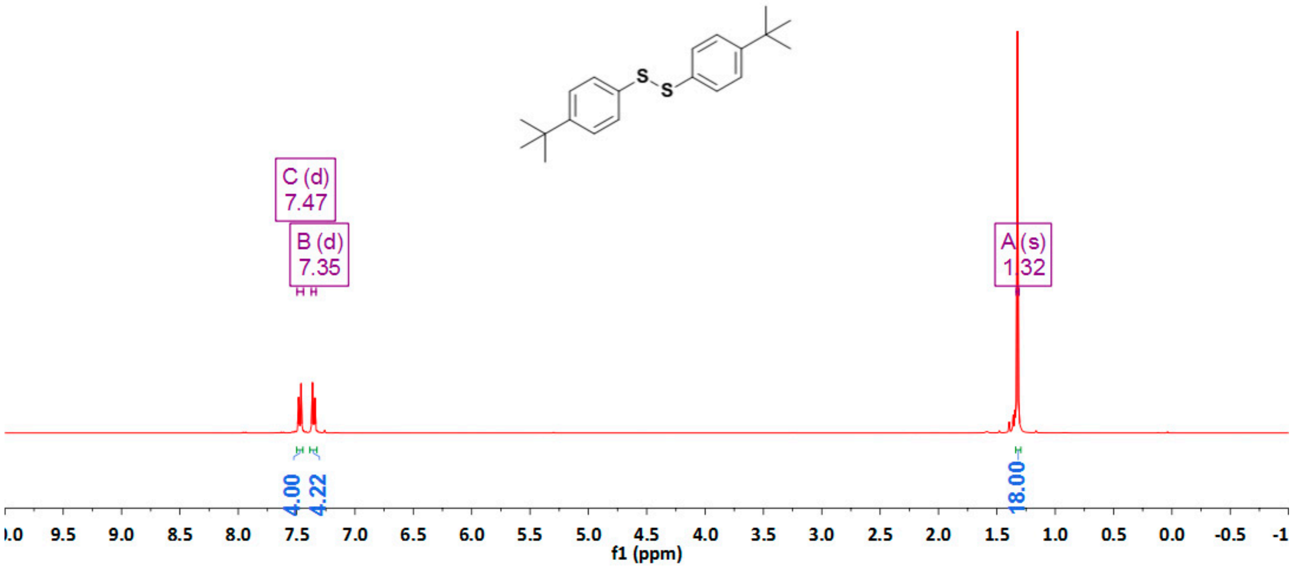

^{1}H NMR spectrum (400 MHz, $CDCl_3$) of Bis(4-methylphenyl) disulfide (1d)

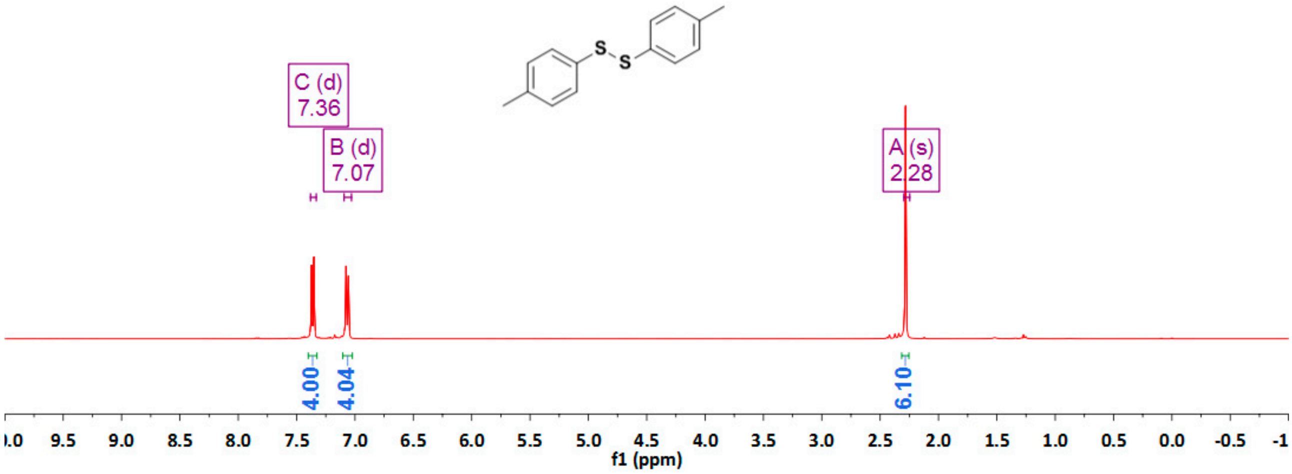

^{1}H NMR spectrum (400 MHz, $CDCl_3$) of Bis(4-bromophenyl) disulfide (1e)

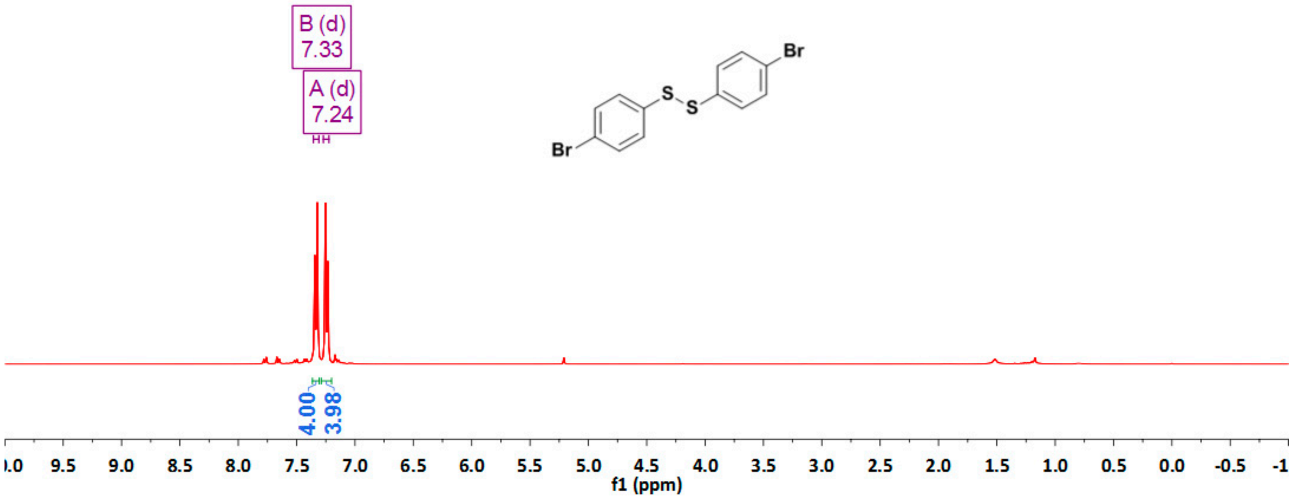

^{1}H NMR spectrum (400 MHz, $CDCl_3$) of Bis(4-chlorophenyl) disulfide (1f)

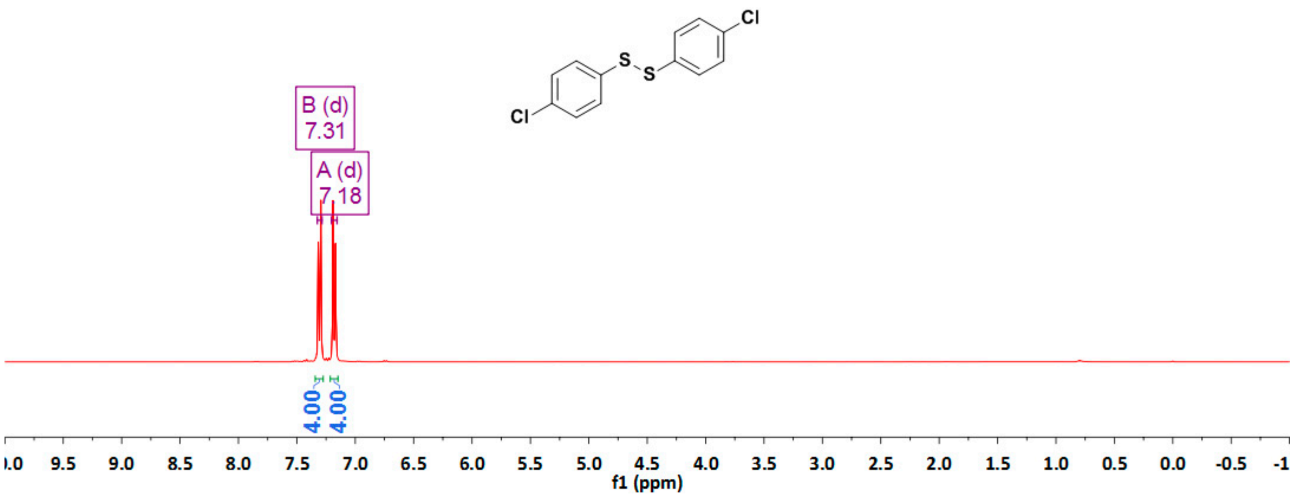

^{1}H NMR spectrum (400 MHz, $CDCl_3$) of Bis(4-fluorophenyl)disulfide (1g)

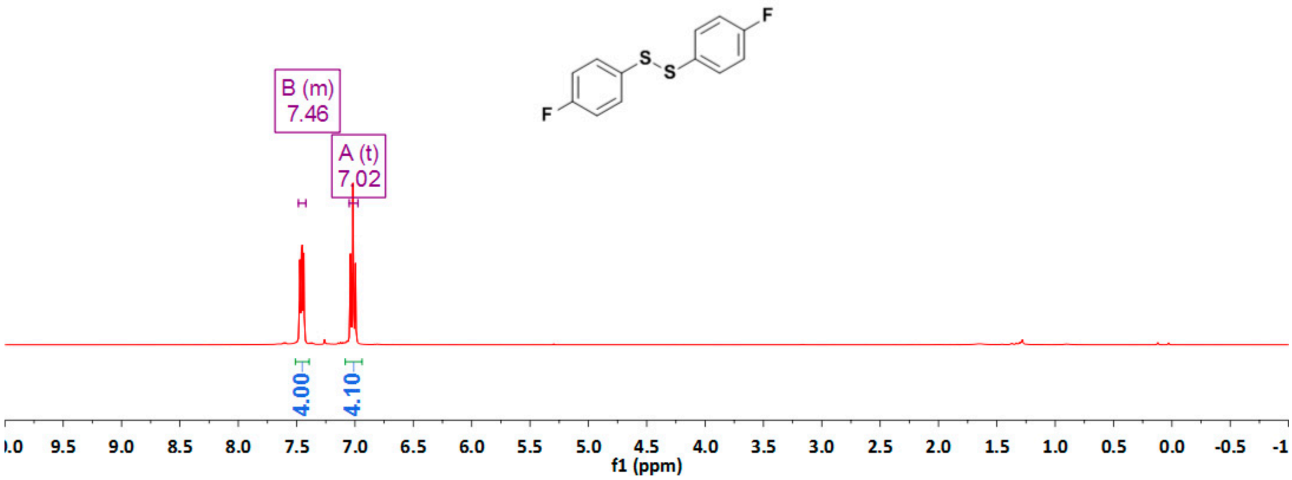

^{1}H NMR spectrum (400 MHz, $CDCl_3$) of Bis(4-(trifluoromethyl)phenyl) disulfide (1h)

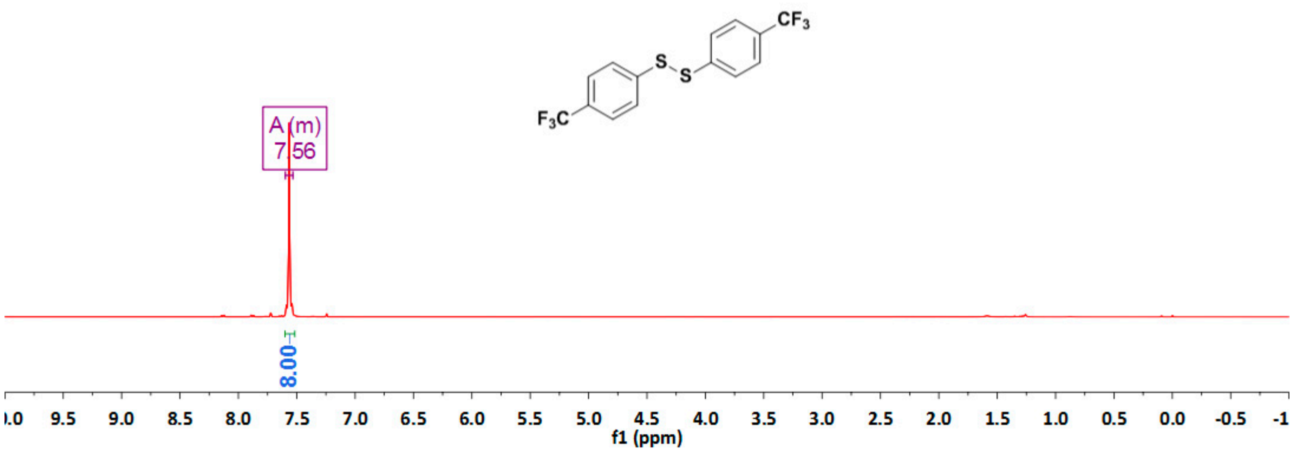

^{1}H NMR spectrum (400 MHz, $CDCl_3$) of Bis(3-methoxyphenyl) disulfide (1i)

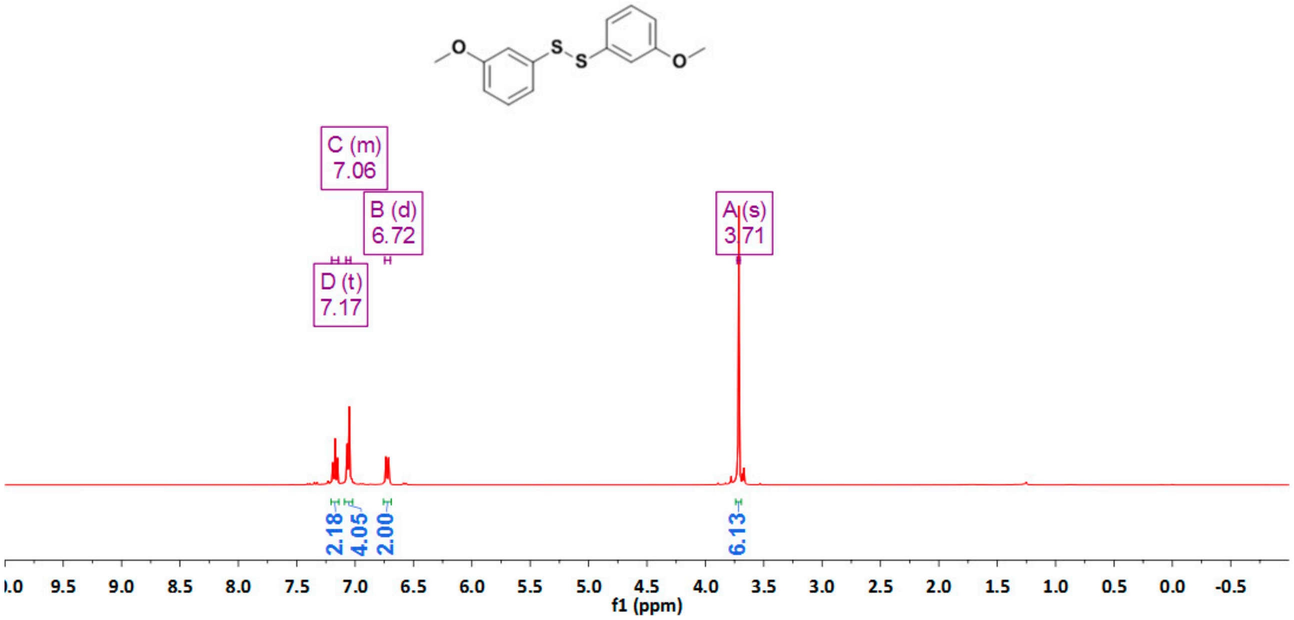

^{1}H NMR spectrum (400 MHz, $CDCl_3$) of Bis(3-methylphenyl) disulfide (1j)

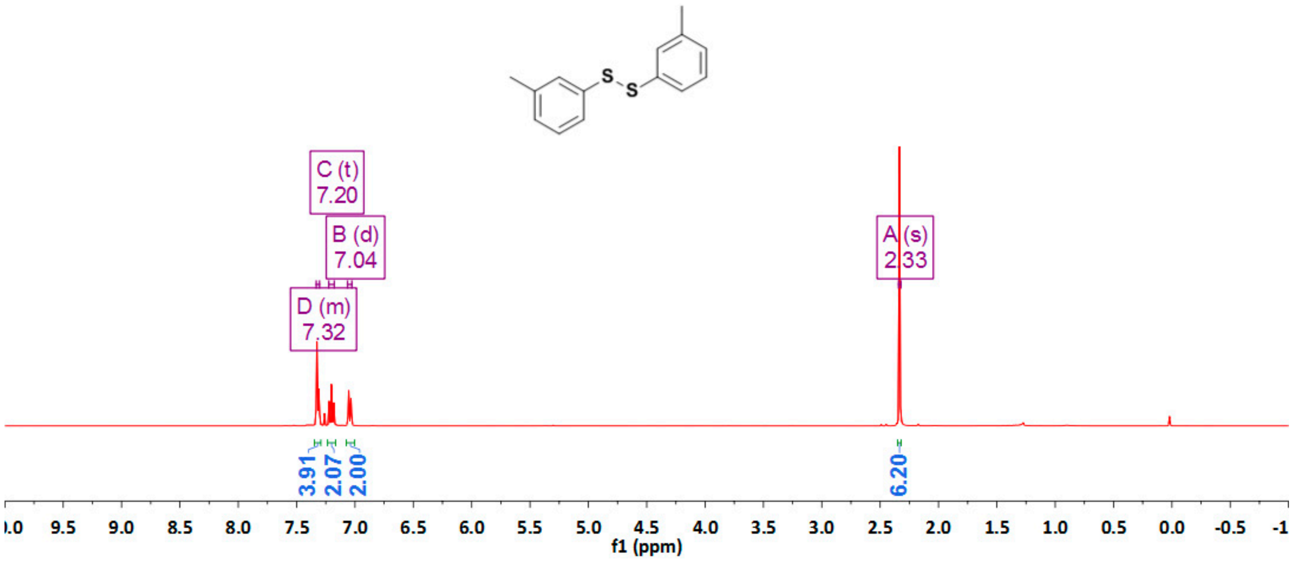

^{1}H NMR spectrum (400 MHz, $CDCl_3$) of Bis(3-bromophenyl)disulfide (1k)

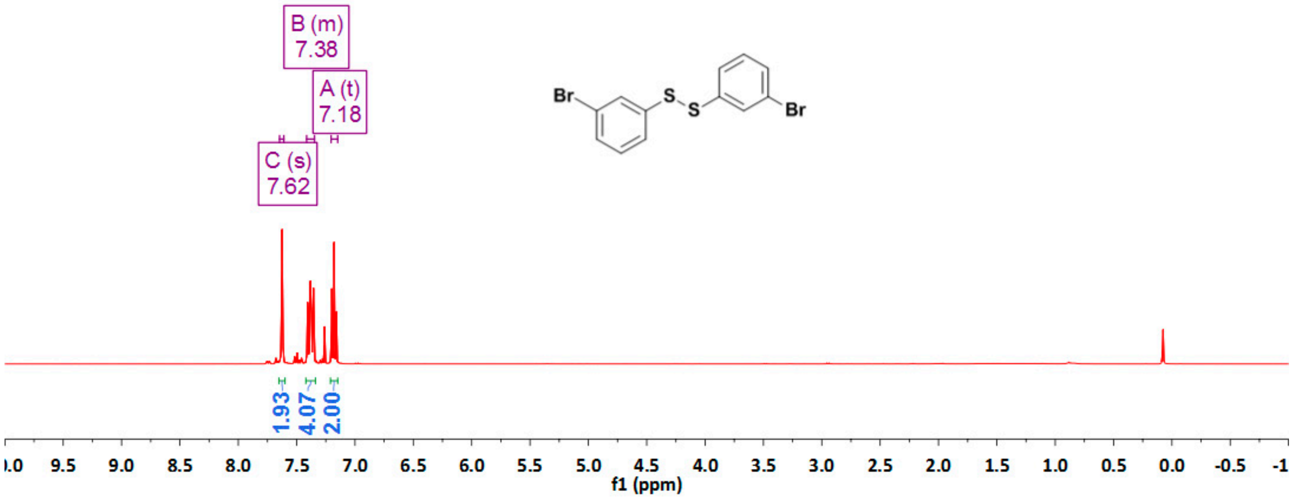

^{1}H NMR spectrum (400 MHz, $CDCl_3$) of Bis(3-chlorophenyl) disulfide (1l)

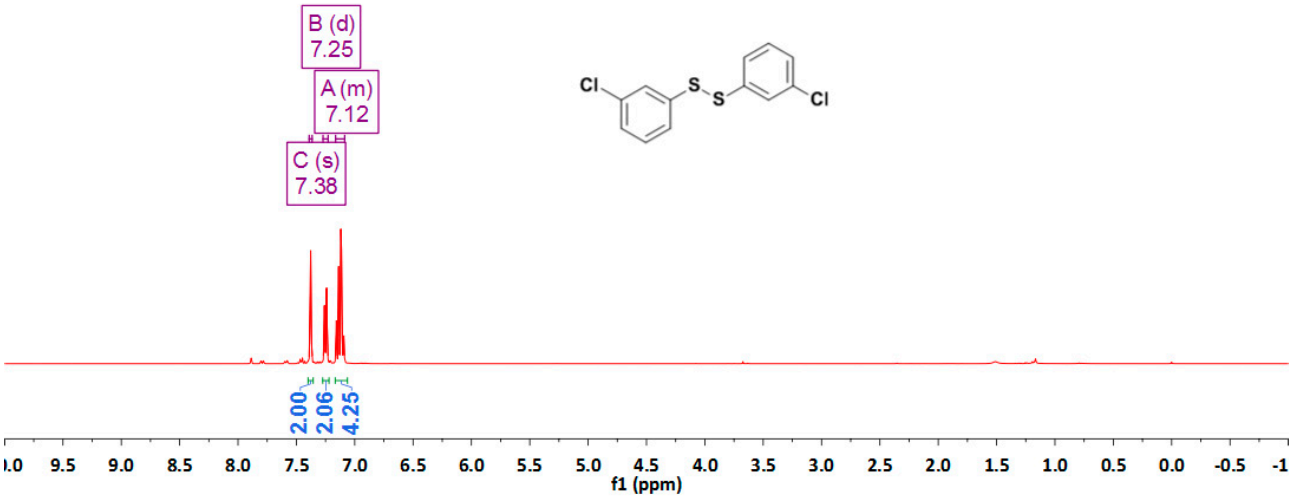

^{1}H NMR spectrum (400 MHz, $CDCl_3$) of Bis(3-fluorophenyl)disulfide (1m)

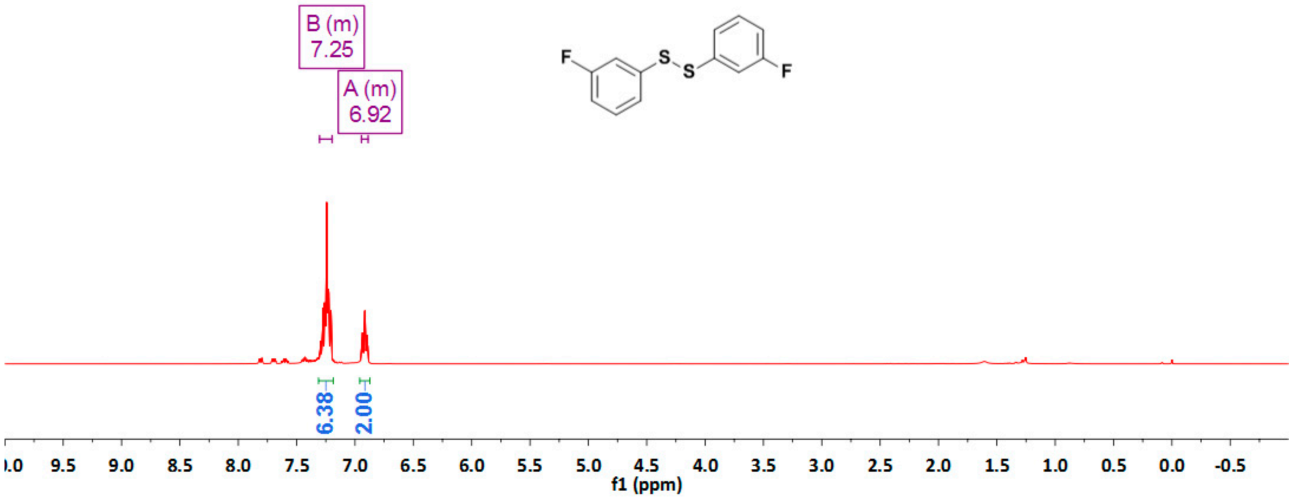

^{1}H NMR spectrum (400 MHz, $CDCl_3$) of Bis(2-methoxyphenyl) disulfide (1n)

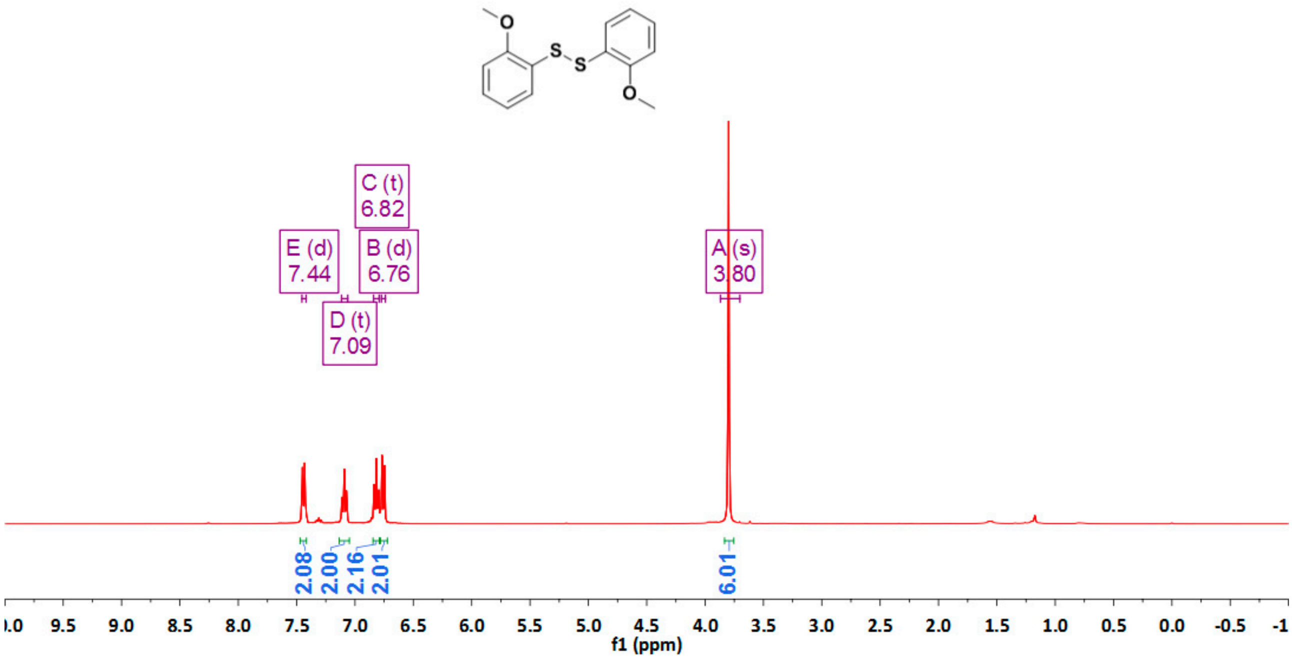

^{1}H NMR spectrum (400 MHz, $CDCl_3$) of Bis(2-methylphenyl) disulfide (1o)

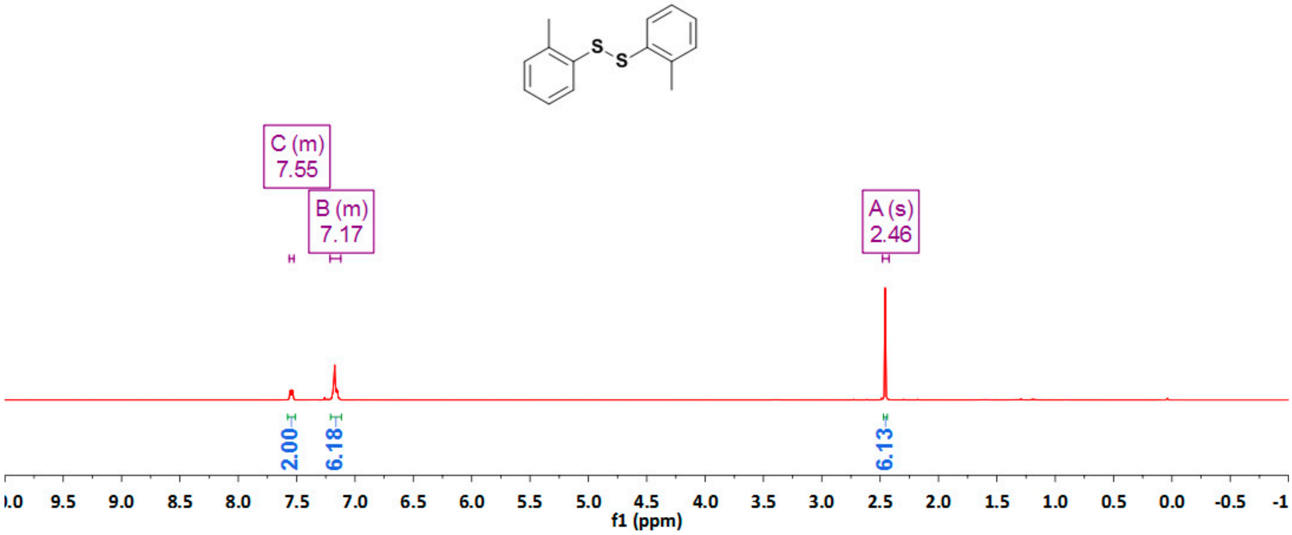

^{1}H NMR spectrum (400 MHz, $CDCl_3$) of Bis(2-bromophenyl)disulfide (1p)

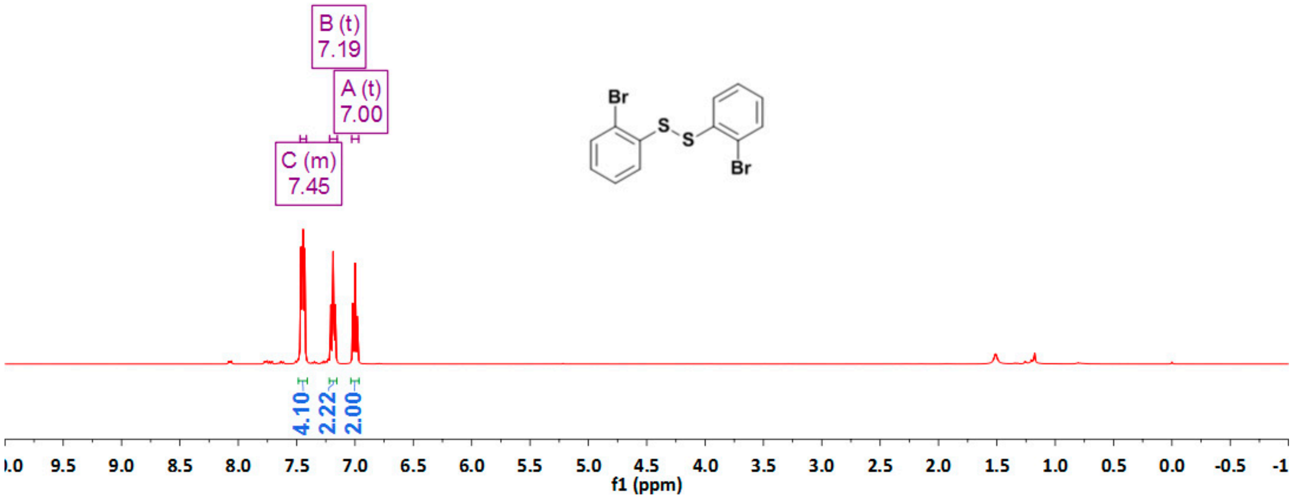

^{1}H NMR spectrum (400 MHz, $CDCl_3$) of Bis(2-chlorophenyl) disulfide (1q)

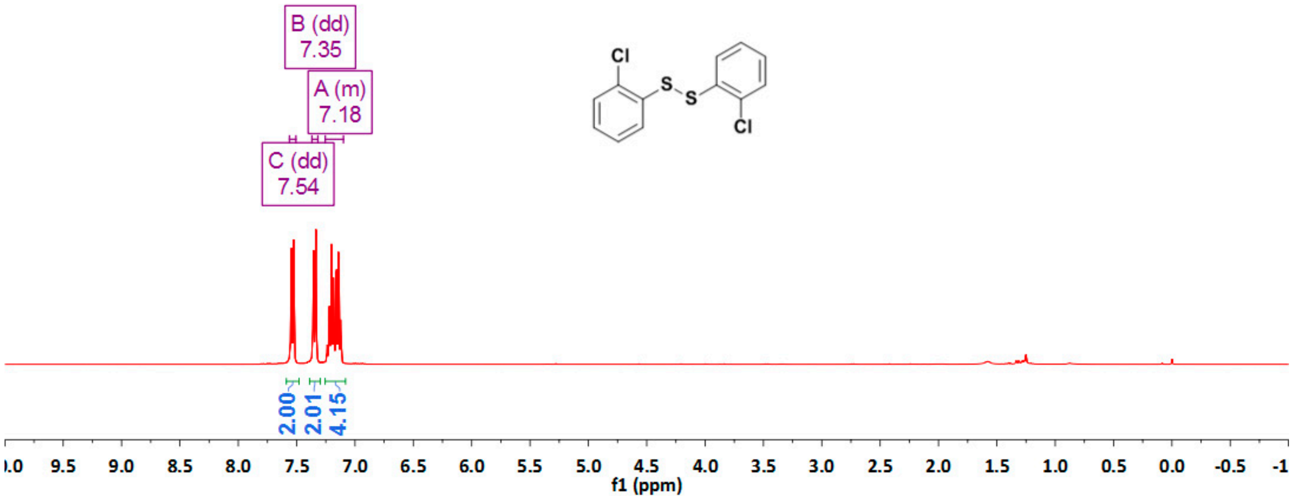

^{1}H NMR spectrum (400 MHz, $CDCl_3$) of Bis(2,4-dimethylphenyl)disulfide (1s)

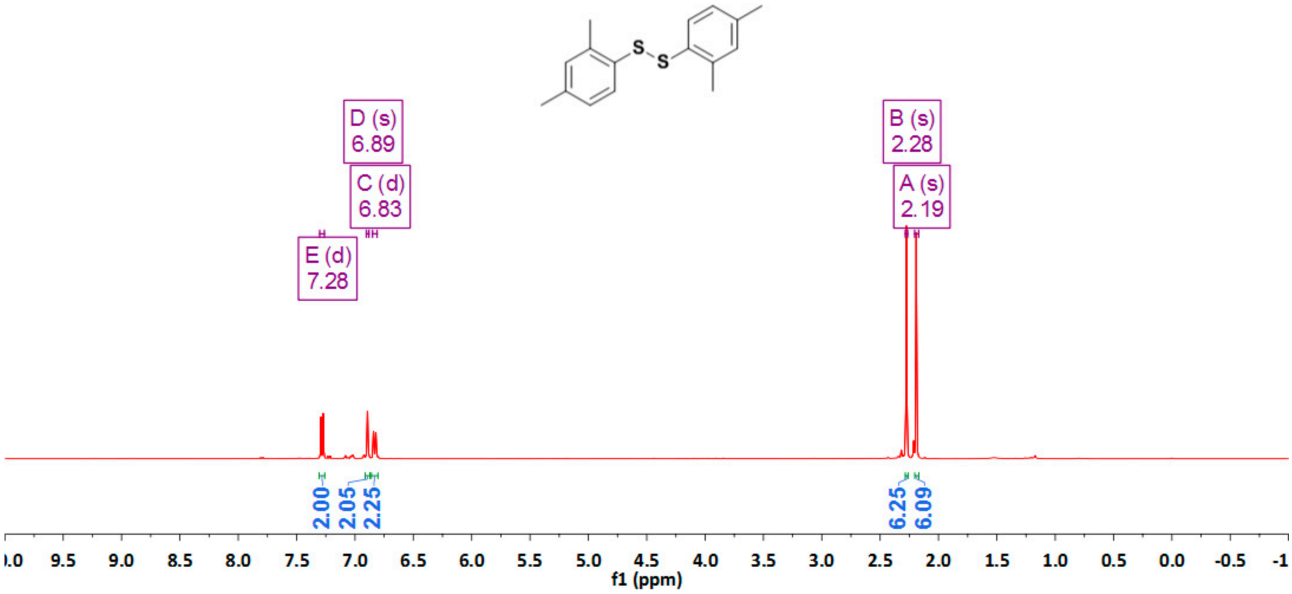

^{1}H NMR spectrum (400 MHz, $CDCl_3$) of Bis(3,5-dimethylphenyl)disulfide (1u)

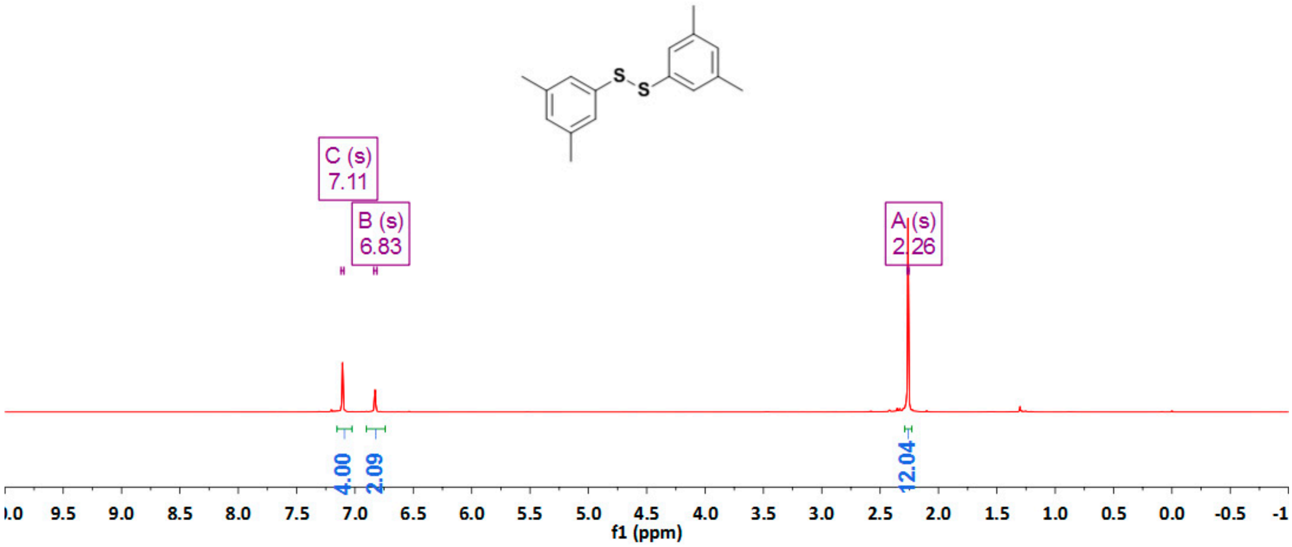

^{1}H NMR spectrum (400 MHz, $CDCl_3$) of Bis(2-naphthyl)disulfide (1v)

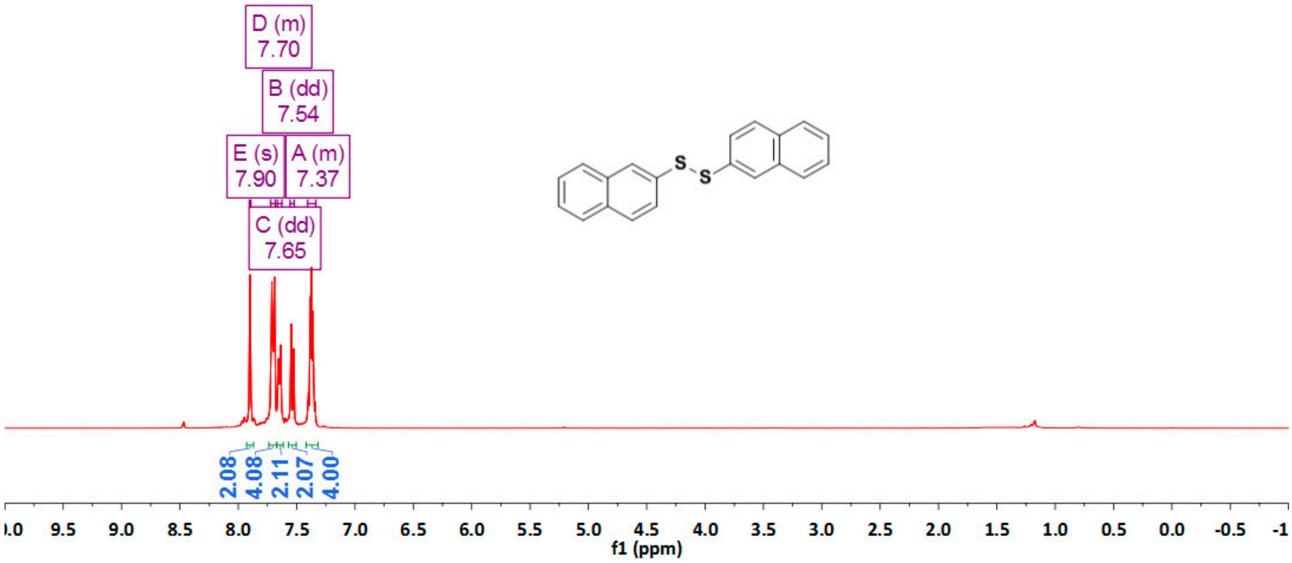

^{1}H NMR spectrum (400 MHz, $CDCl_3$) of dihexyl disulfide (1ac)

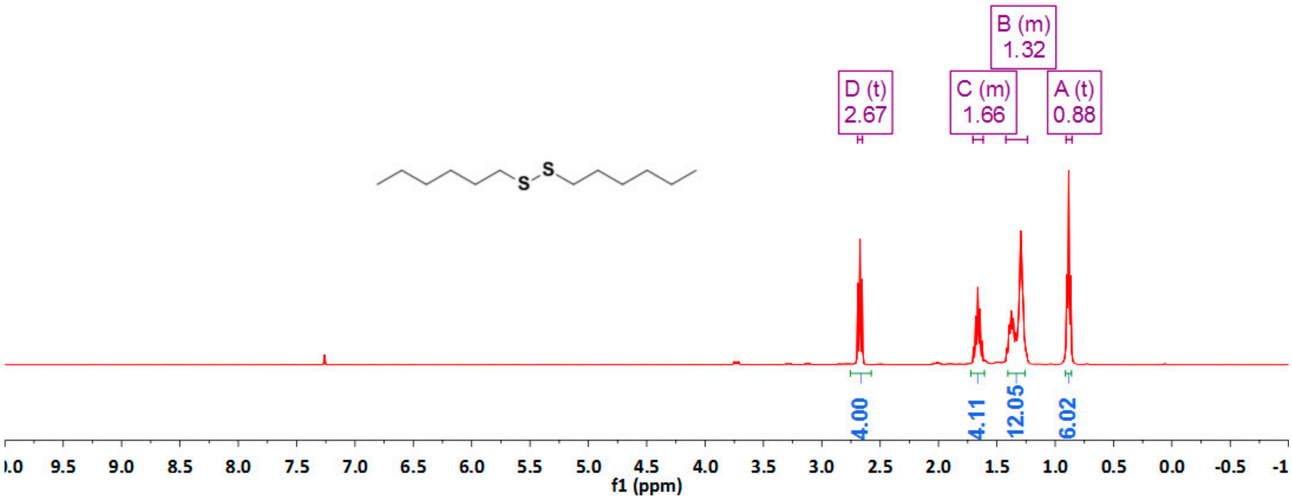

^{1}H NMR spectrum (400 MHz, $CDCl_3$) and ^{13}C NMR spectrum (101 MHz, $CDCl_3$) of 2-(4-(2-(phenylthio)ethyl)piperazinyl)acetonitrile (2a)

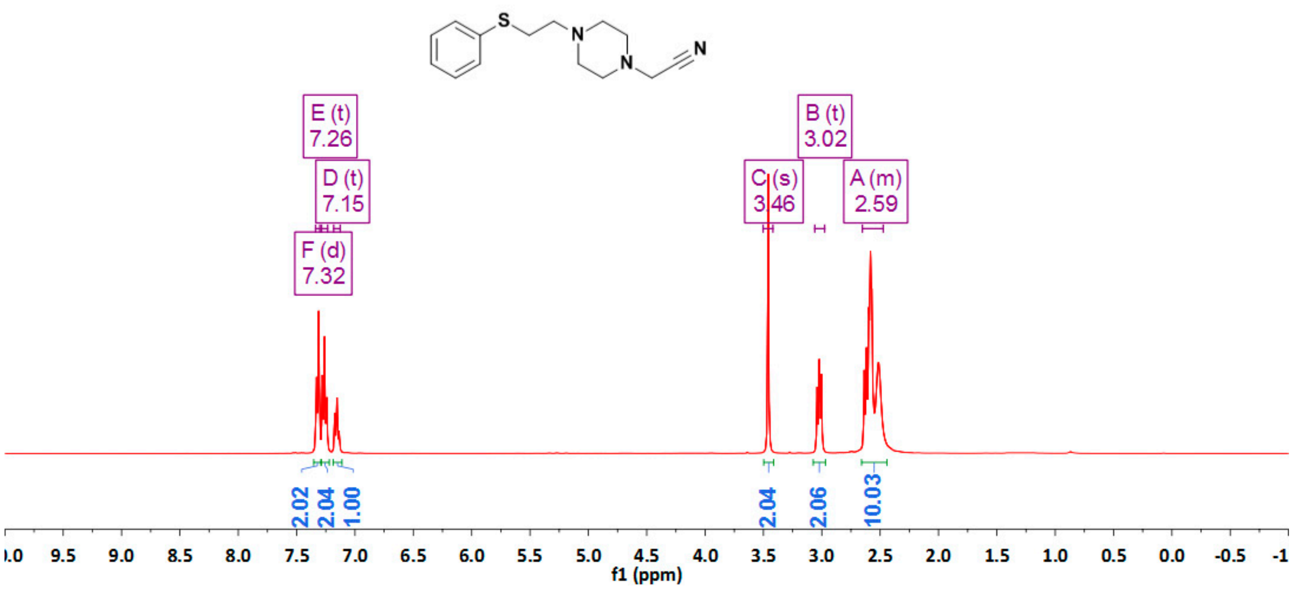

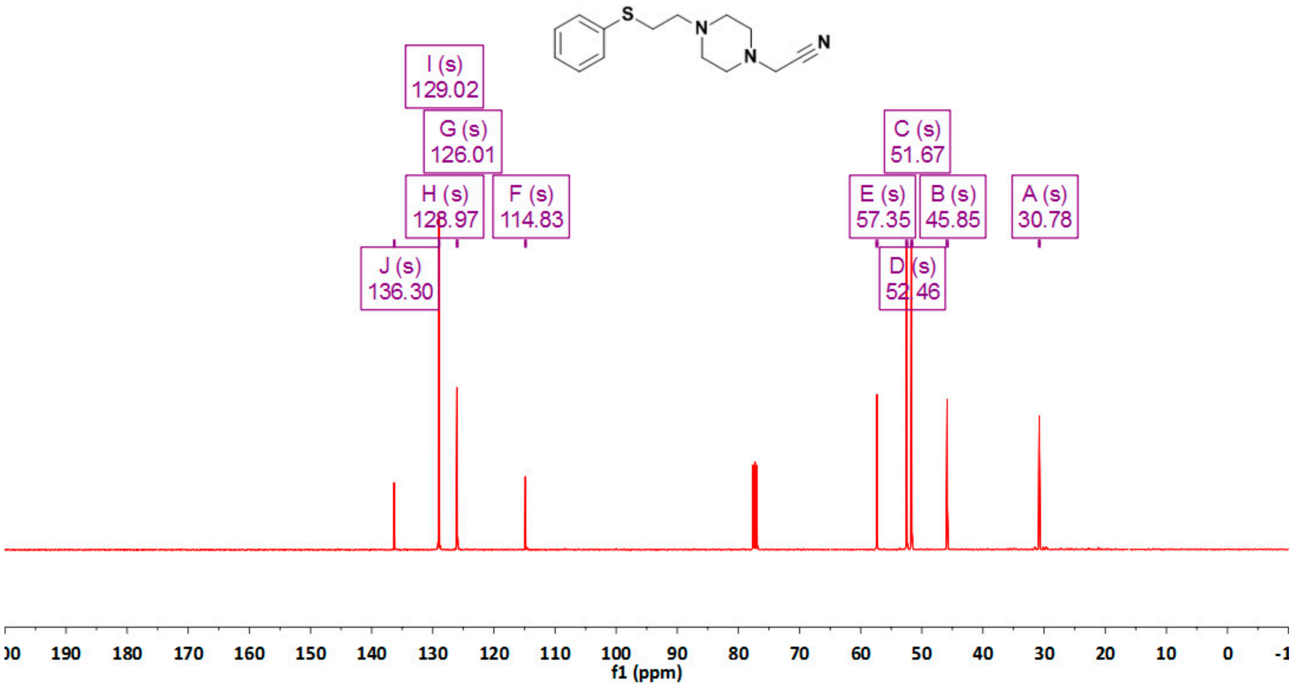

^{1}H NMR spectrum (400 MHz, $CDCl_3$) and ^{13}C NMR spectrum (101 MHz, $CDCl_3$) of 2-(4-(2-((4-methoxyphenyl)thio)ethyl)piperazinyl)acetonitrile (2b)

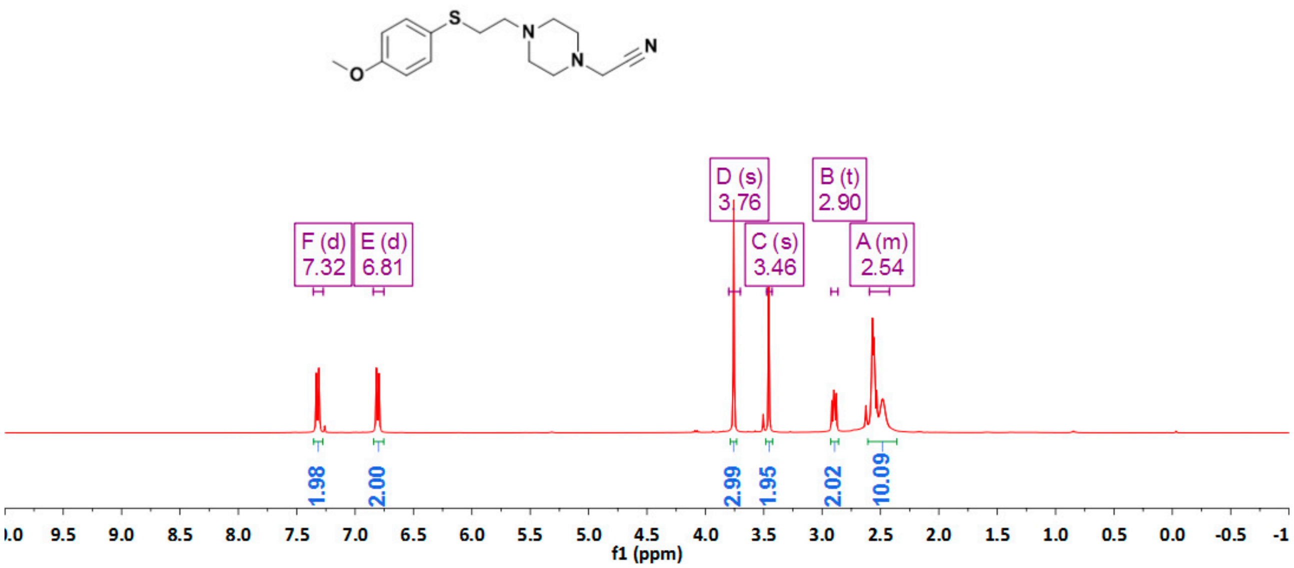

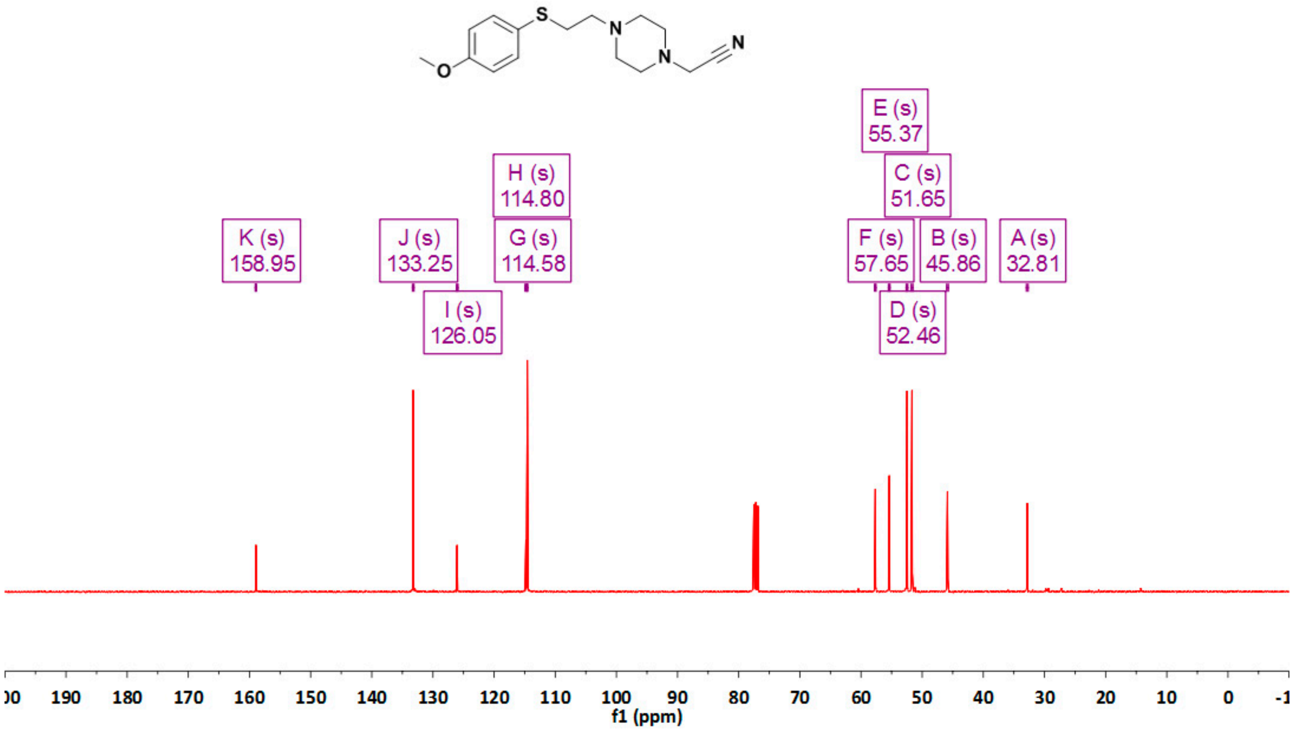

^{1}H NMR spectrum (400 MHz, $CDCl_3$) and ^{13}C NMR spectrum (101 MHz, $CDCl_3$) of 2-(4-(2-((4-(tert-butyl)phenyl)thio)ethyl)piperazinyl)acetonitrile (2c)

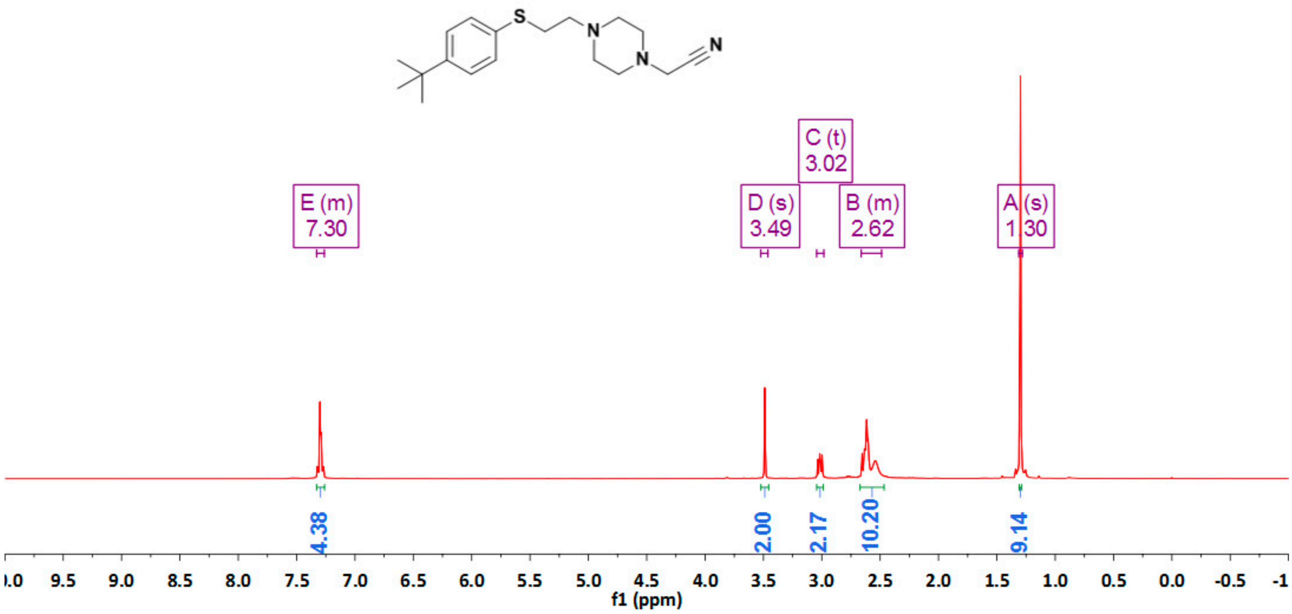

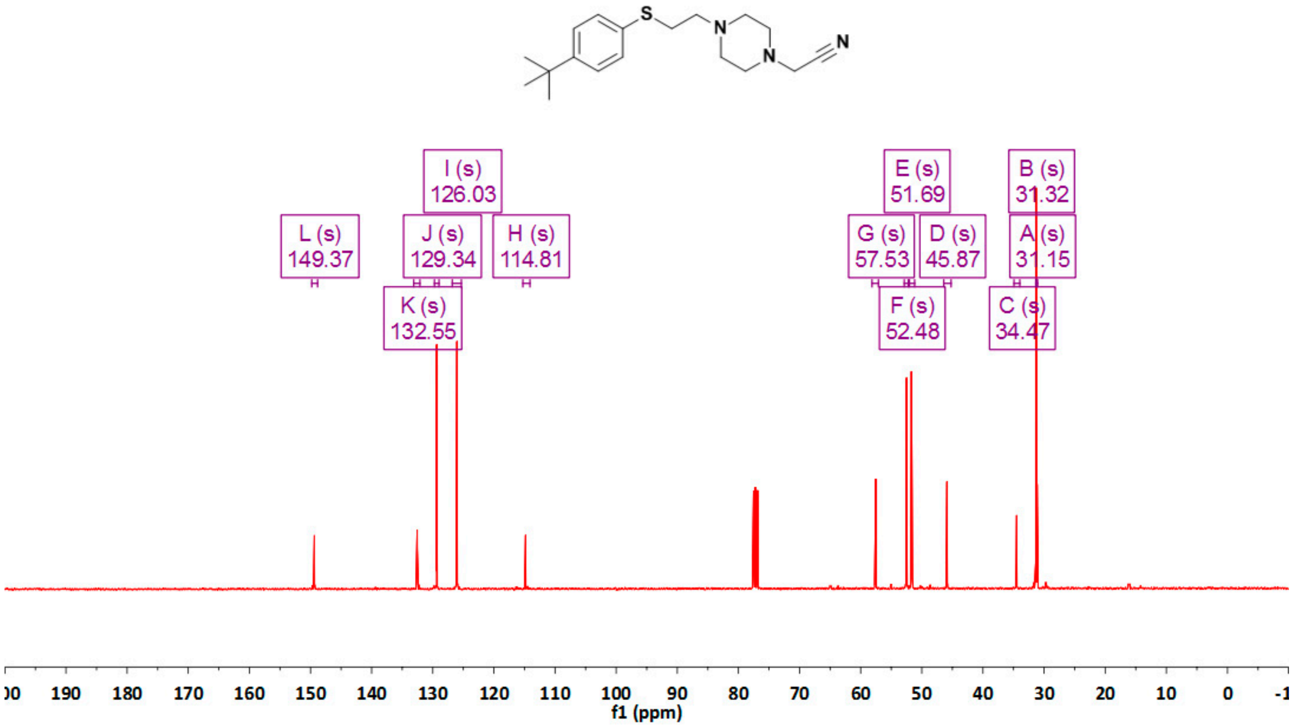

^{1}H NMR spectrum (400 MHz, $CDCl_3$) and ^{13}C NMR spectrum (101 MHz, $CDCl_3$) of 2-(4-(2-(p-tolylthio)ethyl)piperazinyl)acetonitrile (2d)

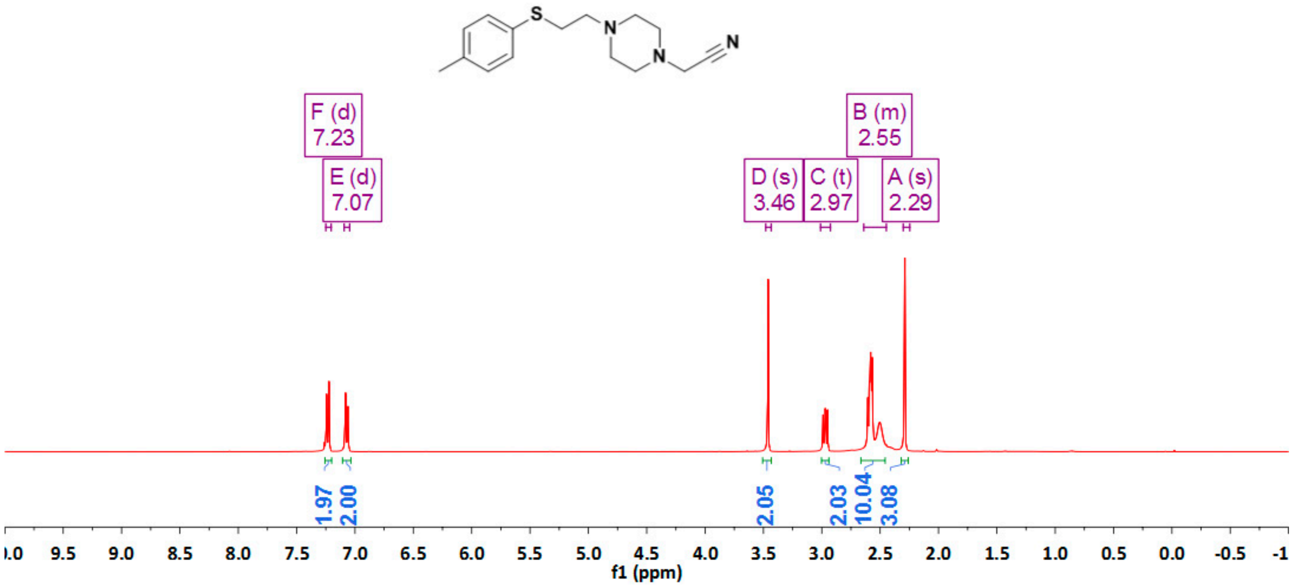

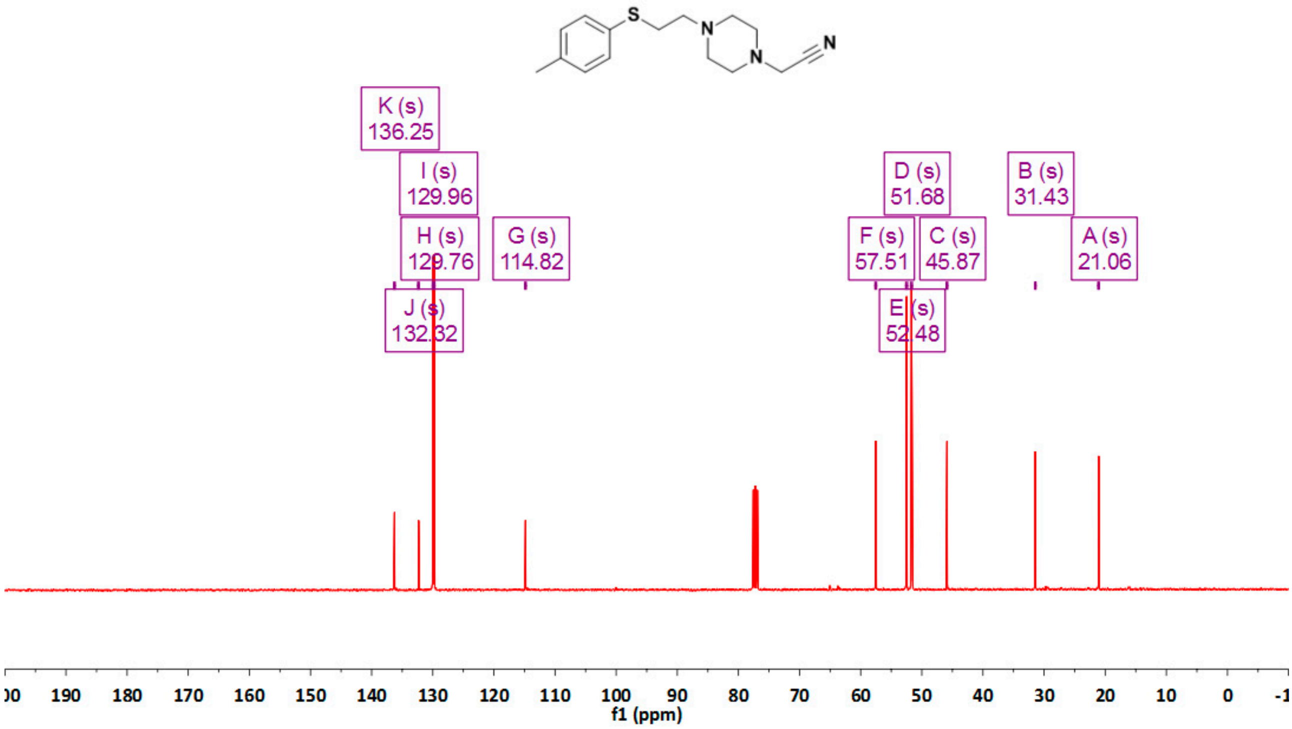

^{1}H NMR spectrum (400 MHz, $CDCl_3$) and ^{13}C NMR spectrum (101 MHz, $CDCl_3$) of 2-(4-(2-((4-bromophenyl)thio)ethyl)piperazinyl)acetonitrile (2e)

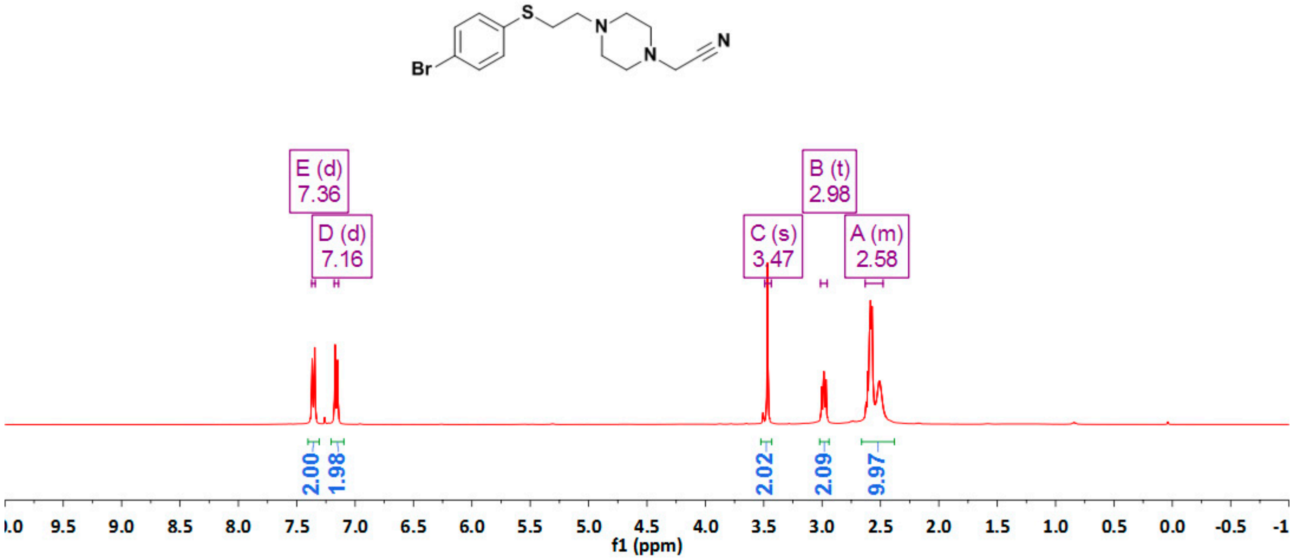

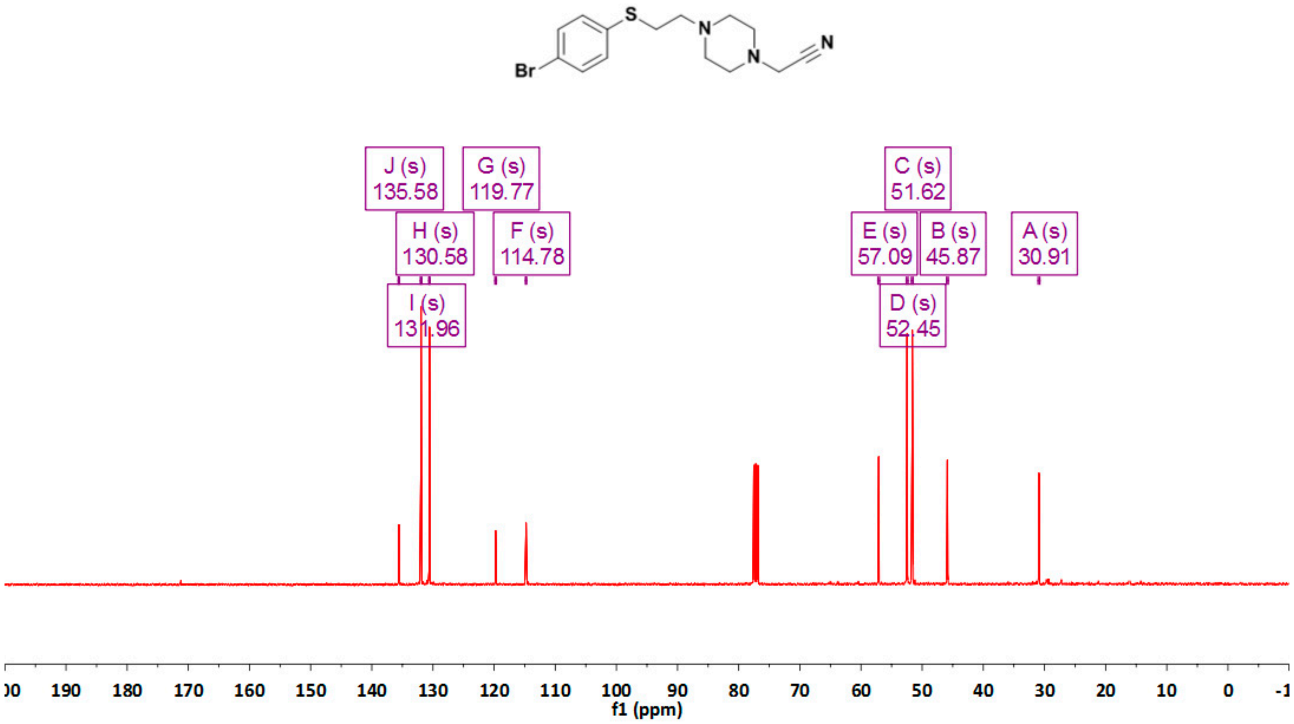

^{1}H NMR spectrum (400 MHz, $CDCl_3$) and ^{13}C NMR spectrum (101 MHz, $CDCl_3$) of 2-(4-(2-((4-chlorophenyl)thio)ethyl)piperazinyl)acetonitrile (2f)

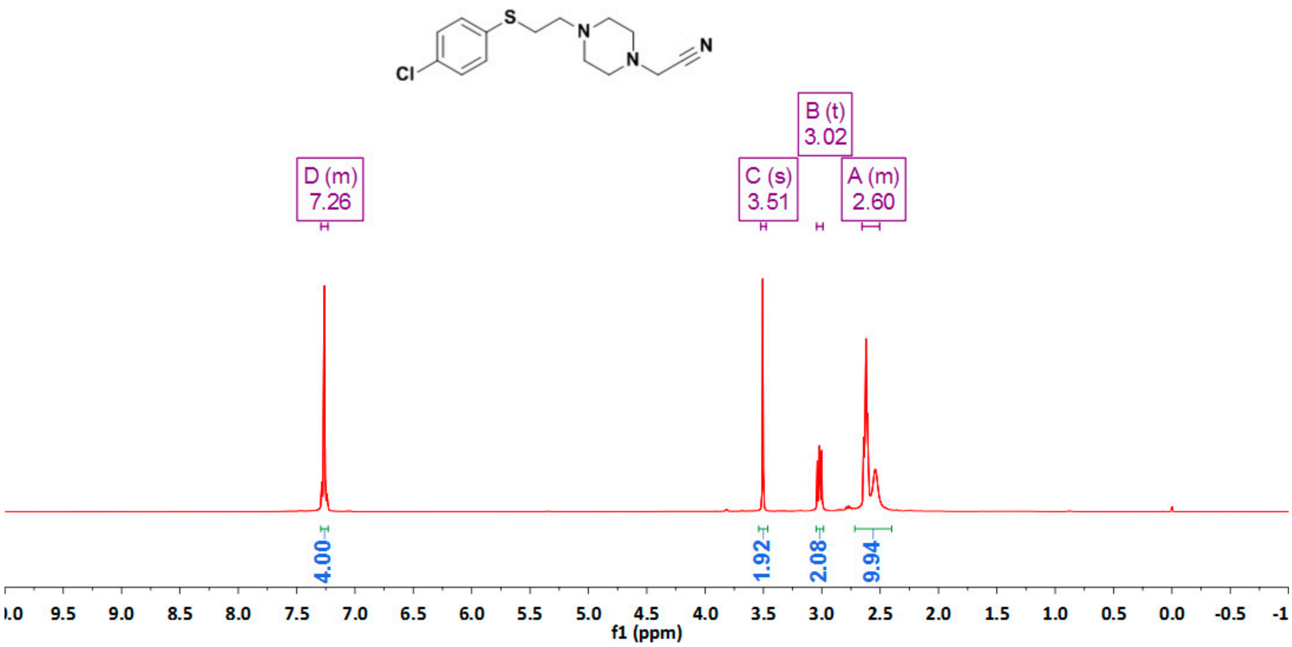

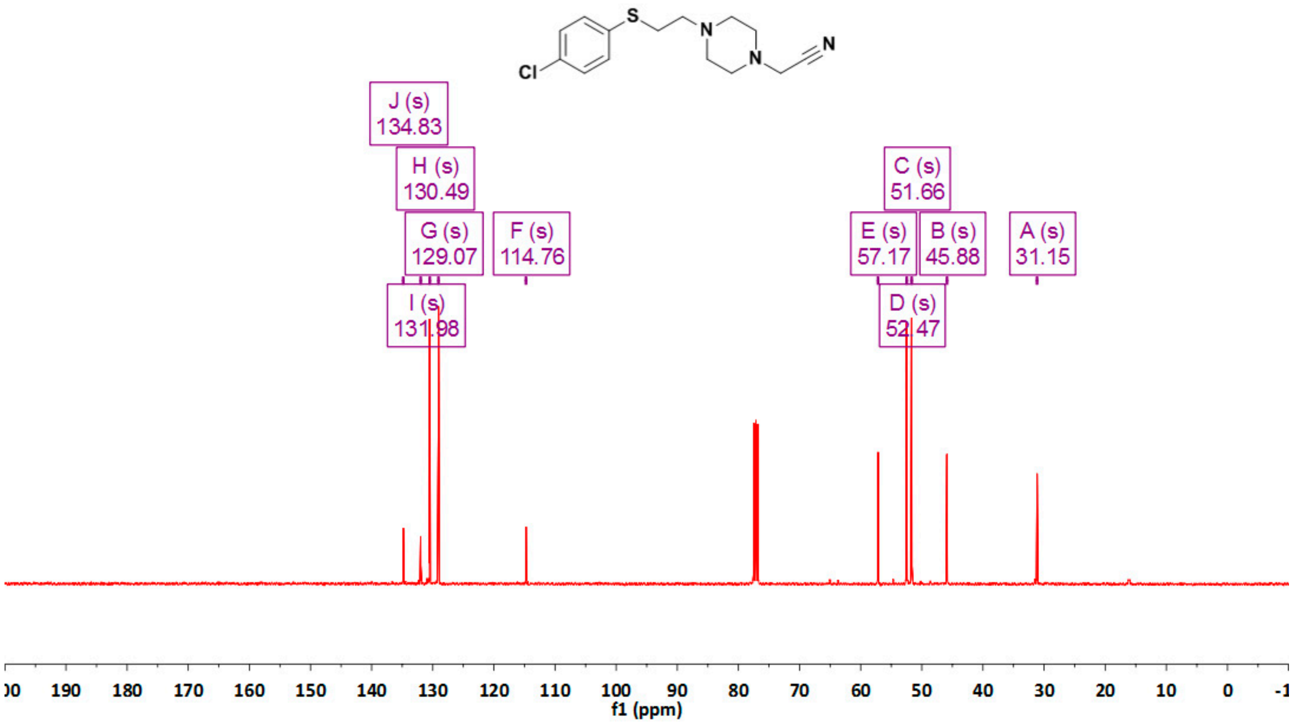

^{1}H NMR spectrum (400 MHz, $CDCl_3$) and ^{13}C NMR spectrum (101 MHz, $CDCl_3$) of 2-(4-(2-((4-fluorophenyl)thio)ethyl)piperazinyl)acetonitrile (2g)

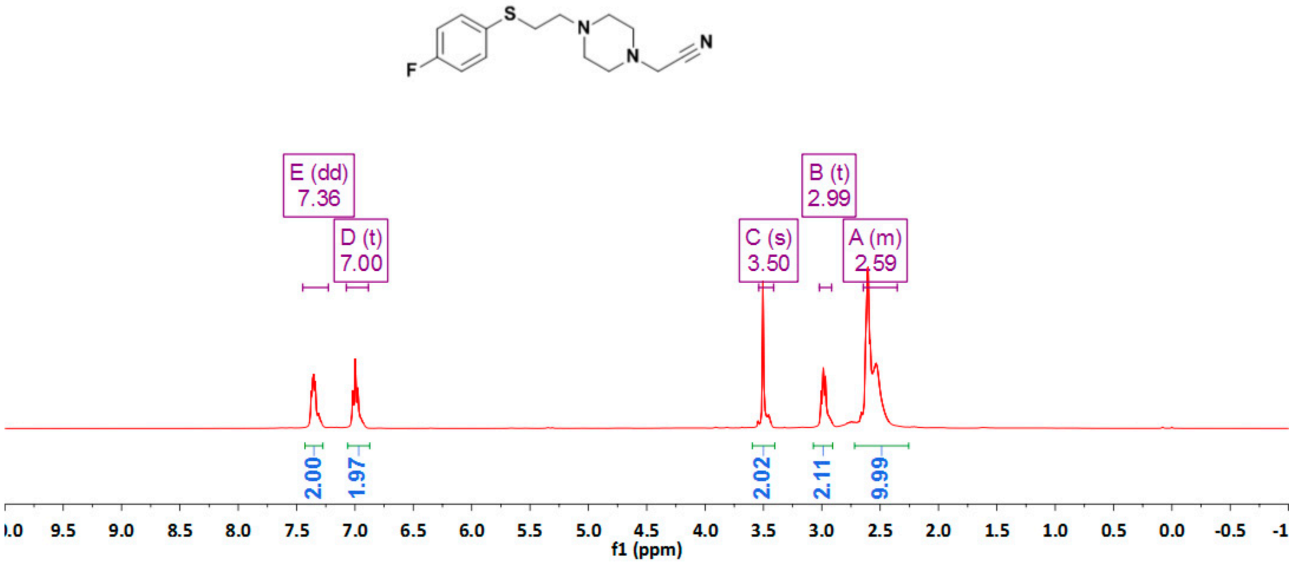

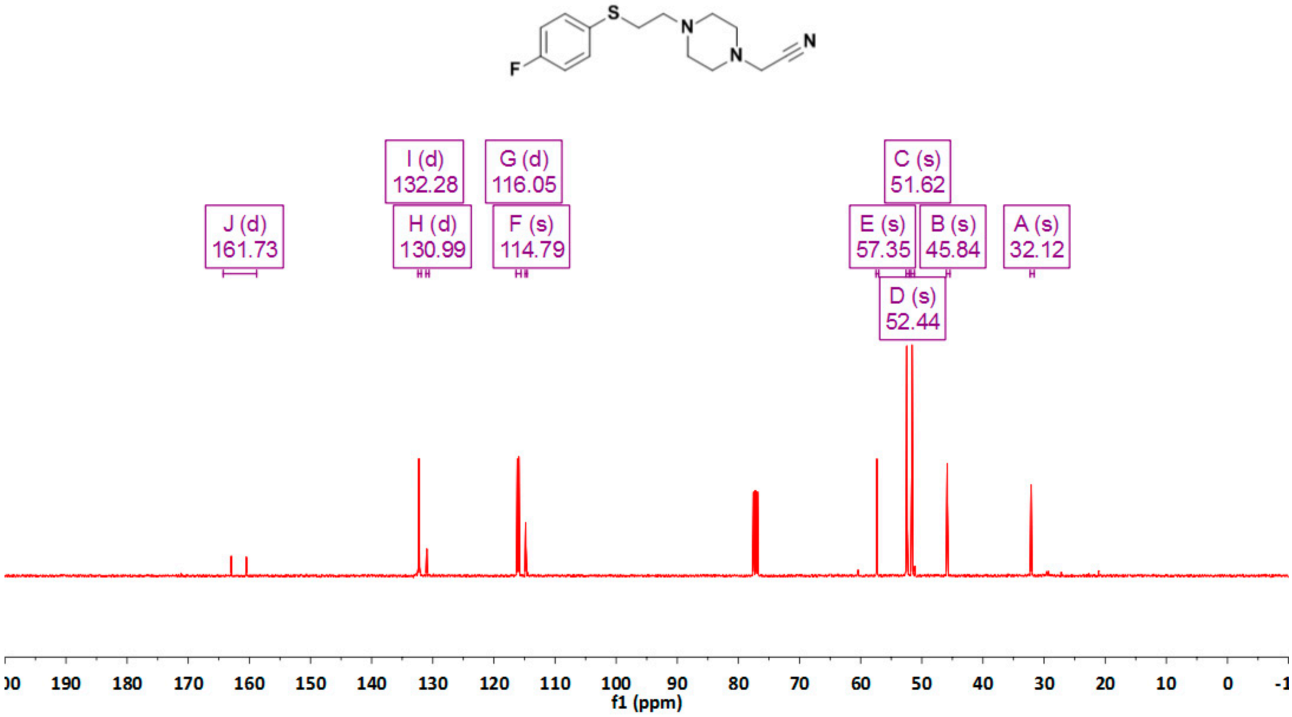

^{1}H NMR spectrum (400 MHz, $CDCl_3$) and ^{13}C NMR spectrum (101 MHz, $CDCl_3$) of 2-(4-(2-((4-(trifluoromethyl)phenyl)thio)ethyl)piperazinyl)acetonitrile (2h)

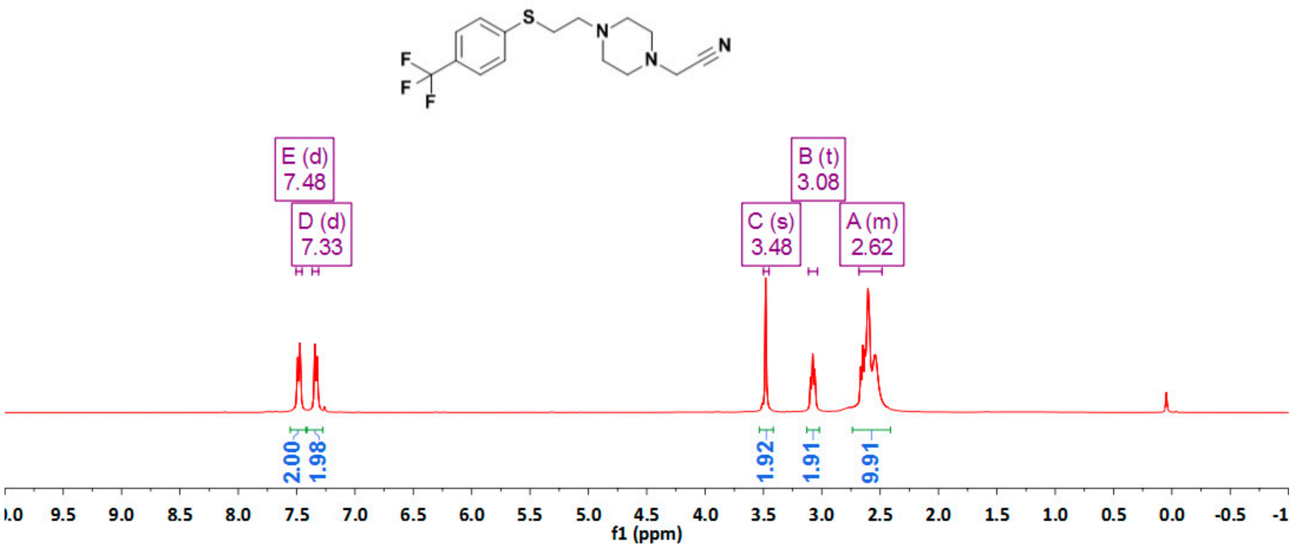

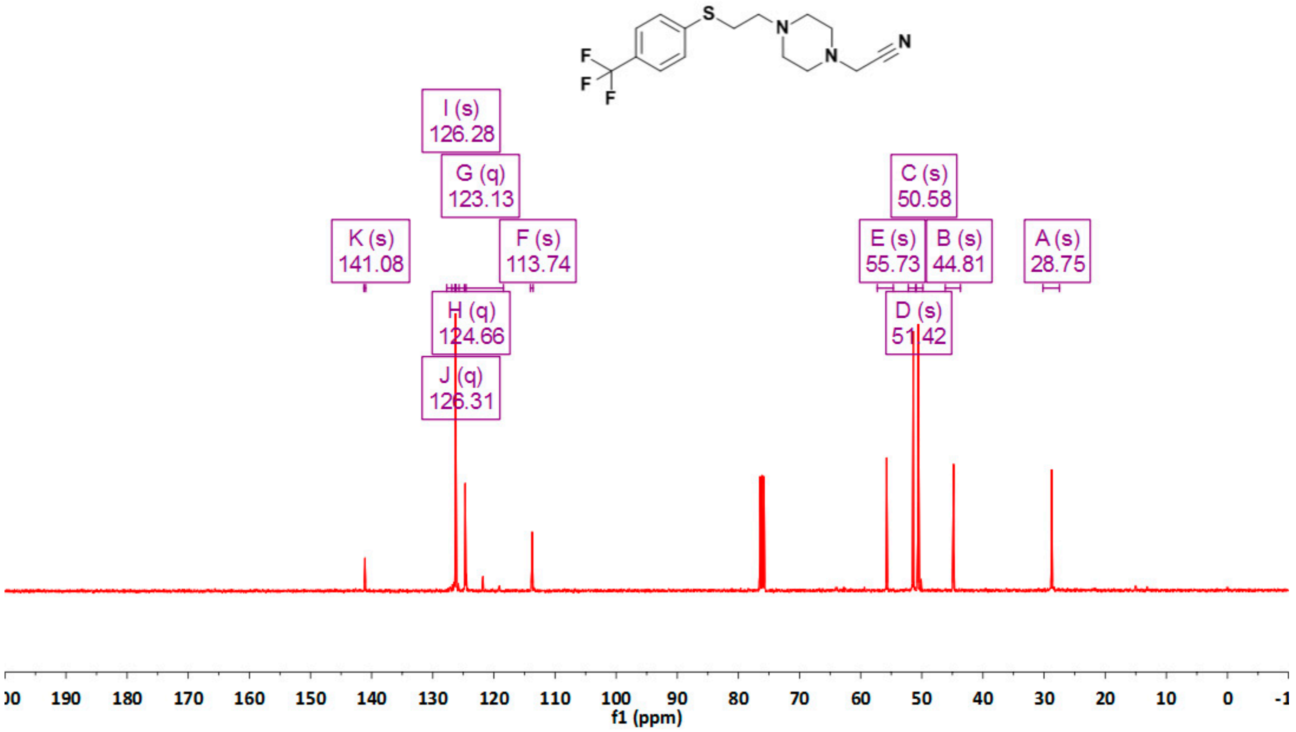

^{1}H NMR spectrum (400 MHz, $CDCl_3$) and ^{13}C NMR spectrum (101 MHz, $CDCl_3$) of 2-(4-(2-((3-methoxyphenyl)thio)ethyl)piperazinyl)acetonitrile (2i)

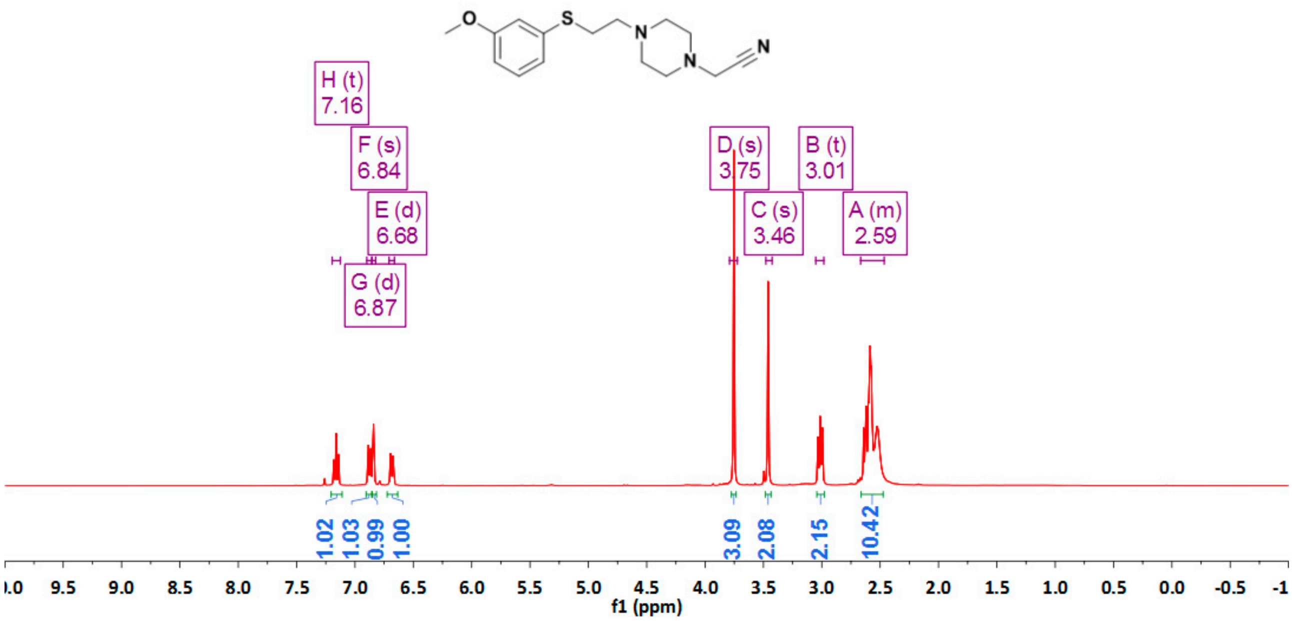

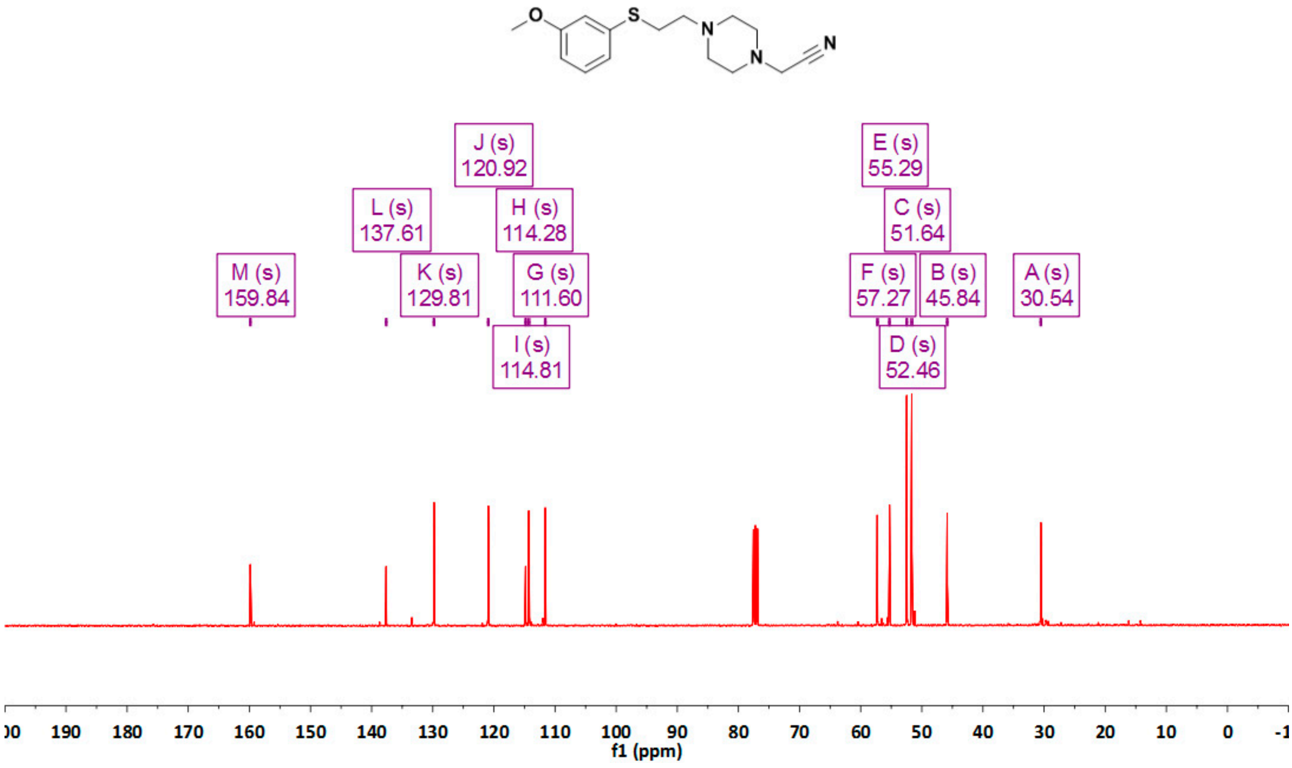

^{1}H NMR spectrum (400 MHz, $CDCl_3$) and ^{13}C NMR spectrum (101 MHz, $CDCl_3$) of 2-(4-(2-(m-tolylthio)ethyl)piperazinyl)acetonitrile (2j)

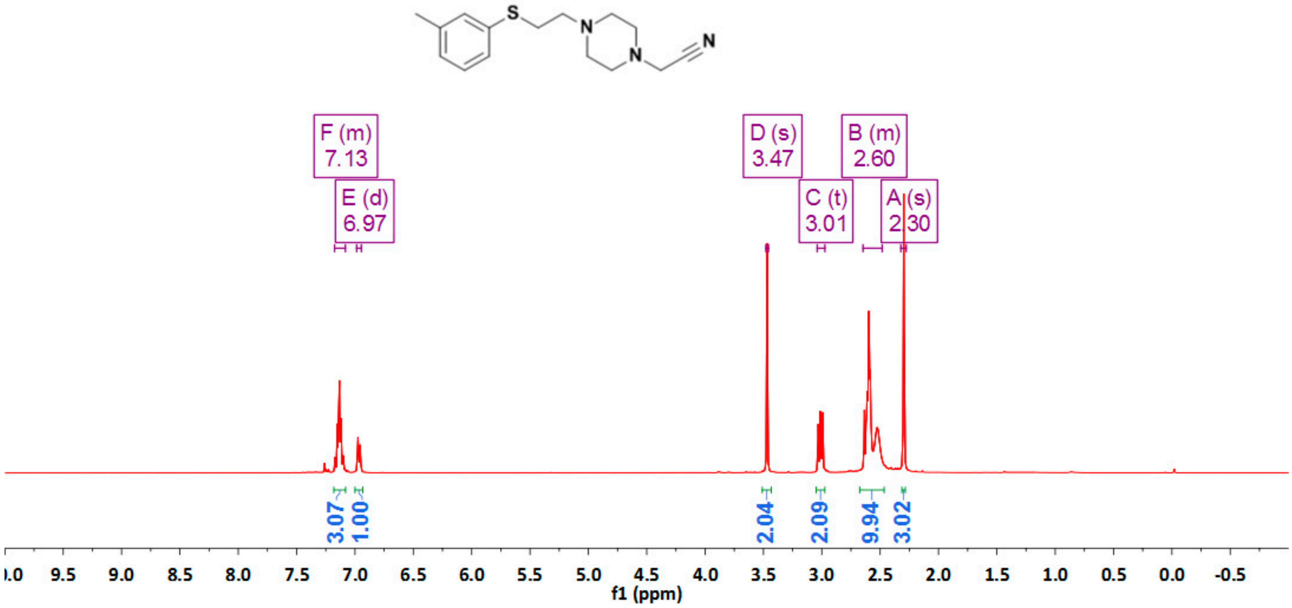

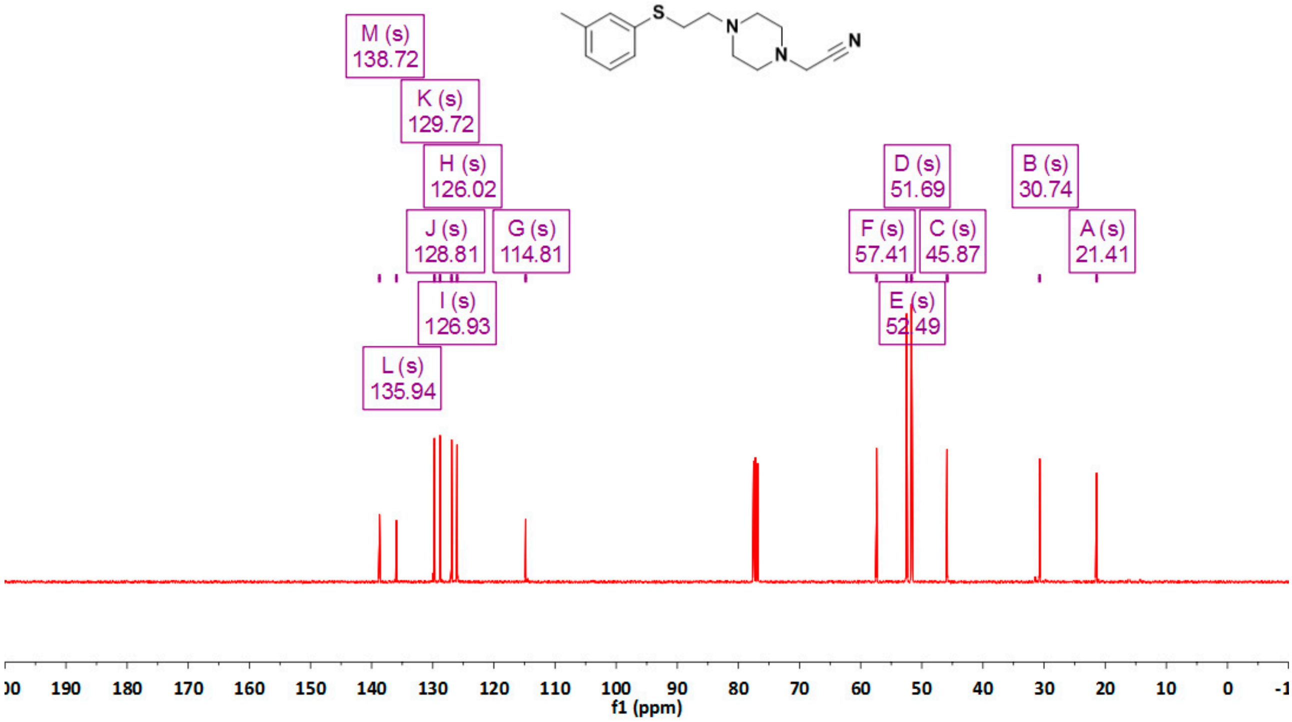

^{1}H NMR spectrum (400 MHz, $CDCl_3$) and ^{13}C NMR spectrum (101 MHz, $CDCl_3$) of 2-(4-(2-((3-bromophenyl)thio)ethyl)piperazinyl)acetonitrile (2k)

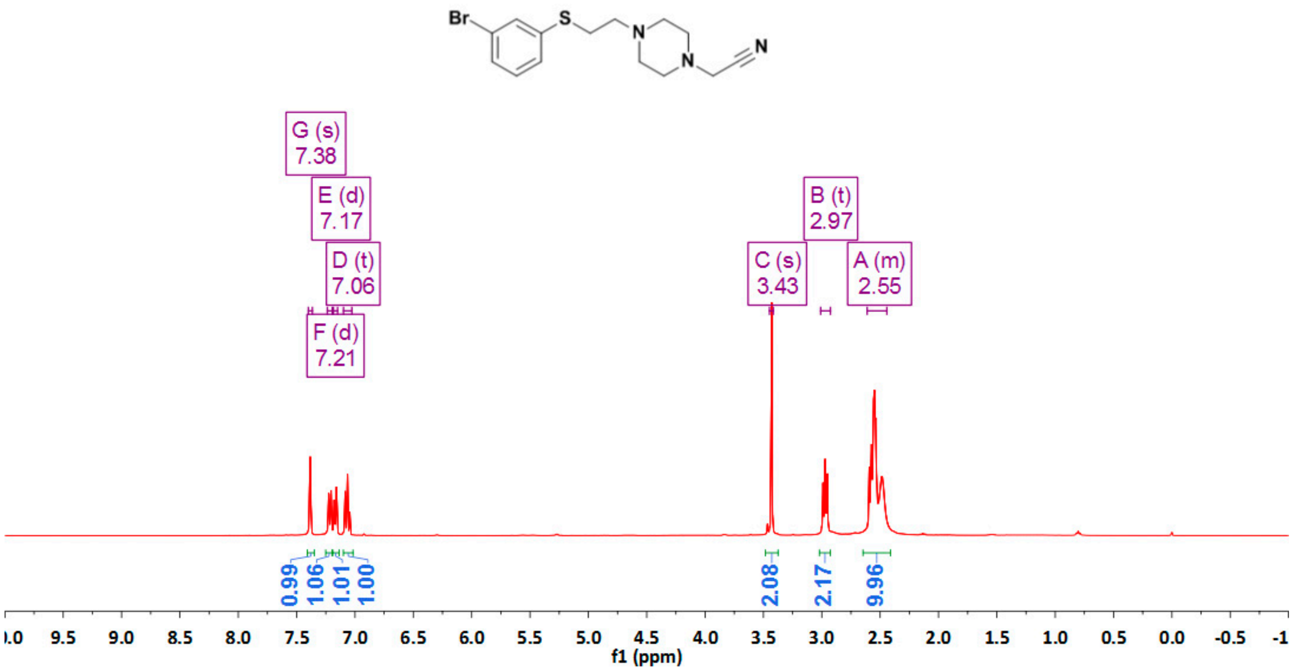

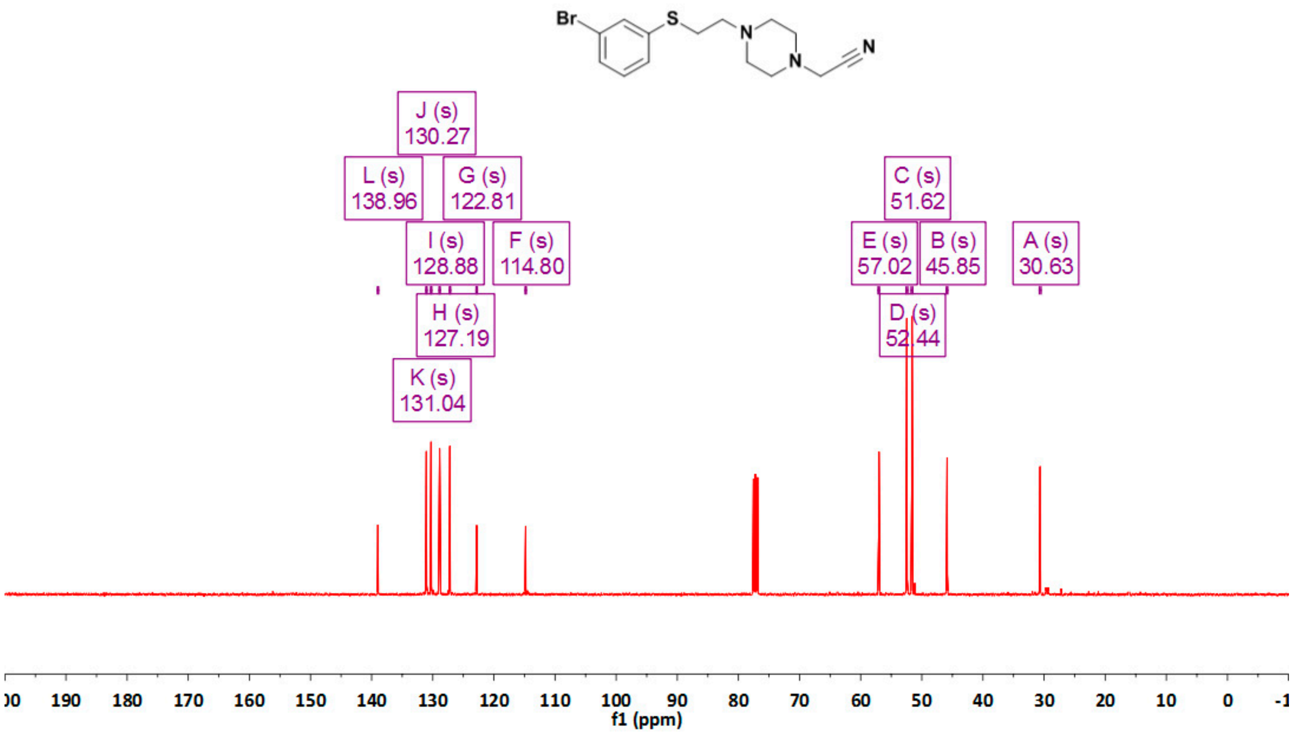

^{1}H NMR spectrum (400 MHz, $CDCl_3$) and ^{13}C NMR spectrum (101 MHz, $CDCl_3$) of 2-(4-(2-((3-chlorophenyl)thio)ethyl)piperazinyl)acetonitrile (2l)

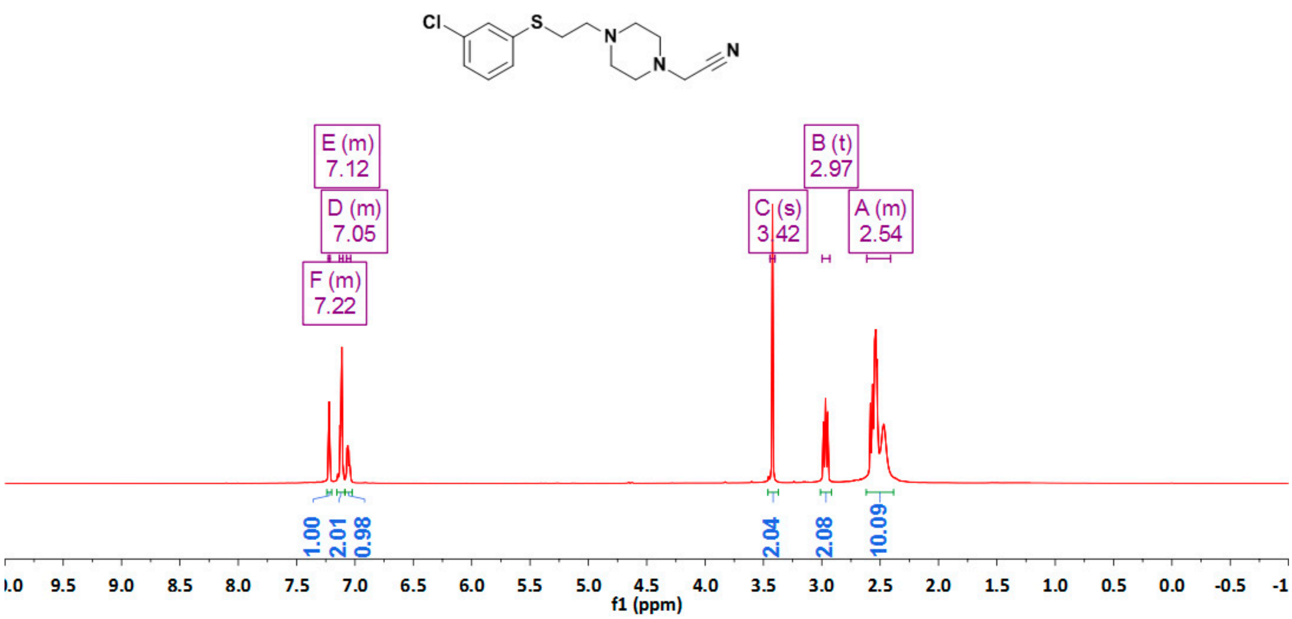

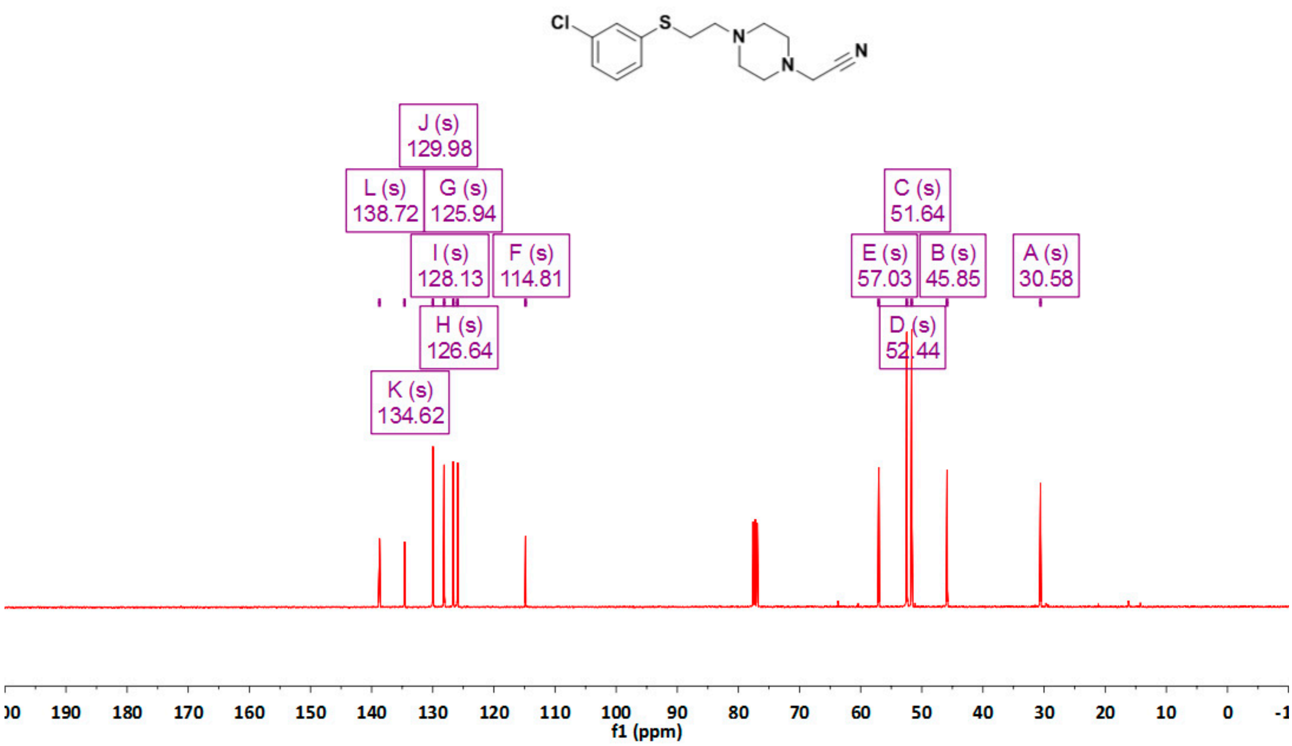

^{1}H NMR spectrum (400 MHz, CDCl$_3$) and ^{13}C NMR spectrum (101 MHz, CDCl$_3$) of 2-(4-(2-((3-fluorophenyl)thio)ethyl)piperazinyl)acetonitrile (2m)

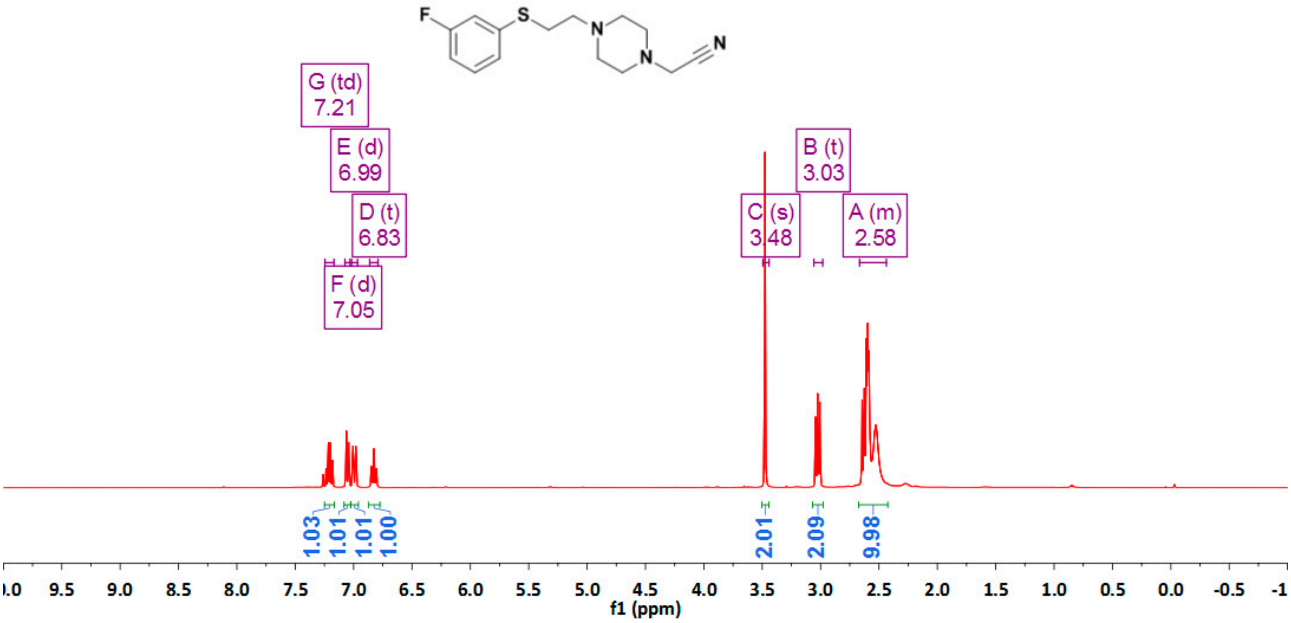

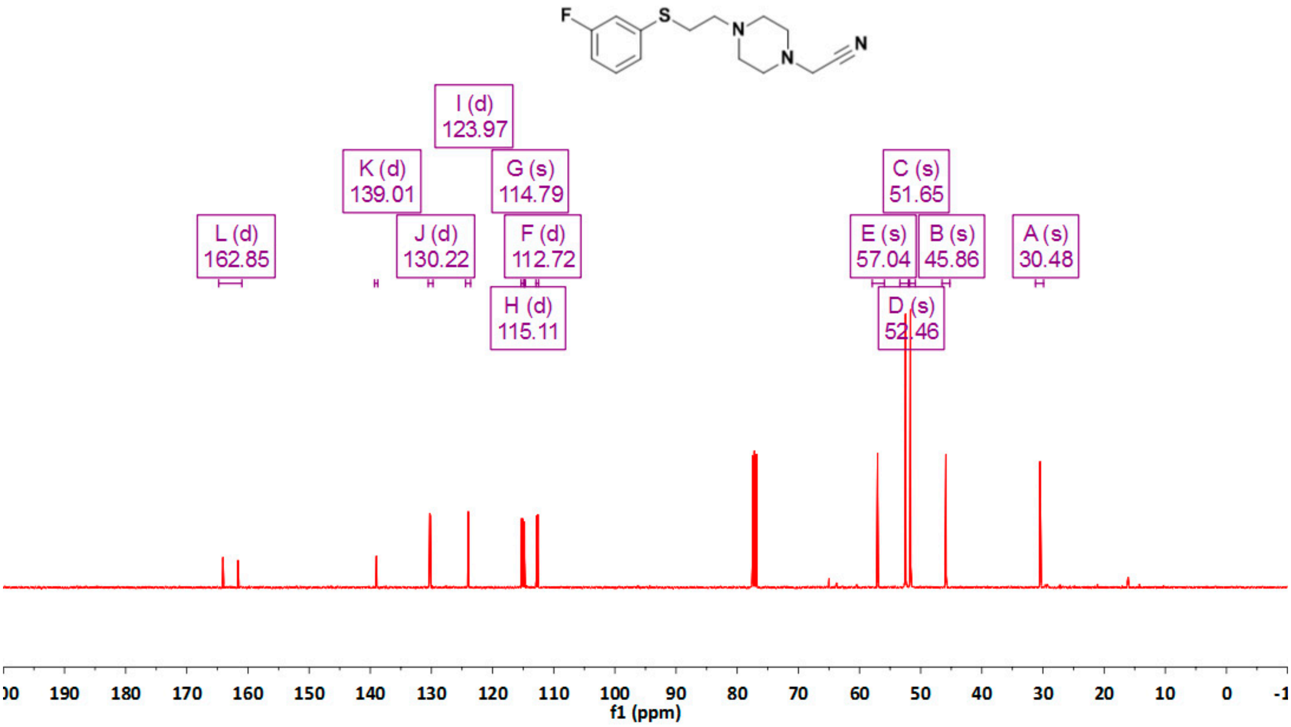

^{1}H NMR spectrum (400 MHz, $CDCl_3$) and ^{13}C NMR spectrum (101 MHz, $CDCl_3$) of 2-(4-(2-((2-methoxyphenyl)thio)ethyl)piperazinyl)acetonitrile (2n)

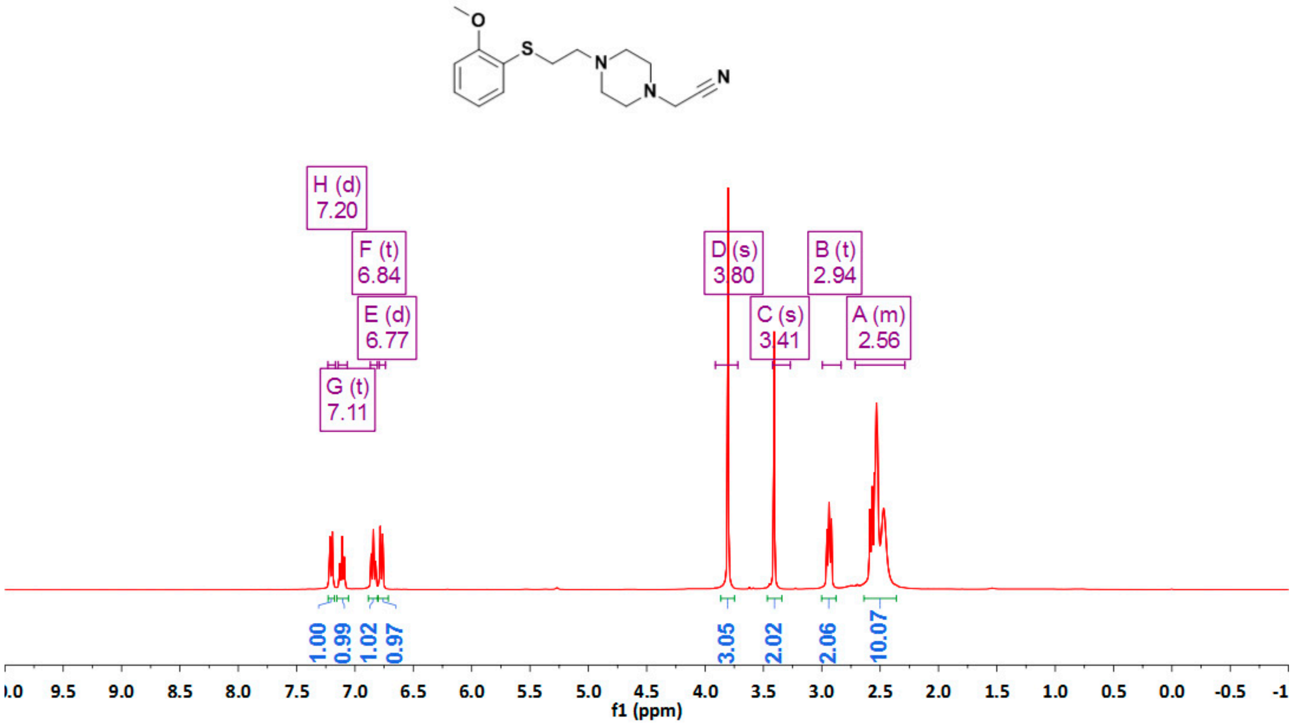

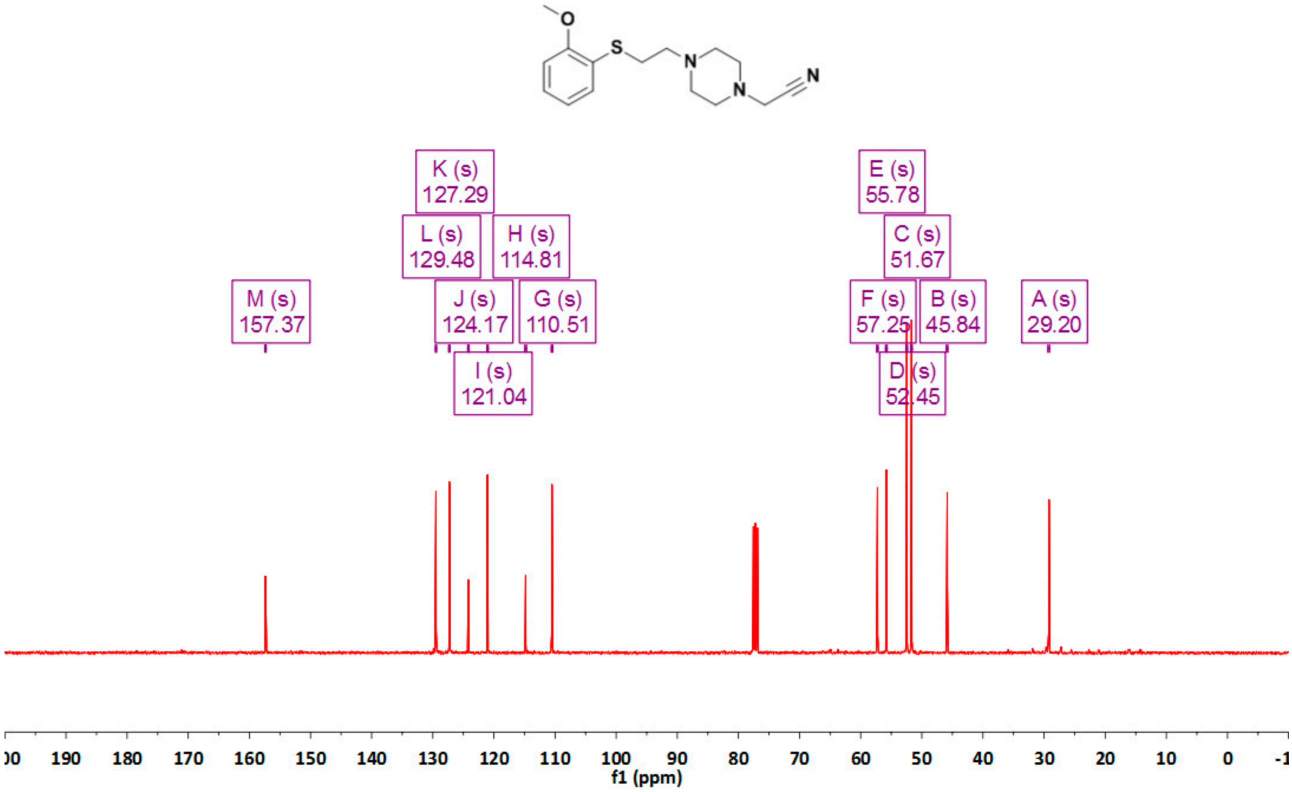

^{1}H NMR spectrum (400 MHz, $CDCl_3$) and ^{13}C NMR spectrum (101 MHz, $CDCl_3$) of 2-(4-(2-(o-tolylthio)ethyl)piperazinyl)acetonitrile (2o)

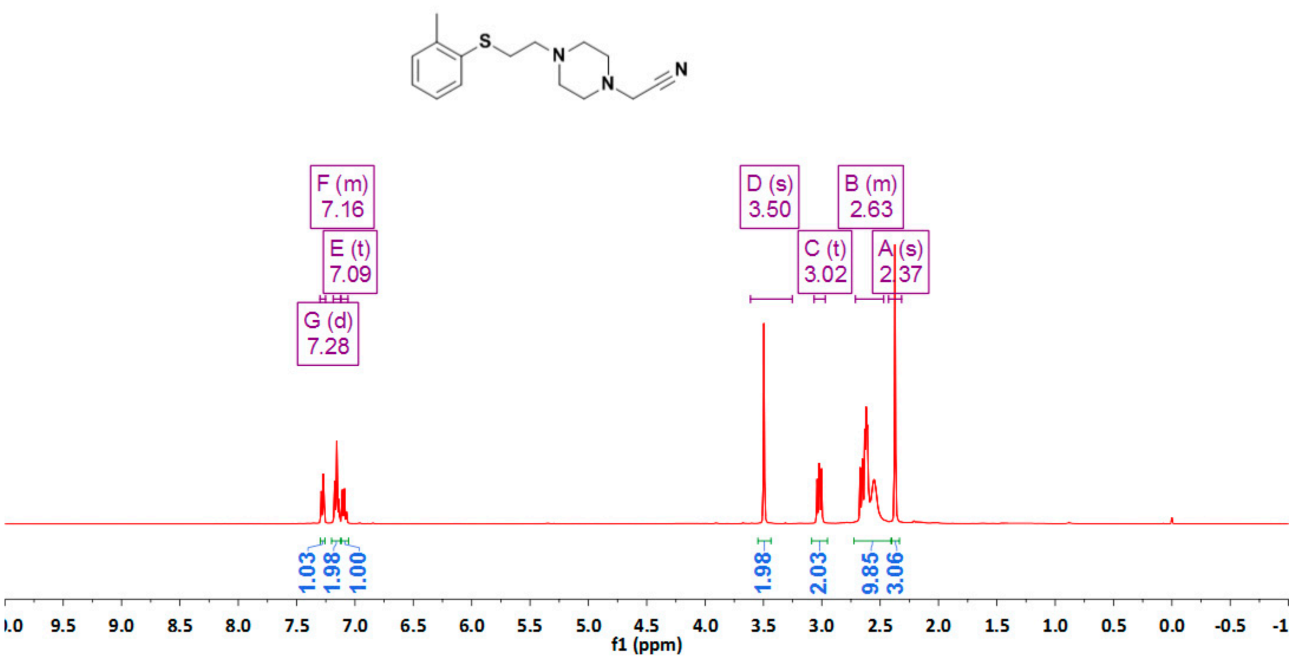

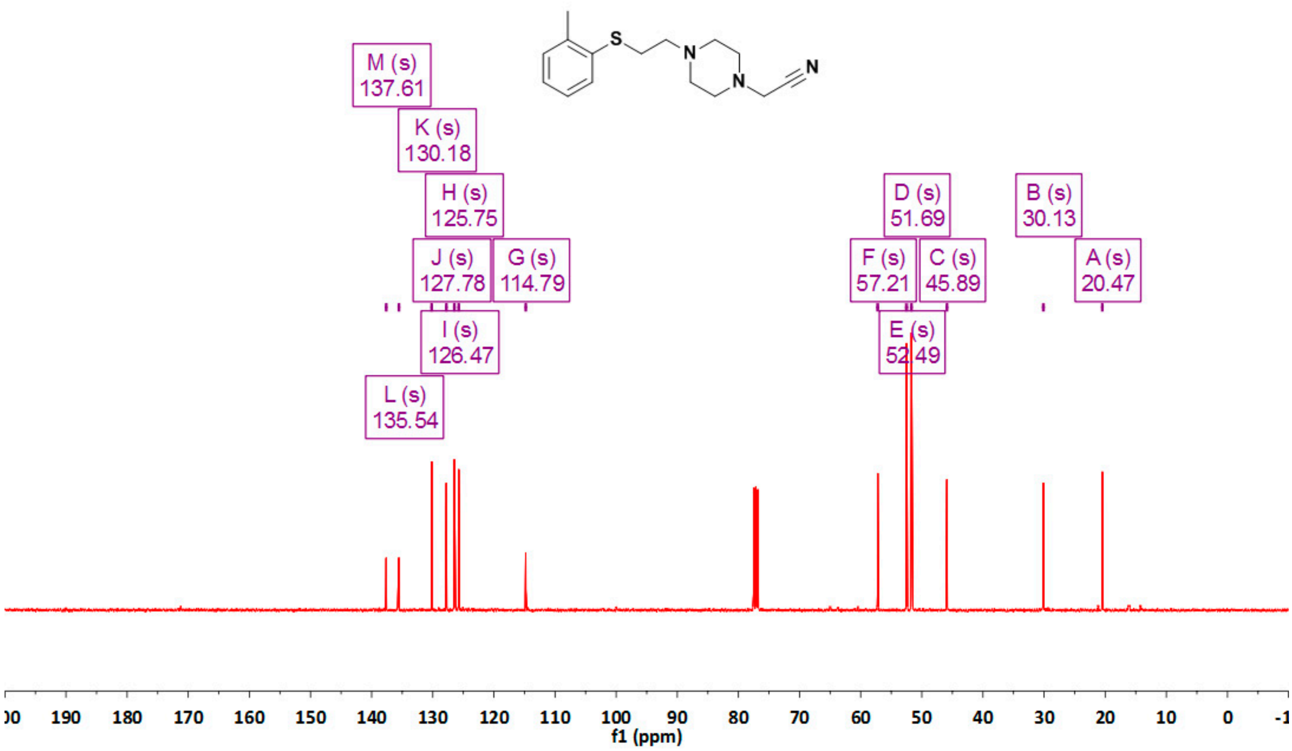

^{1}H NMR spectrum (400 MHz, $CDCl_3$) and ^{13}C NMR spectrum (101 MHz, $CDCl_3$) of 2-(4-(2-((2-bromophenyl)thio)ethyl)piperazinyl)acetonitrile (2p)

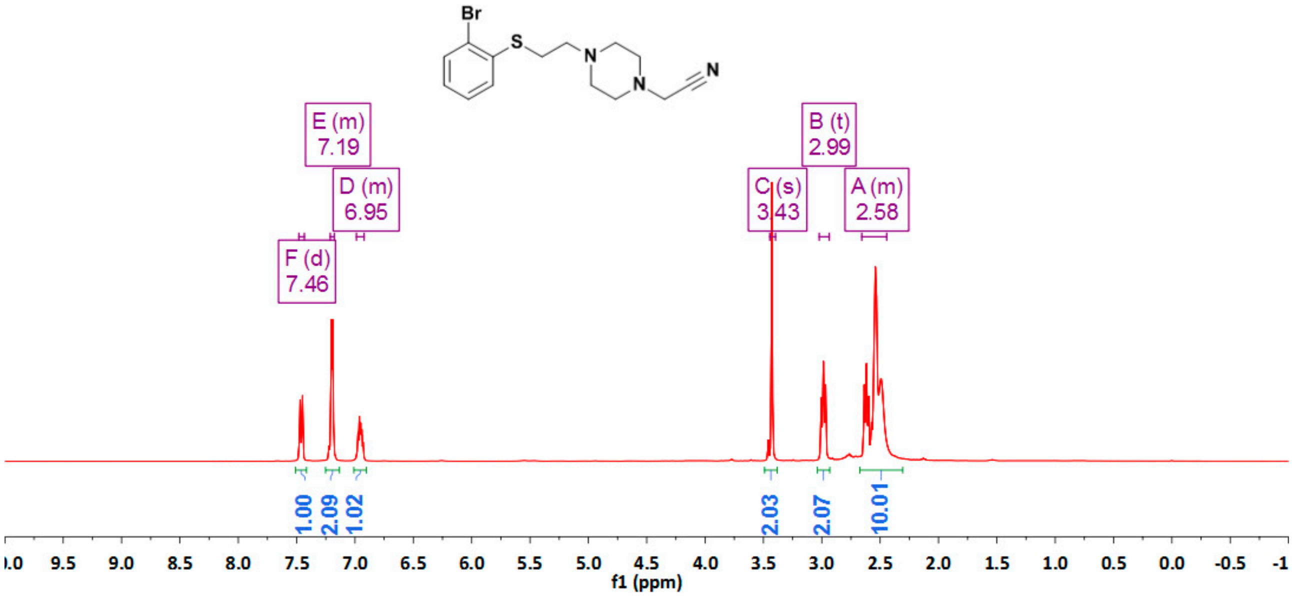

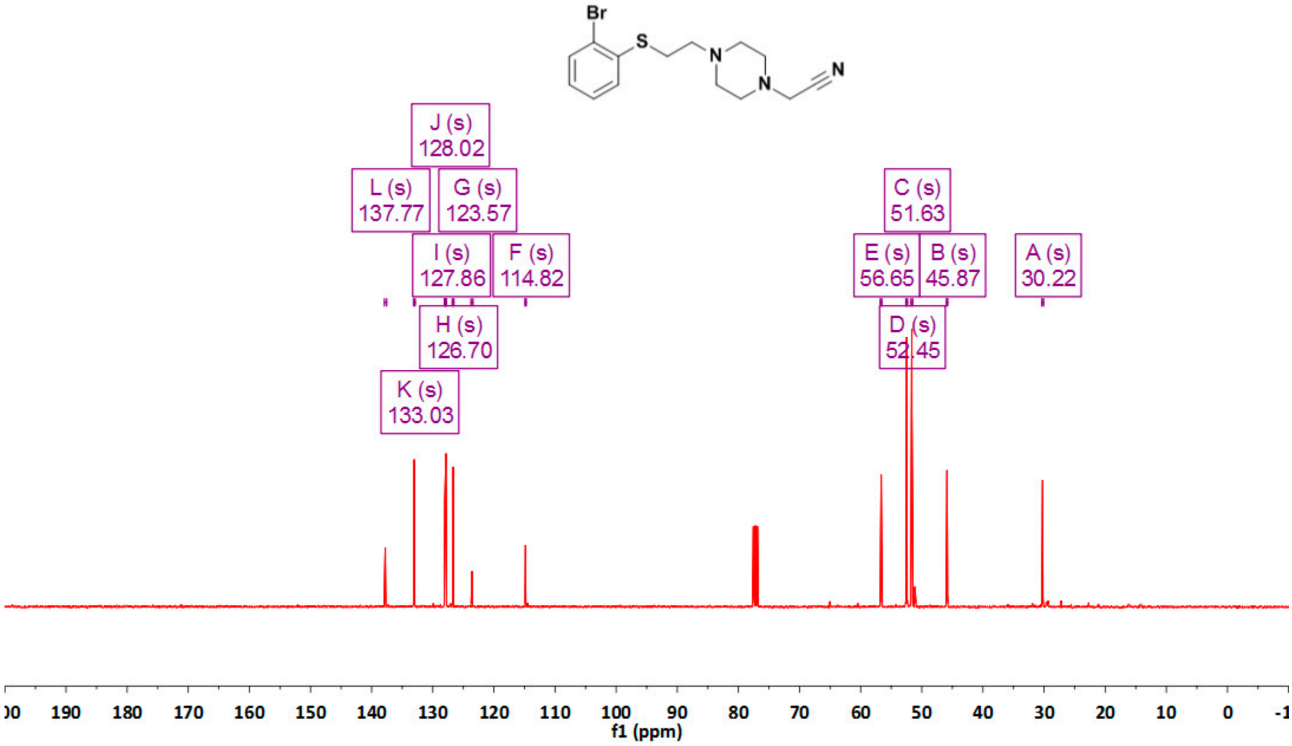

^{1}H NMR spectrum (400 MHz, $CDCl_3$) and ^{13}C NMR spectrum (101 MHz, $CDCl_3$) of 2-(4-(2-((2-chlorophenyl)thio)ethyl)piperazinyl)acetonitrile (2q)

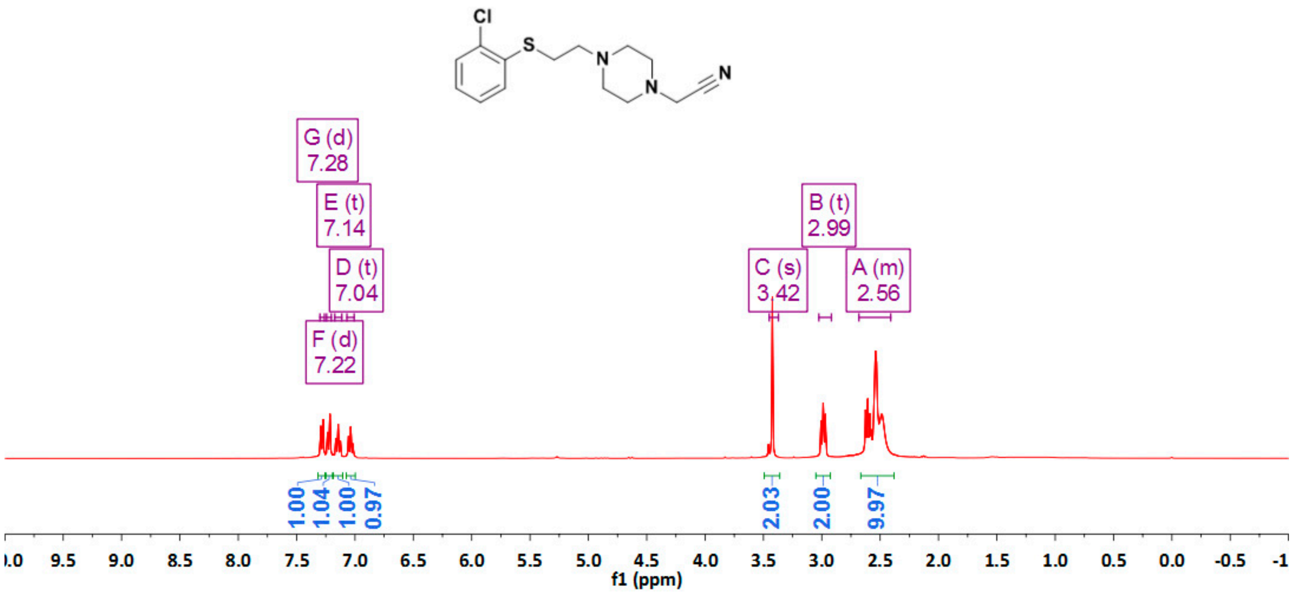

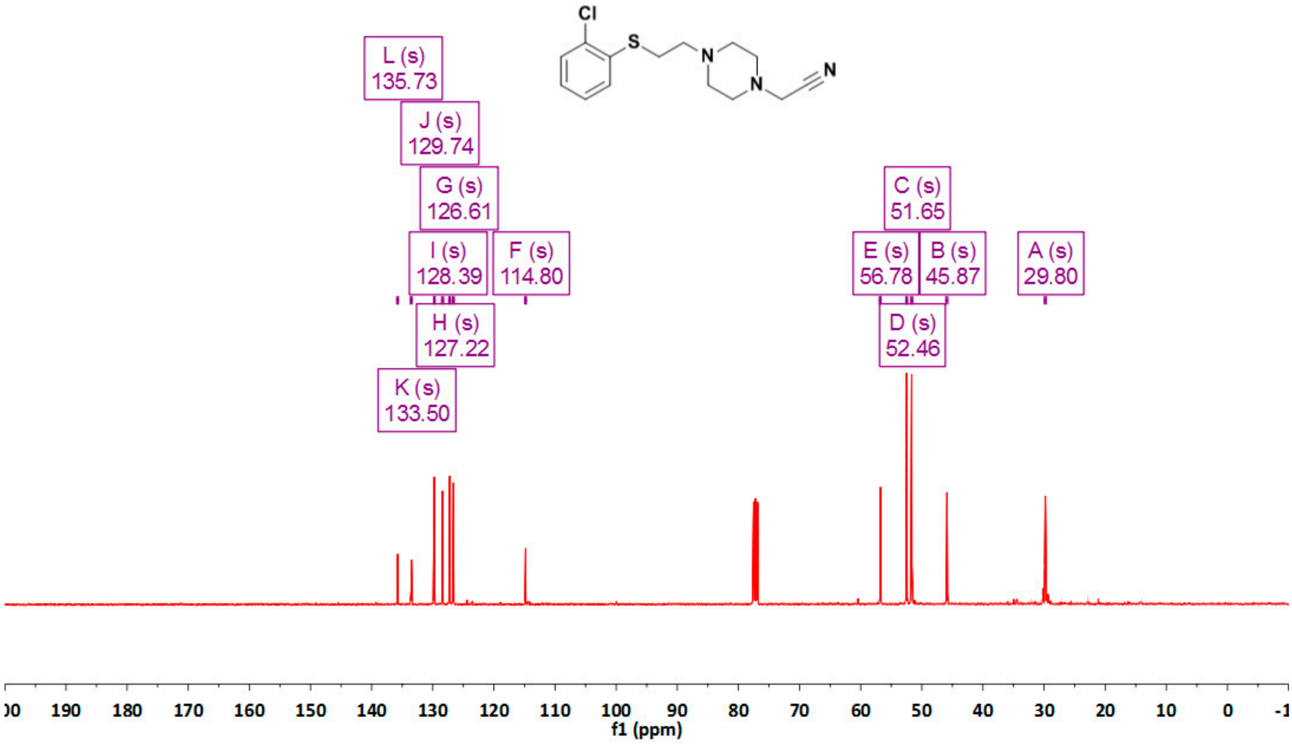

^{1}H NMR spectrum (400 MHz, $CDCl_3$) and ^{13}C NMR spectrum (101 MHz, $CDCl_3$) of 2-(4-(2-((2-fluorophenyl)thio)ethyl)piperazinyl)acetonitrile (2r)

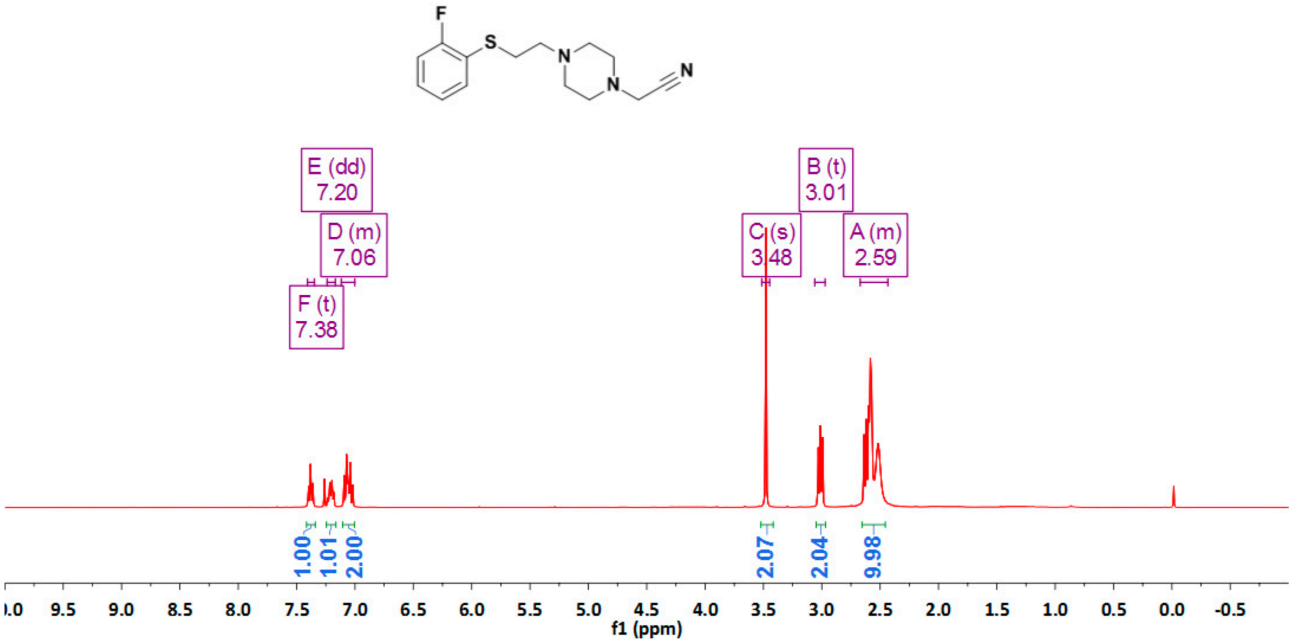

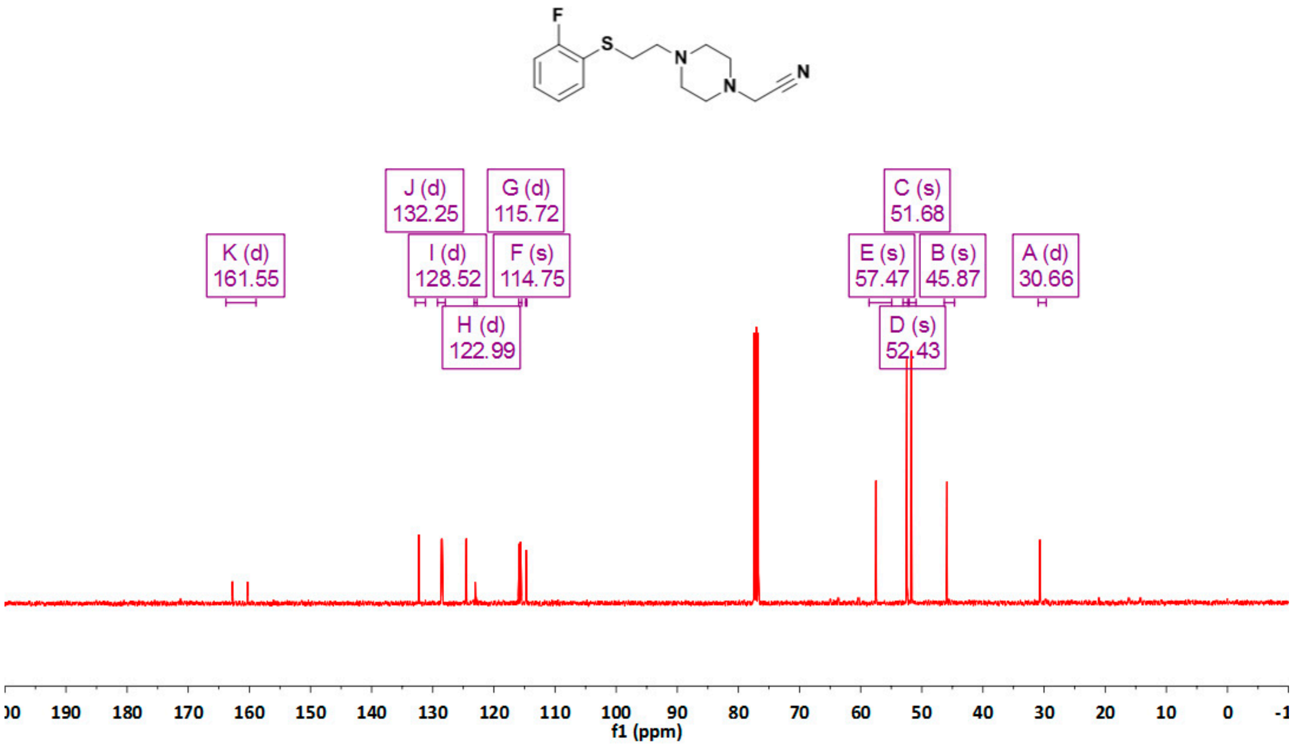

^{1}H NMR spectrum (400 MHz, $CDCl_3$) and ^{13}C NMR spectrum (101 MHz, $CDCl_3$) of 2-(4-(2-((2,4-dimethylphenyl)thio)ethyl)piperazinyl)acetonitrile (2s)

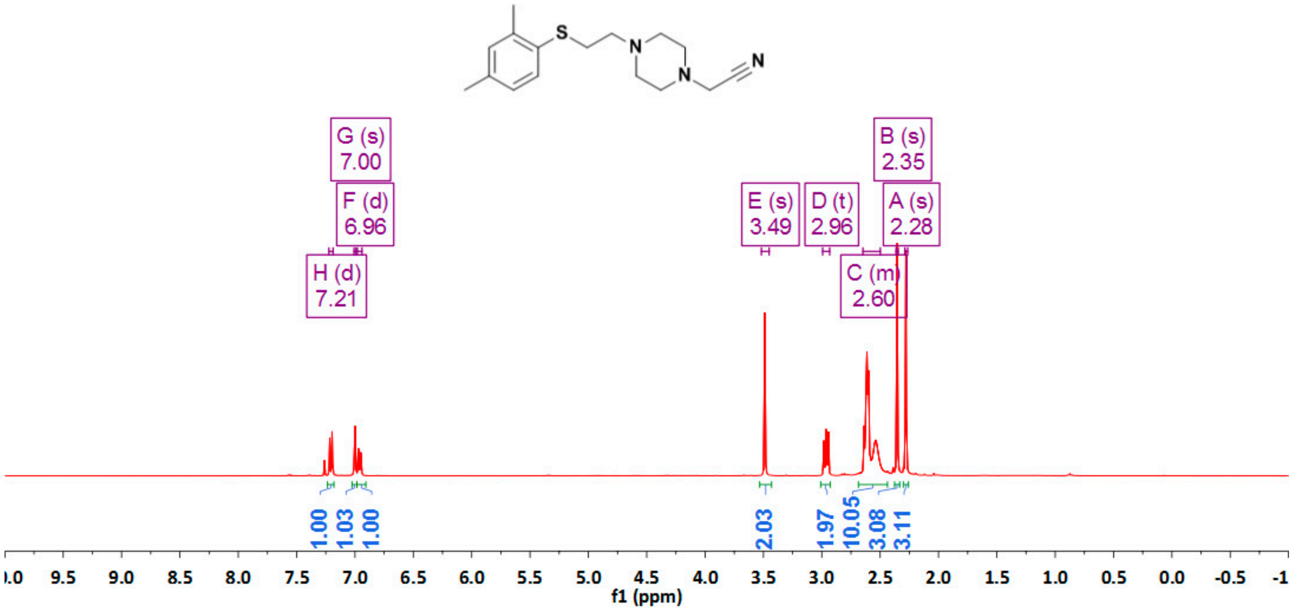

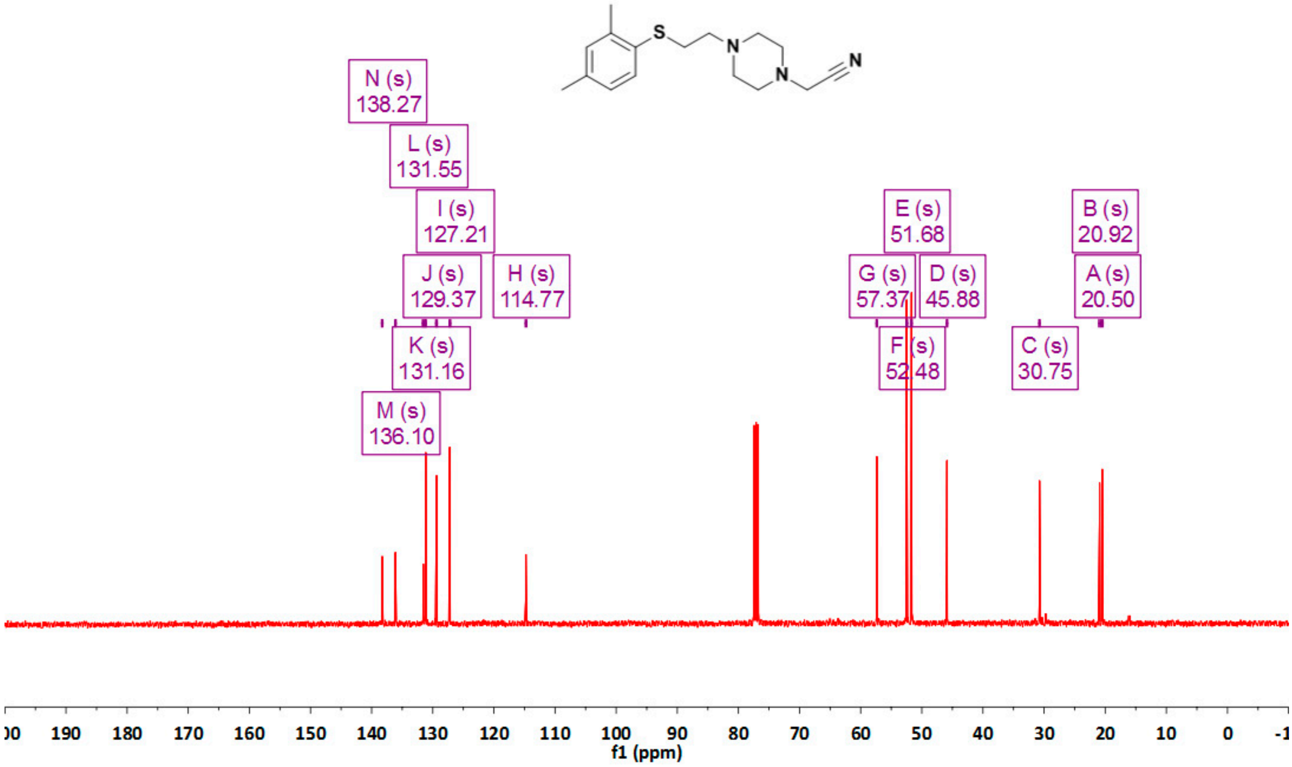

^{1}H NMR spectrum (400 MHz, $CDCl_3$) and ^{13}C NMR spectrum (101 MHz, $CDCl_3$) of 2-(4-(2-((2,5-dimethylphenyl)thio)ethyl)piperazinyl)acetonitrile (2t)

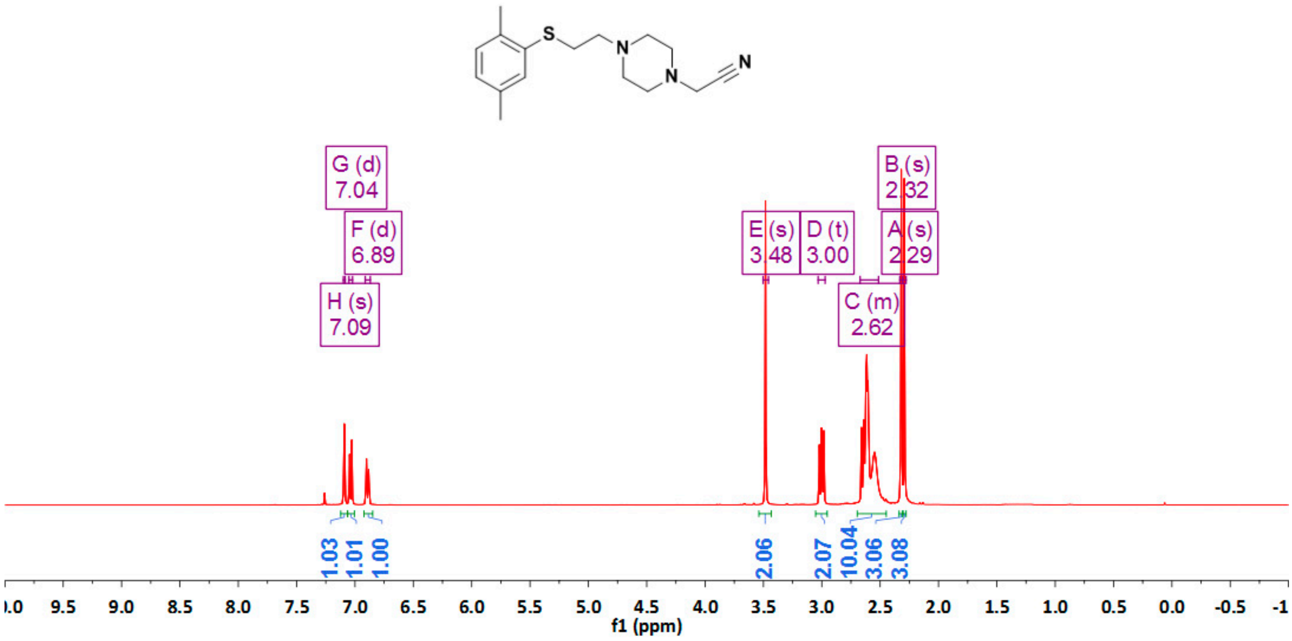

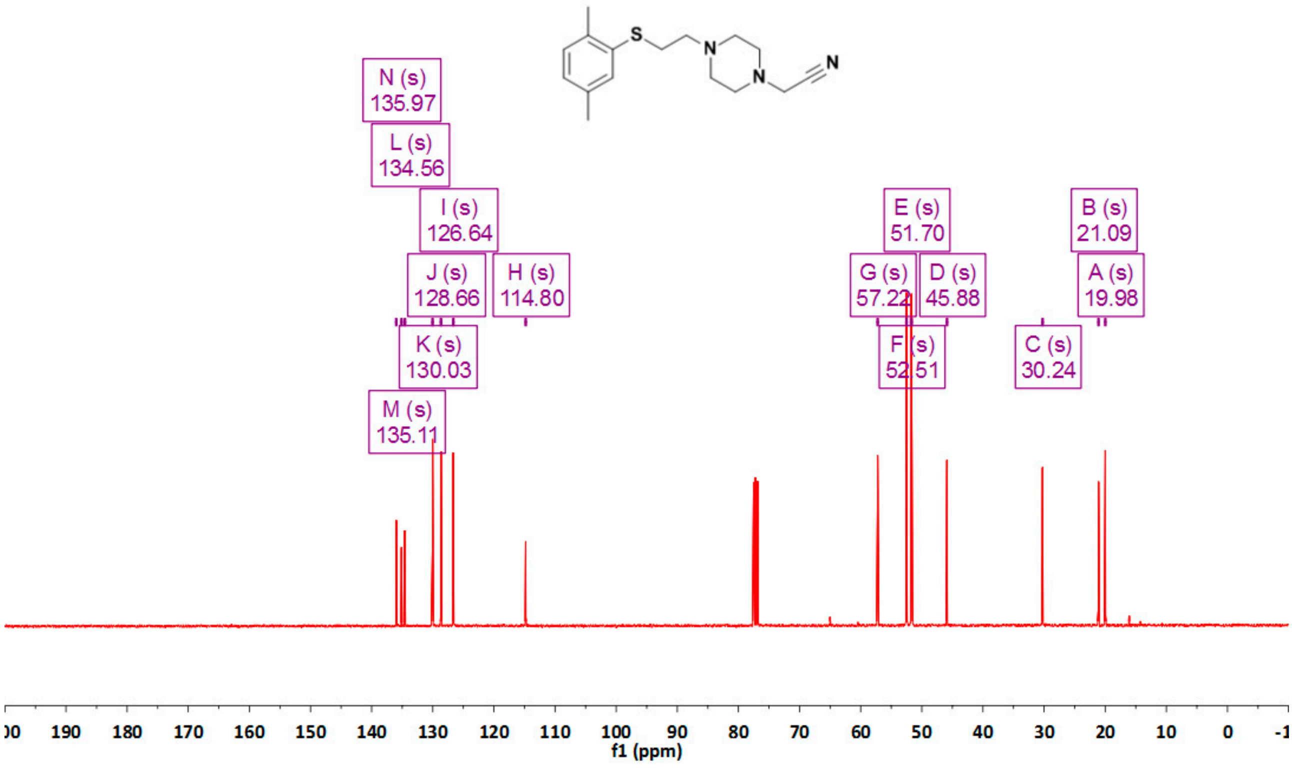

^{1}H NMR spectrum (400 MHz, $CDCl_3$) and ^{13}C NMR spectrum (101 MHz, $CDCl_3$) of 2-(4-(2-((3,5-dimethylphenyl)thio)ethyl)piperazinyl)acetonitrile (2u)

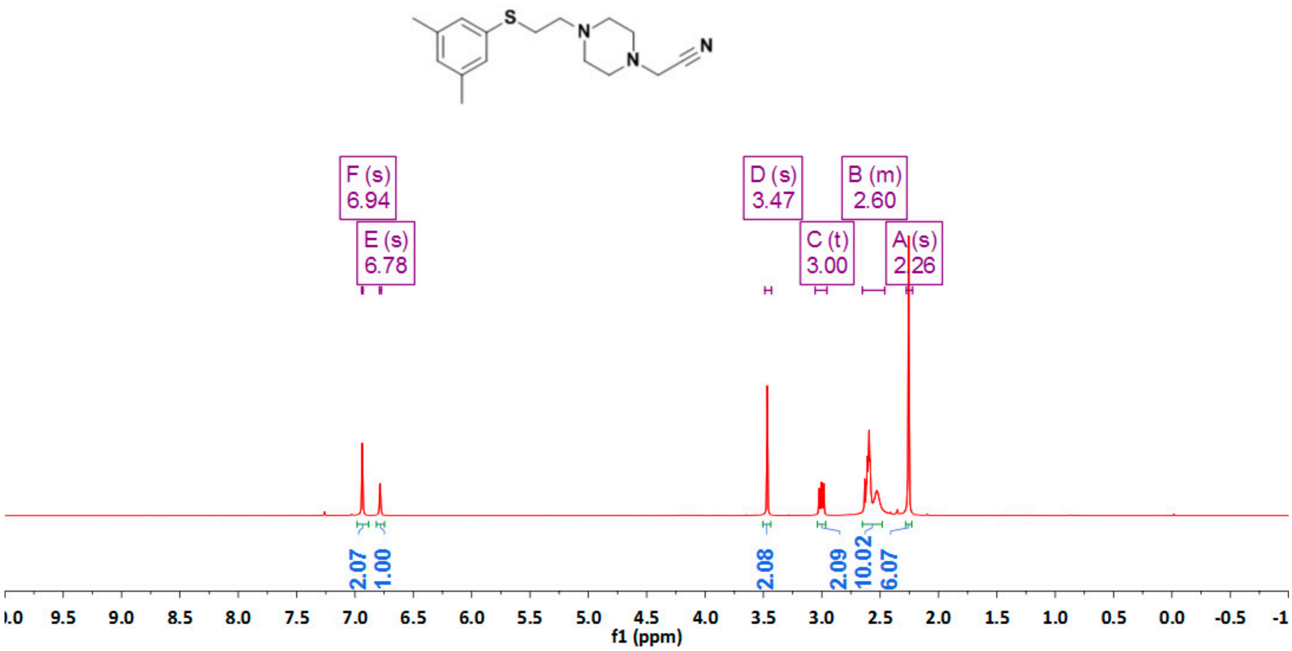

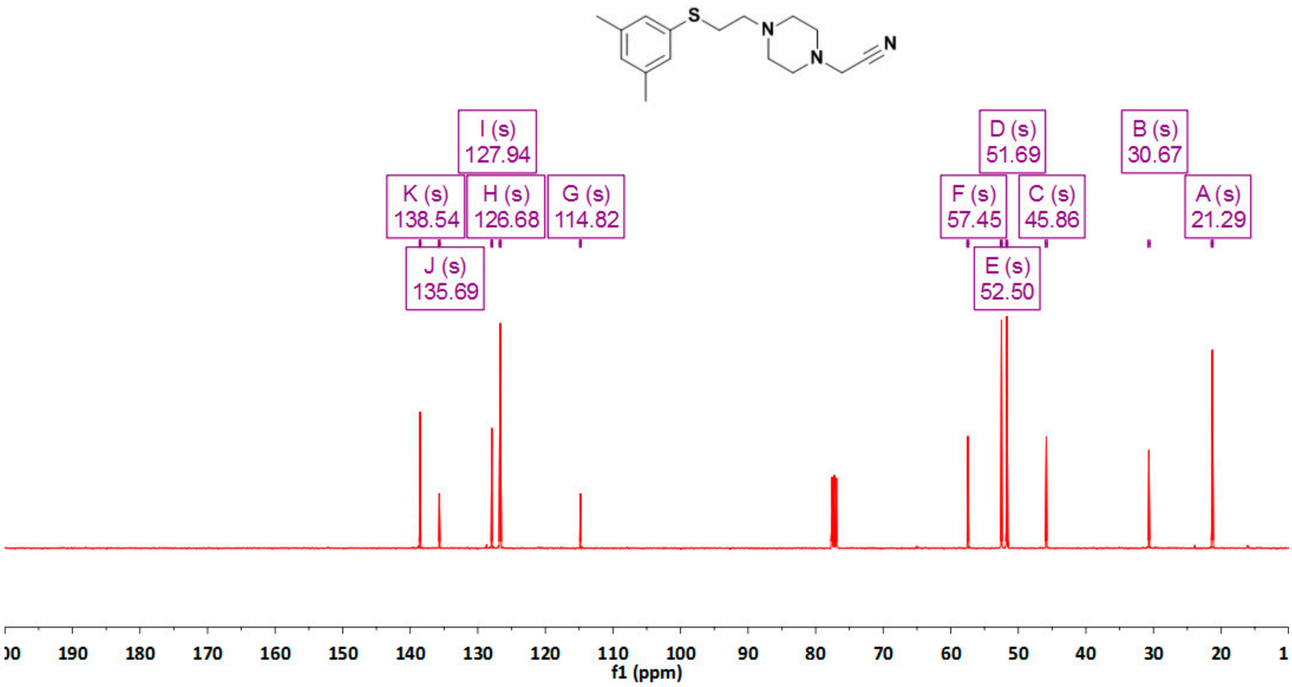

^{1}H NMR spectrum (400 MHz, CDCl$_3$) and ^{13}C NMR spectrum (101 MHz, CDCl$_3$) of 2-(4-(2-(naphthalen-2-ylthio)ethyl)piperazinyl)acetonitrile (2v)

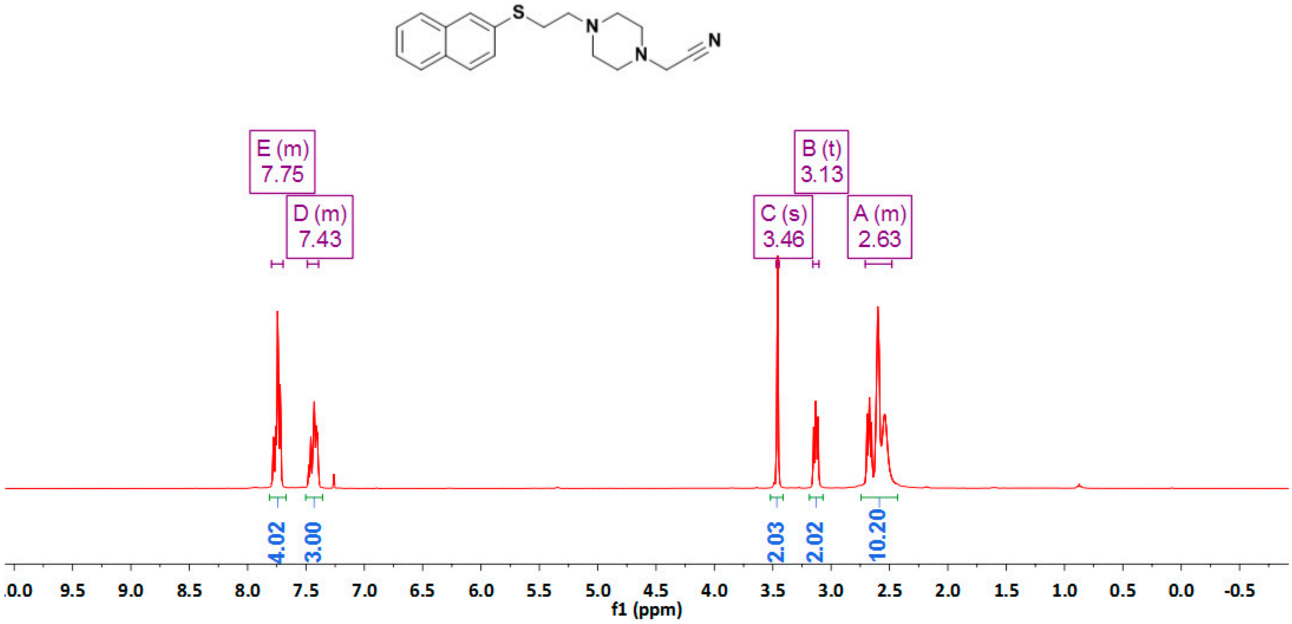

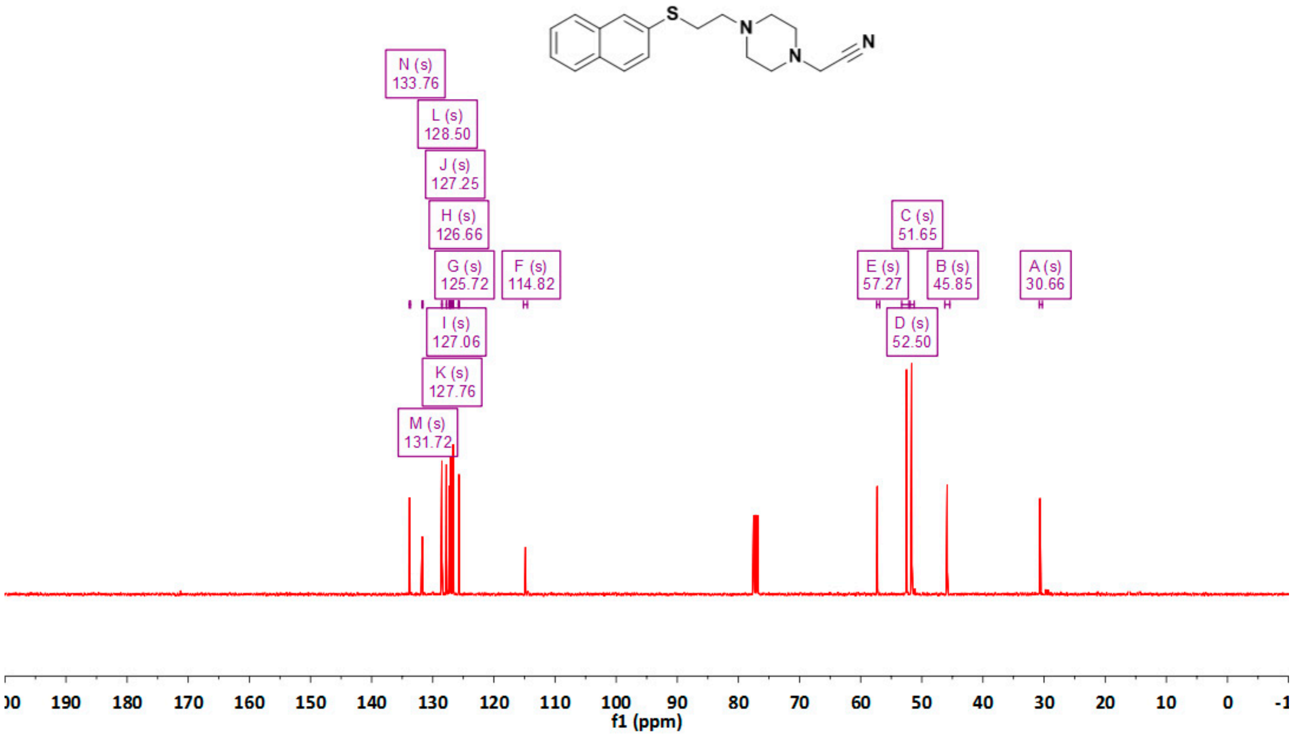

^{1}H NMR spectrum (400 MHz, $CDCl_3$) and ^{13}C NMR spectrum (101 MHz, $CDCl_3$) of 2-(4-(2-(pyridin-2-ylthio)ethyl)piperazinyl)acetonitrile (2w)

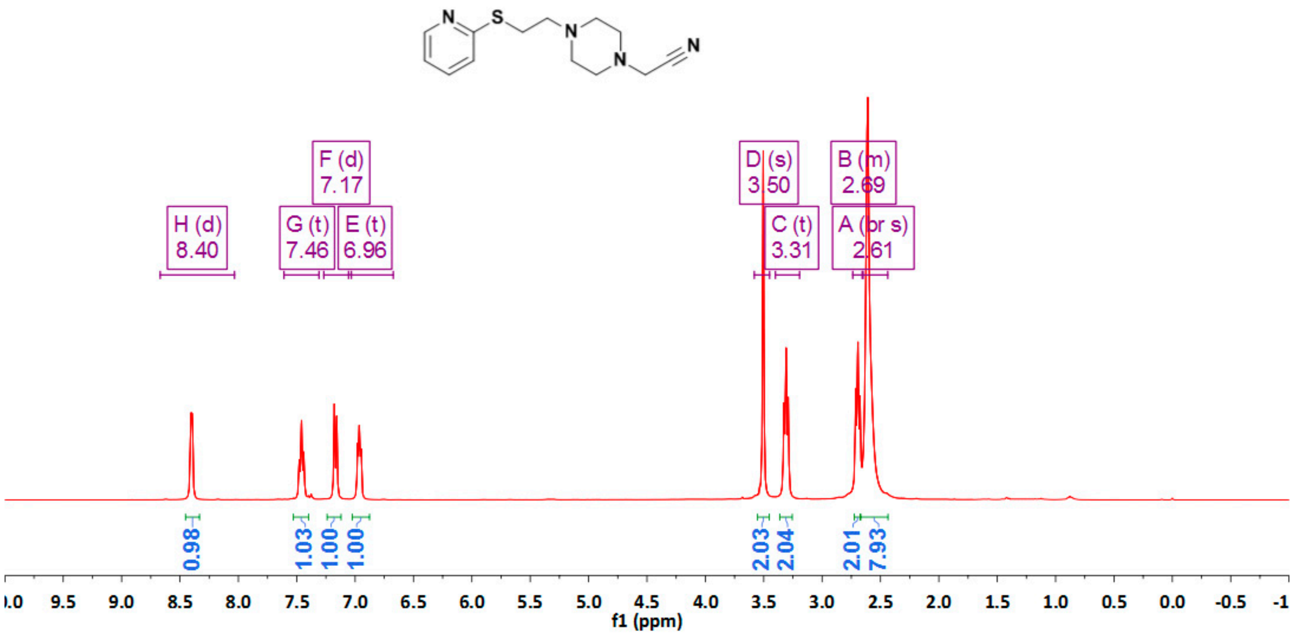

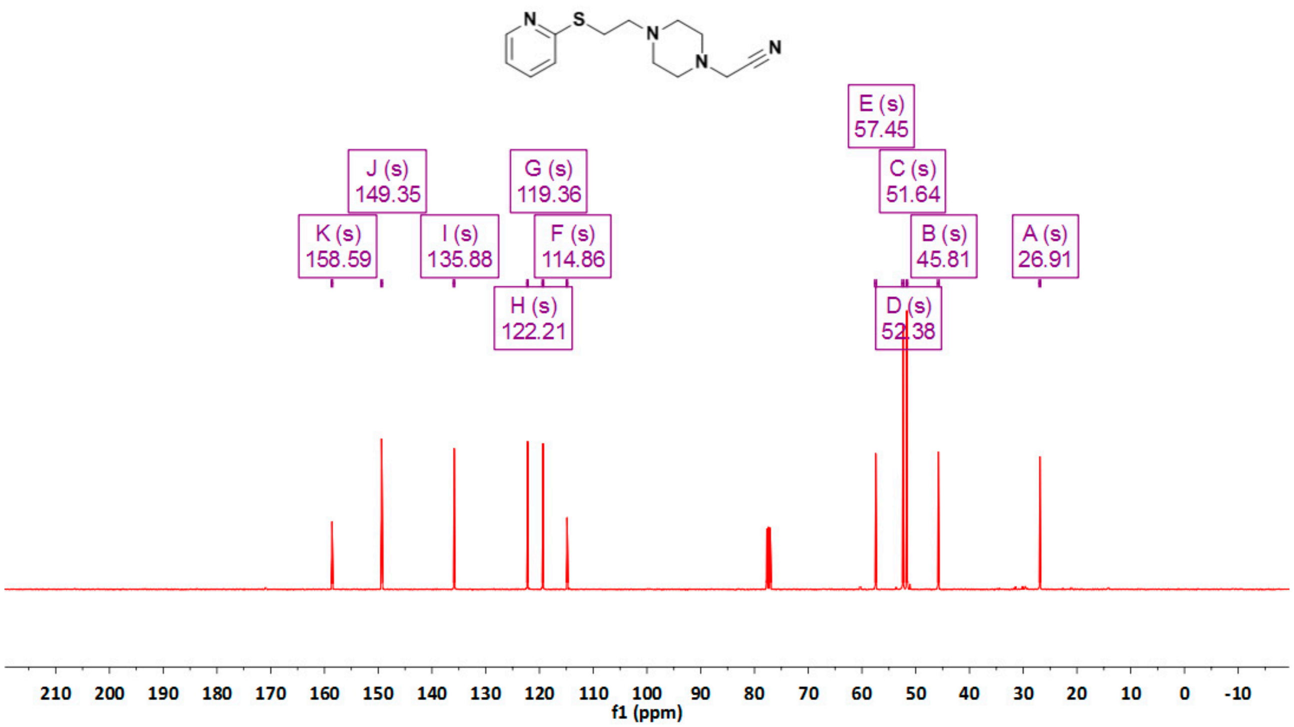

^{1}H NMR spectrum (400 MHz, $CDCl_3$) and ^{13}C NMR spectrum (101 MHz, $CDCl_3$) of 2-(4-(2-(thiophen-2-ylthio)ethyl)piperazinyl)acetonitrile (2x)

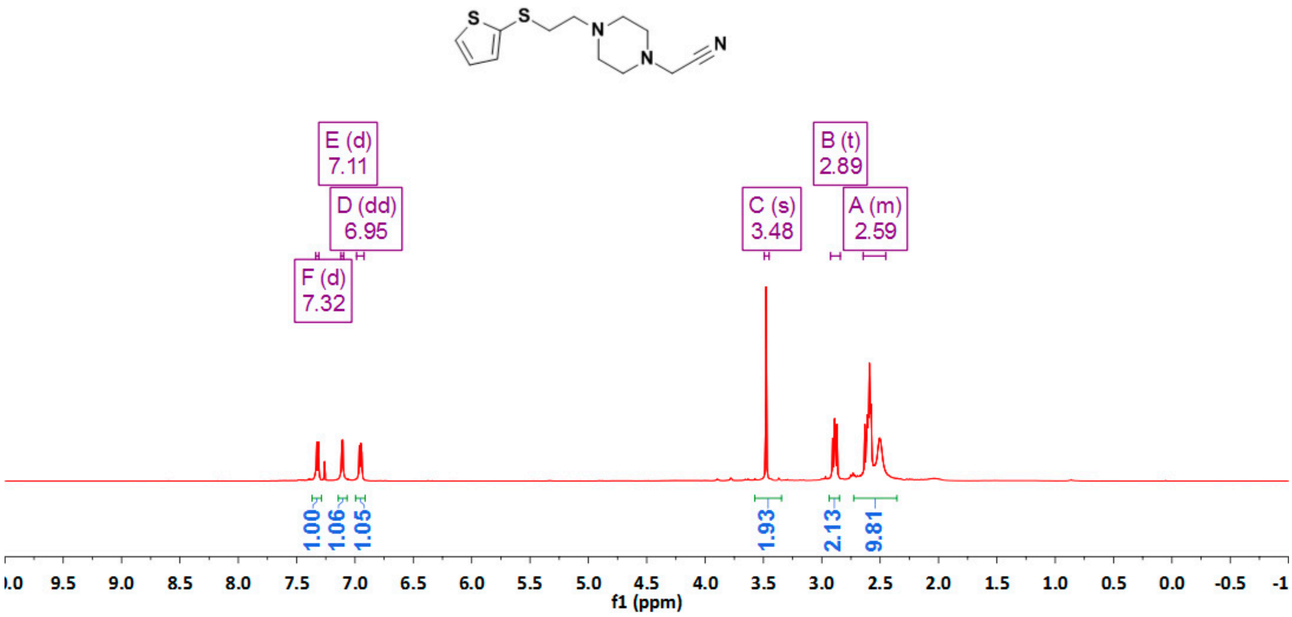

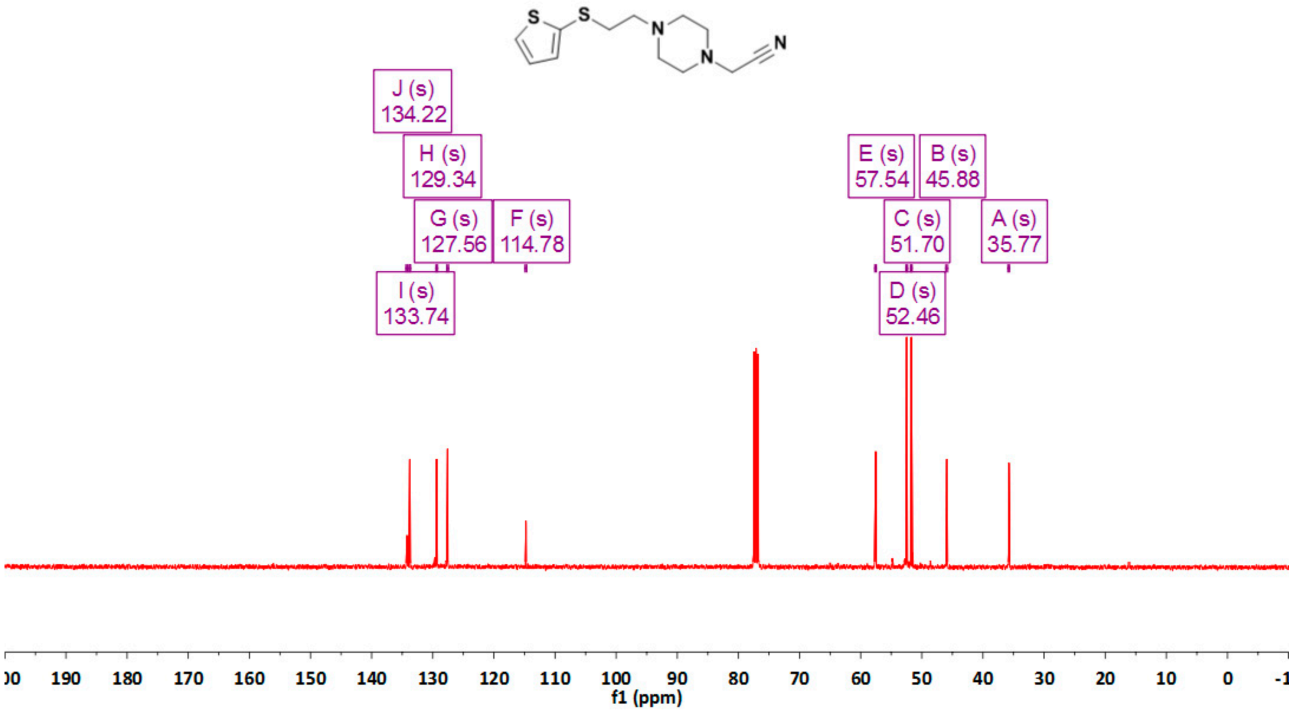

^{1}H NMR spectrum (400 MHz, $CDCl_3$) and ^{13}C NMR spectrum (101 MHz, $CDCl_3$) of 2-(4-(2-((2-methylfuran-3-yl)thio)ethyl)piperazinyl)acetonitrile (2y)

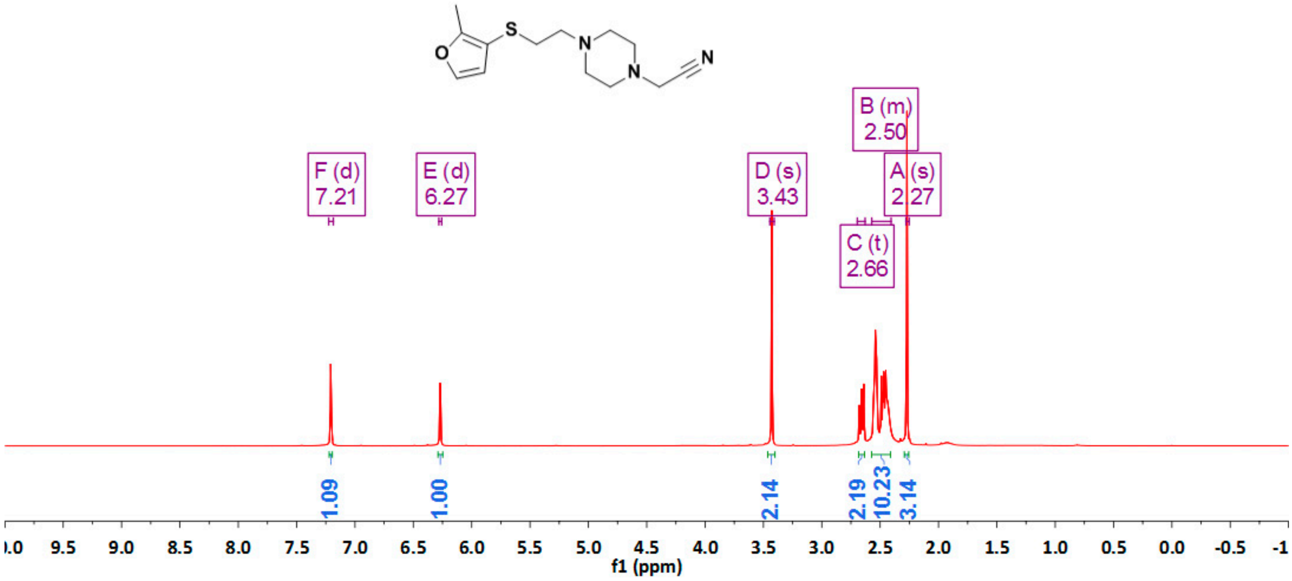

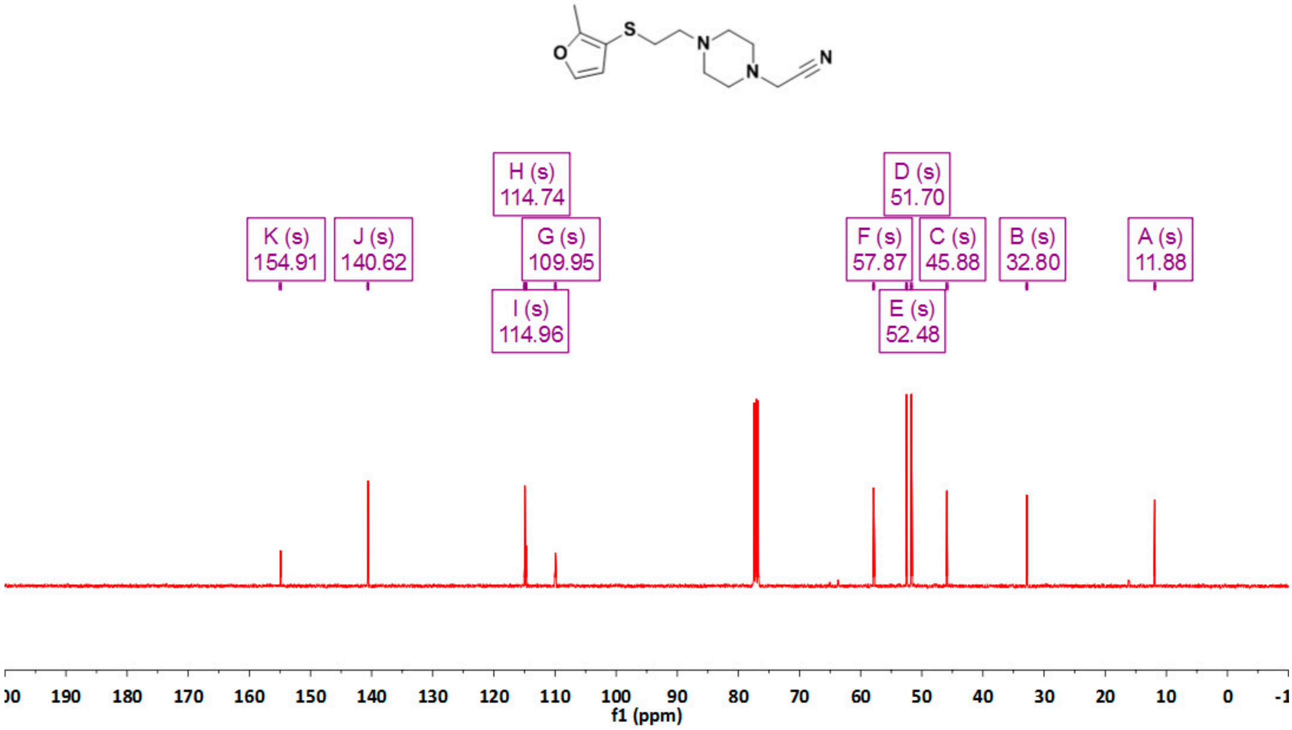

^{1}H NMR spectrum (400 MHz, $CDCl_3$) and ^{13}C NMR spectrum (101 MHz, $CDCl_3$) of 2-(4-(2-(benzo[d]thiazol-2-ylthio)ethyl)piperazinyl)acetonitrile (2z)

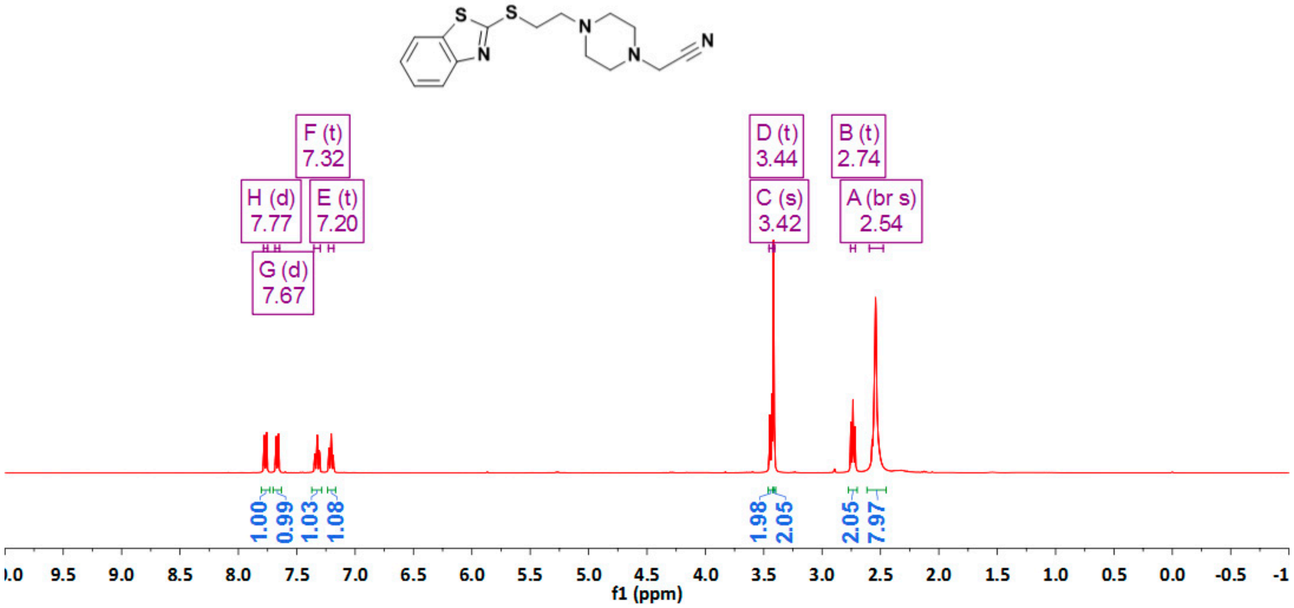

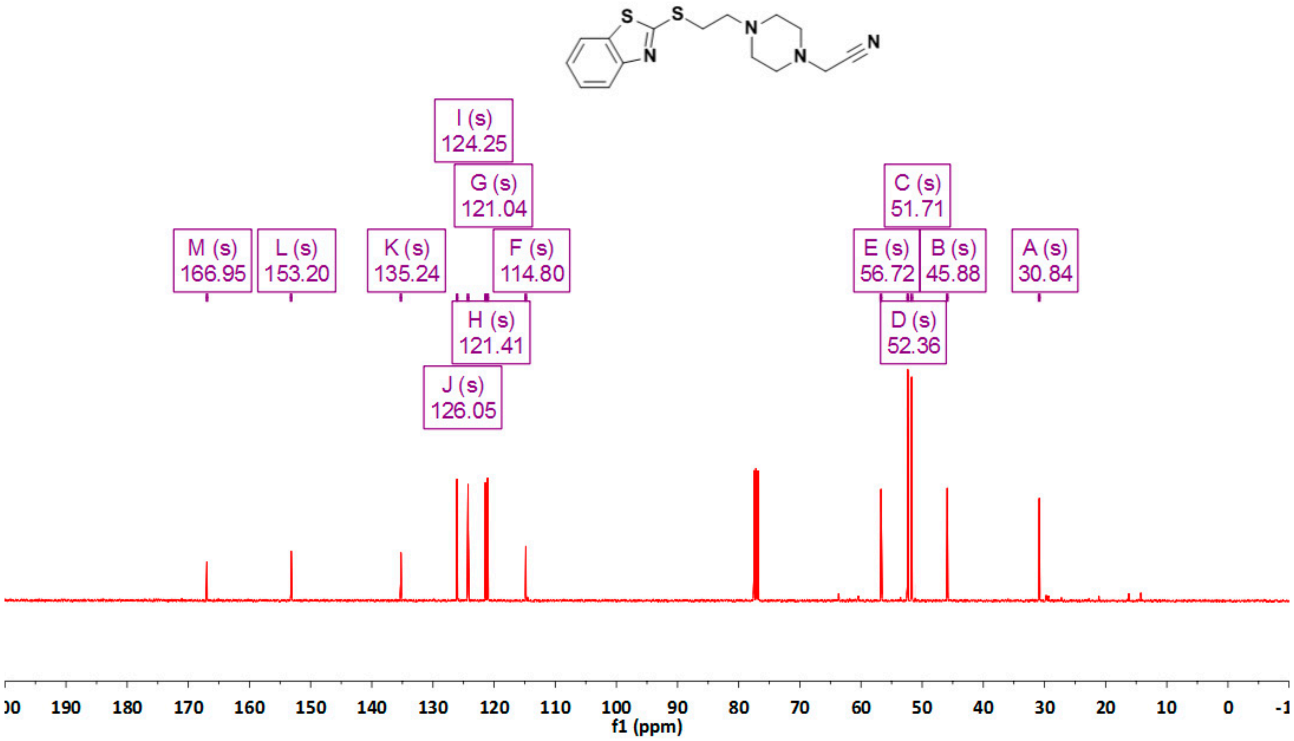

^{1}H NMR spectrum (400 MHz, $CDCl_3$) and ^{13}C NMR spectrum (101 MHz, $CDCl_3$) of 2-(4-(2-(benzo[d]oxazol-2-ylthio)ethyl)piperazinyl)acetonitrile (2aa)

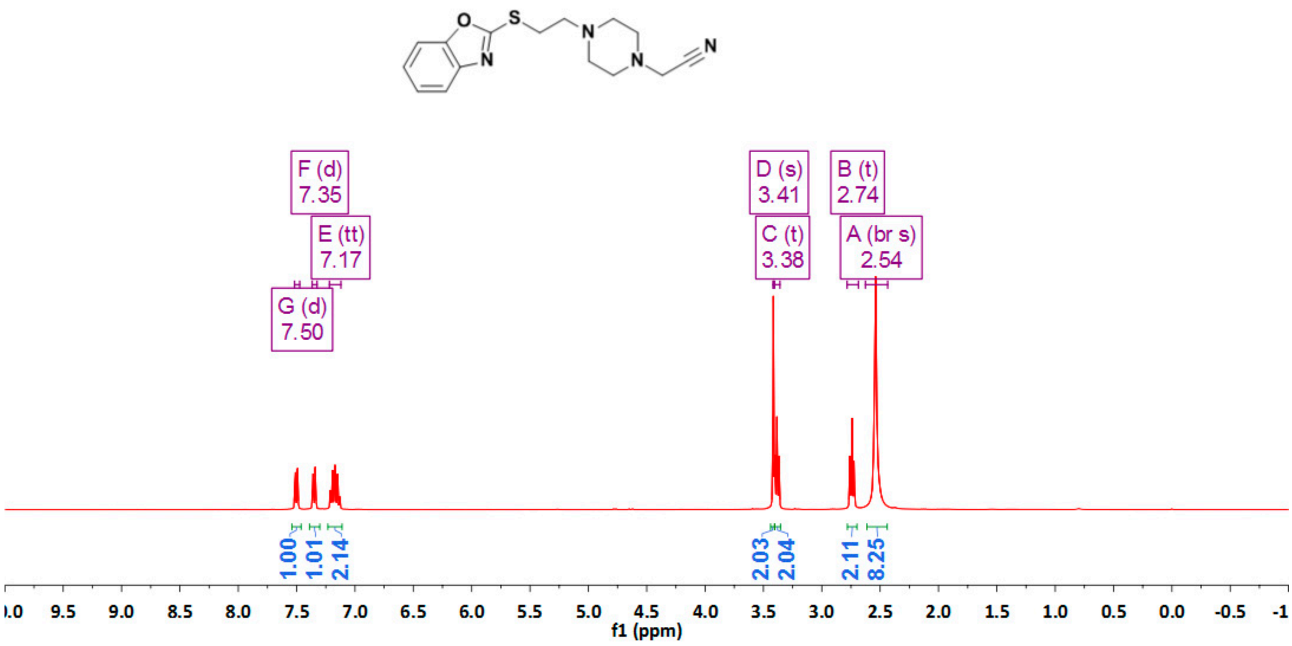

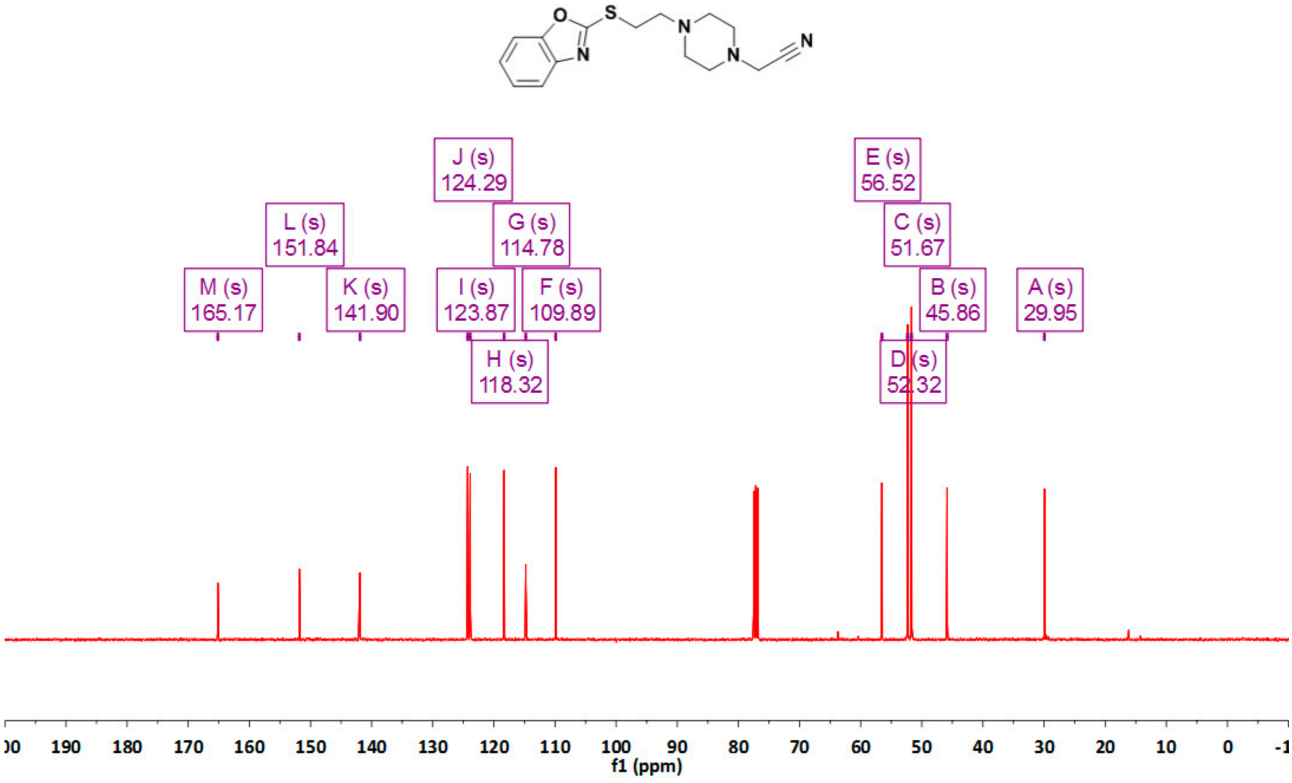

^{1}H NMR spectrum (400 MHz, $CDCl_3$) and ^{13}C NMR spectrum (101 MHz, $CDCl_3$) of 2-(4-(2-((1H-benzo[d]imidazol-2-yl)thio)ethyl)piperazinyl)acetonitrile (2ab)

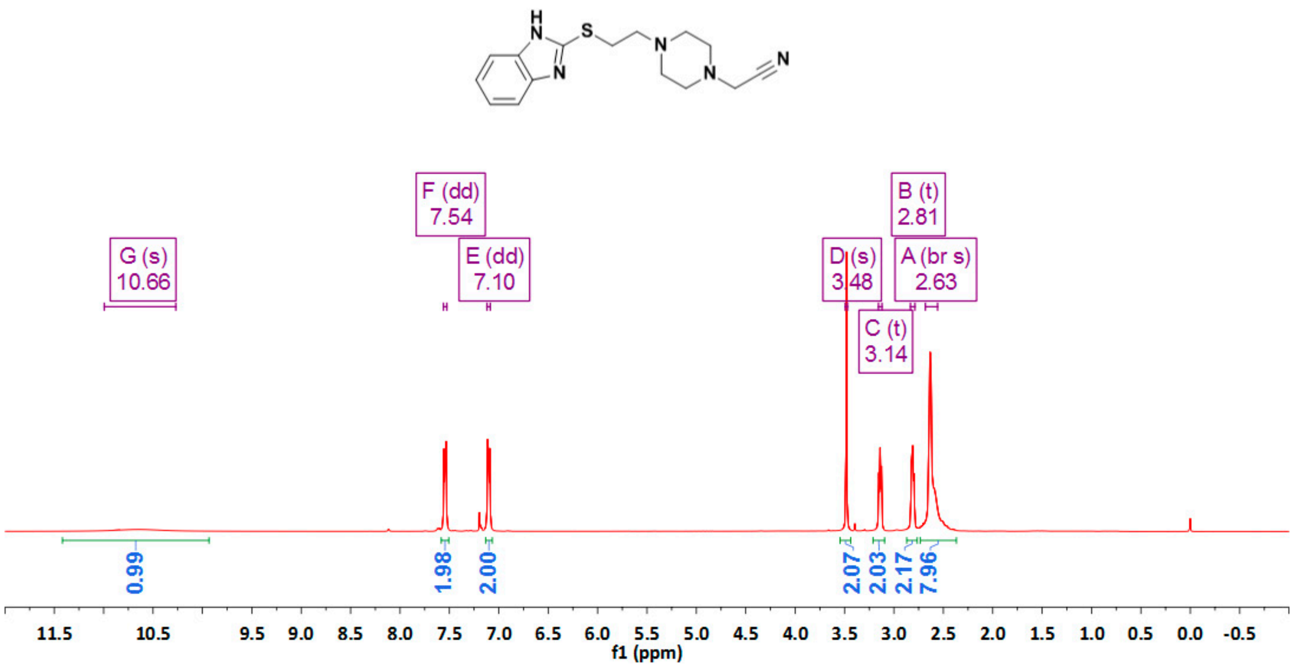

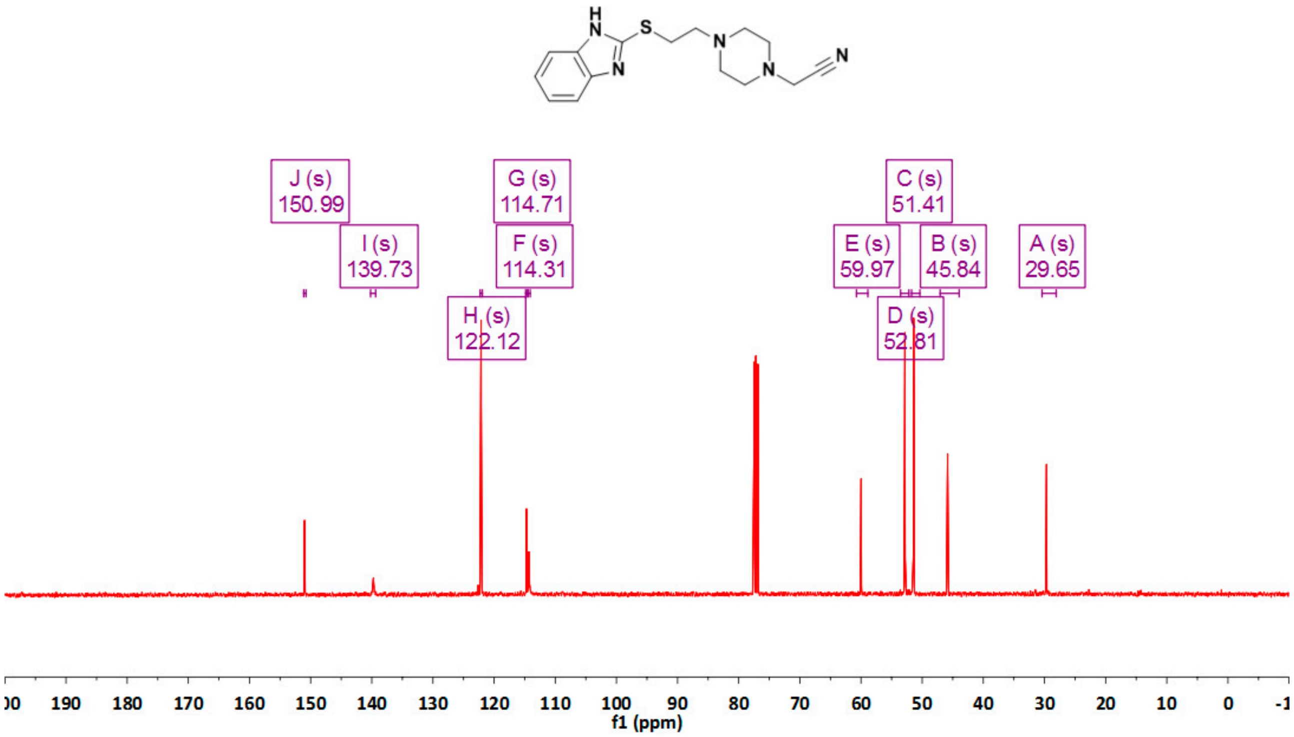

^{1}H NMR spectrum (400 MHz, CDCl$_3$) and ^{13}C NMR spectrum (101 MHz, CDCl$_3$) of 2-(4-(2-(hexylthio)ethyl)piperazinyl)acetonitrile (2ac)

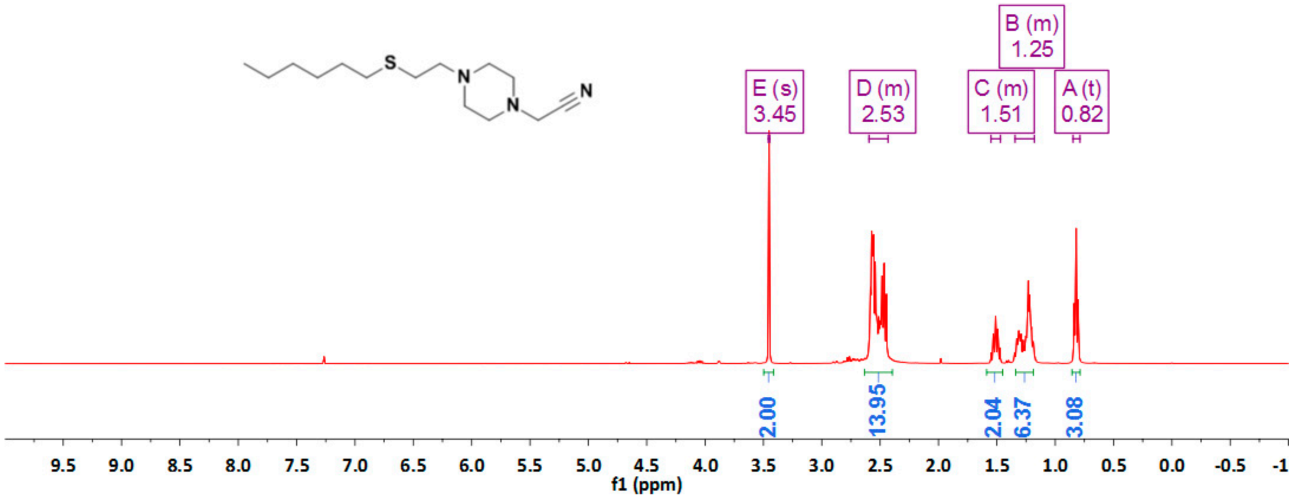

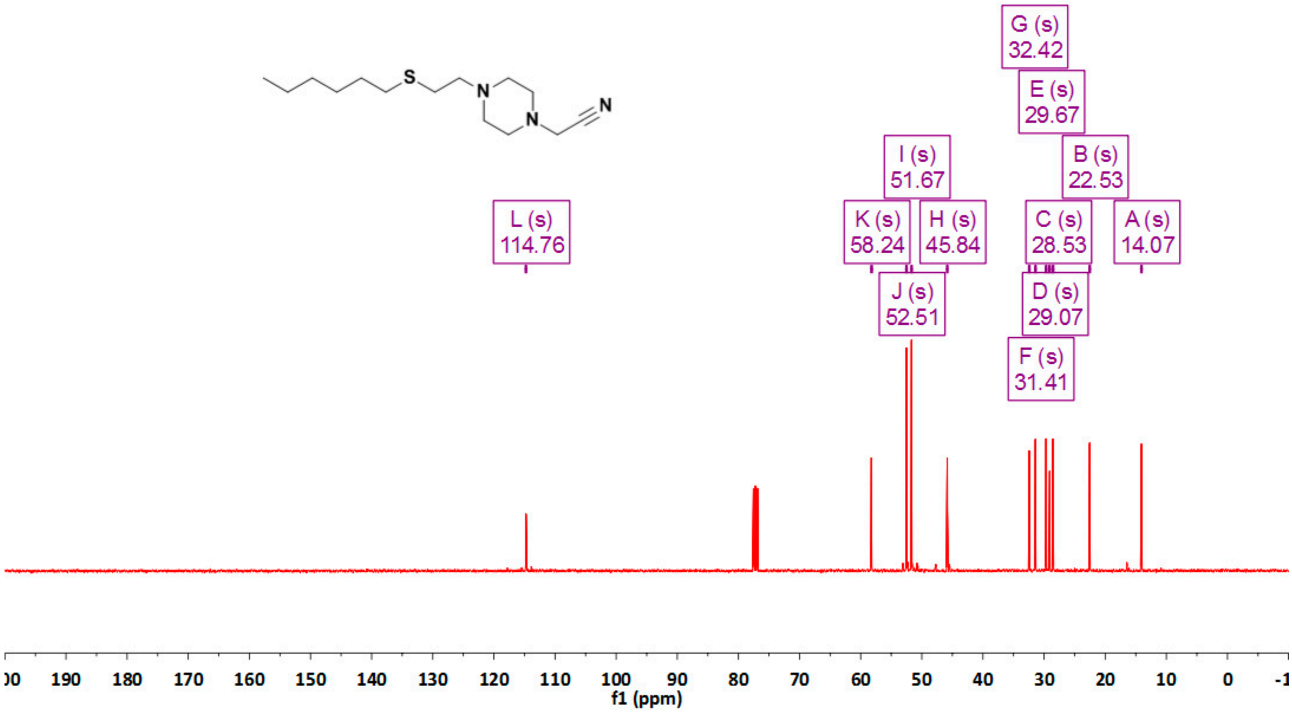

^{1}H NMR spectrum (400 MHz, $CDCl_3$) and ^{13}C NMR spectrum (101 MHz, $CDCl_3$) of 2-(4-(2-(phenylselanyl)ethyl)piperazinl)acetonitrile (2ad)

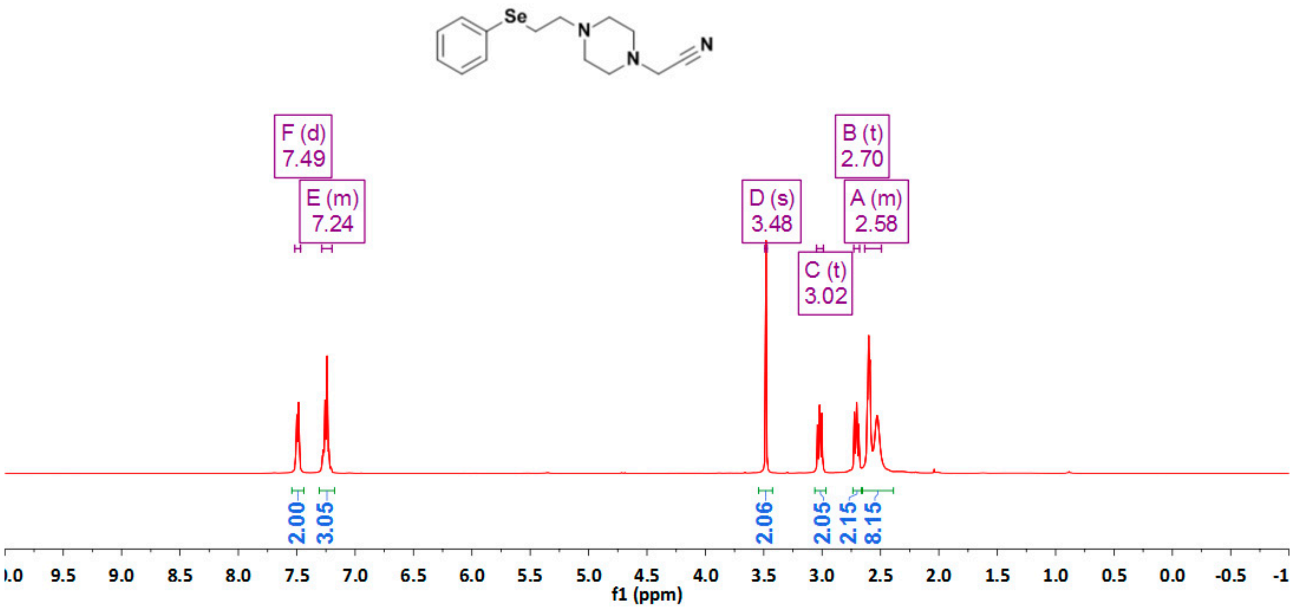

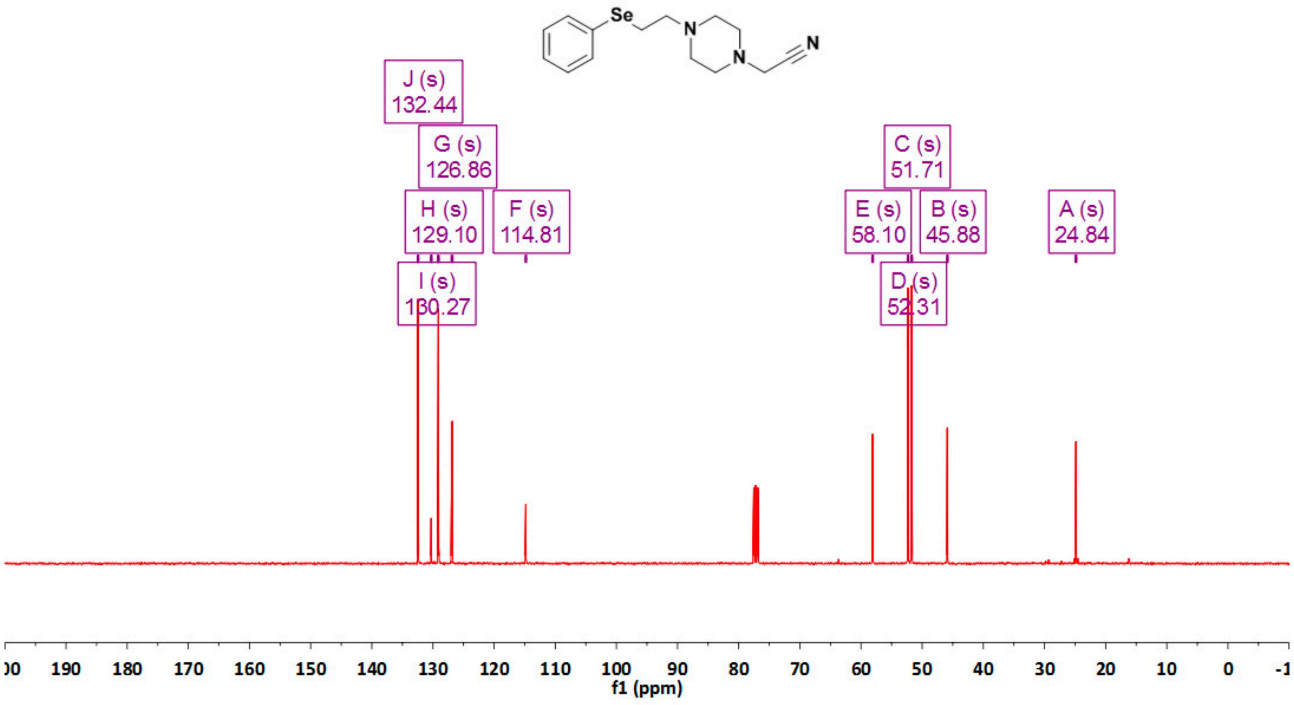

^{1}H NMR spectrum (400 MHz, D_2O) and ^{13}C NMR spectrum (101 MHz, D_2O) of 1-(chloromethyl)-1,4-diazabicyclo[2.2.2]octan-1-ium chloride (CAABC)

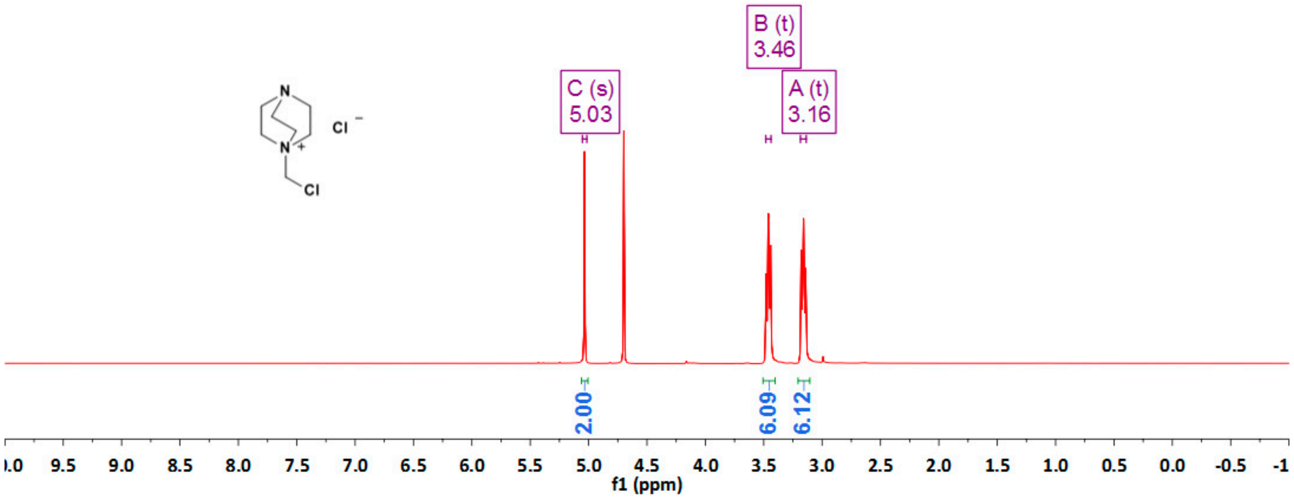

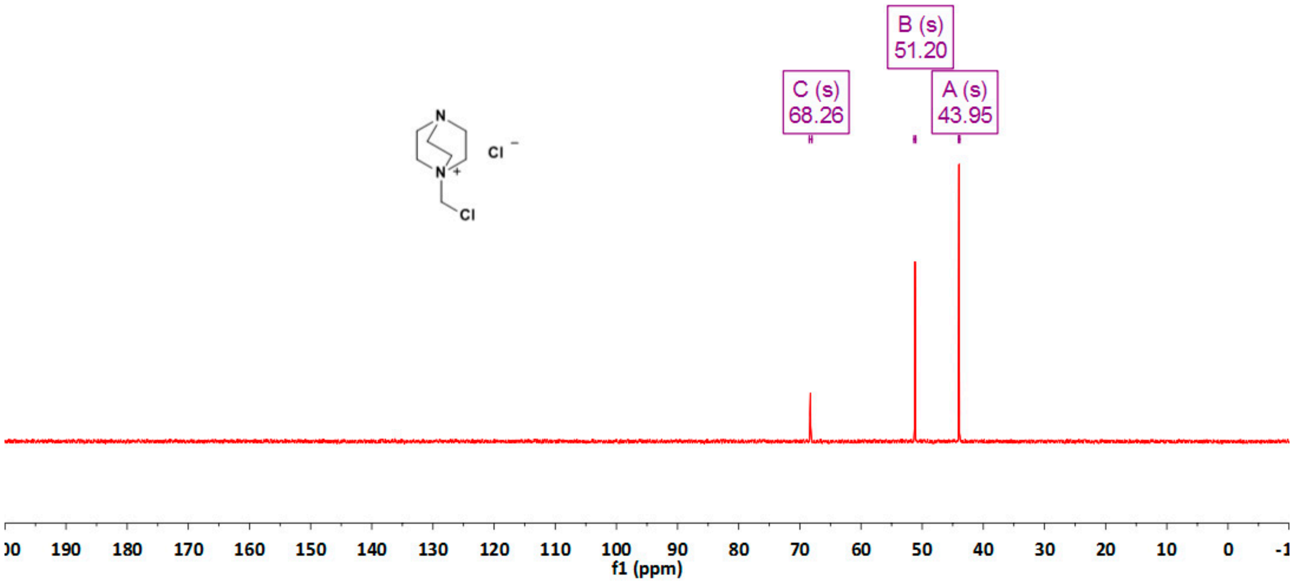

^{1}H NMR spectrum (400 MHz, DMSO-d$_6$) and ^{13}C NMR spectrum (101 MHz, DMSO-d$_6$) of 1-(cyanomethyl)-4-aza-1-azonia bicyclo[2.2.2]octane chloride

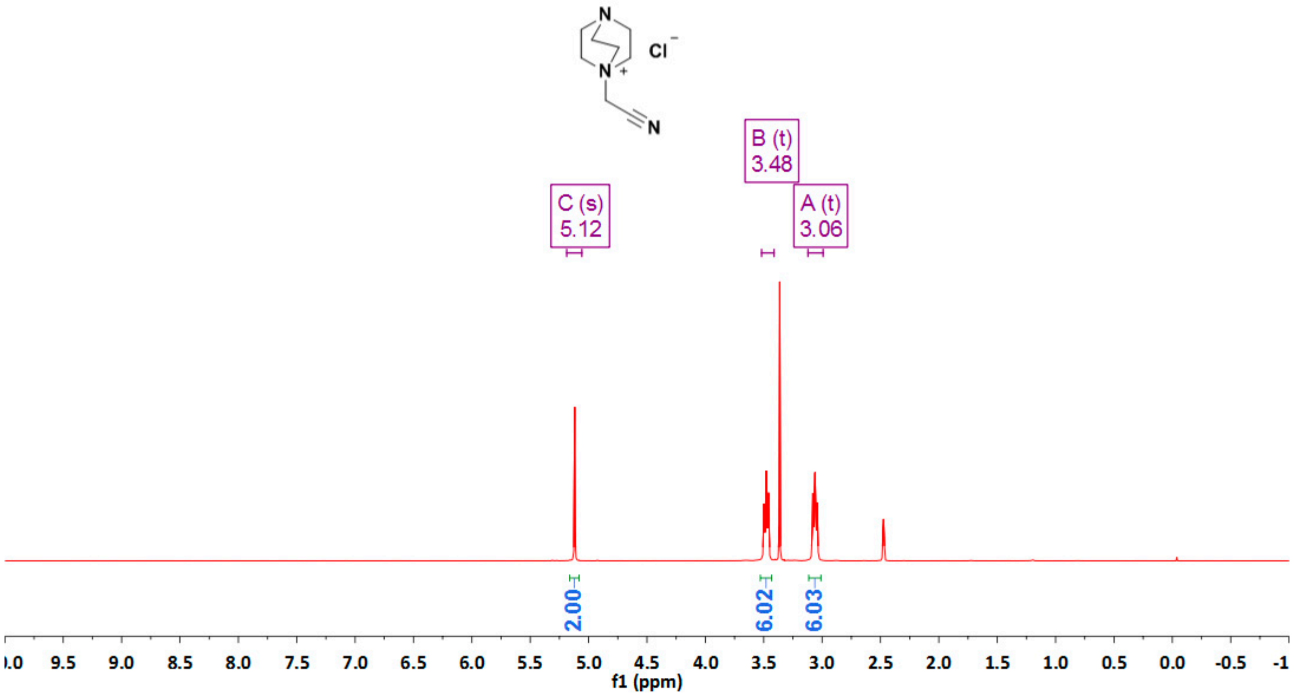

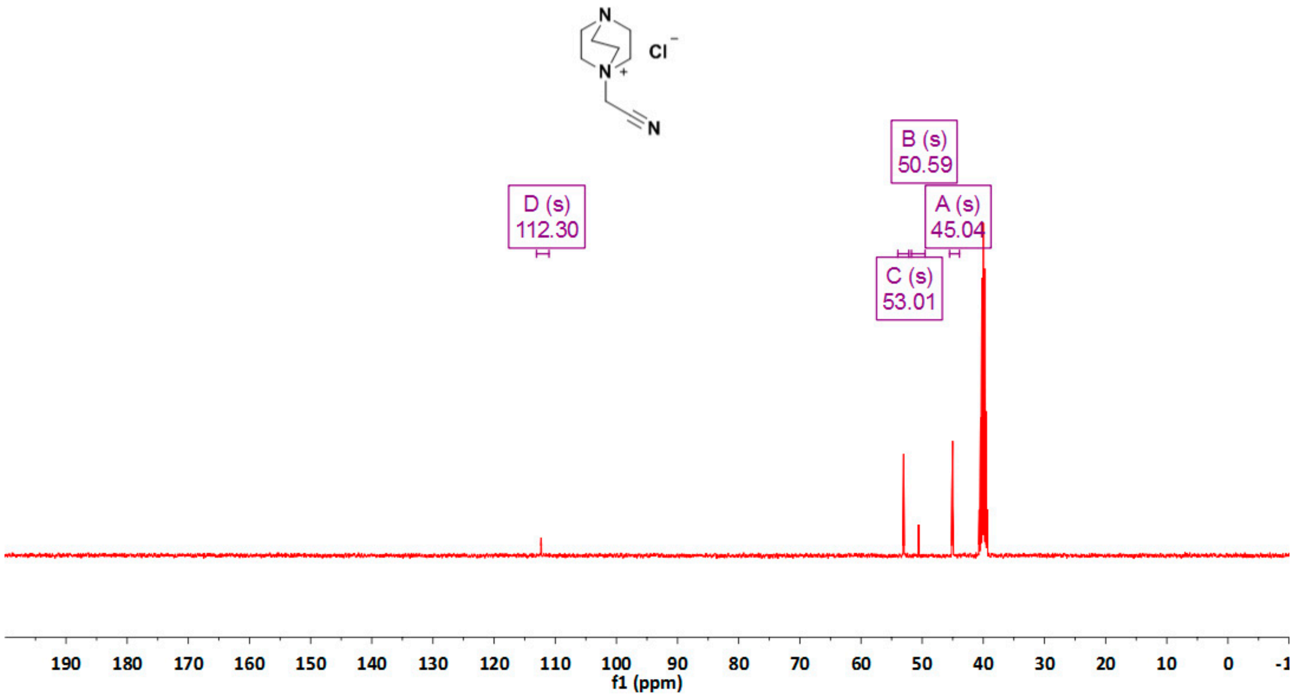

^{1}H NMR spectrum (400 MHz, DMSO-d_6) and ^{13}C NMR spectrum (101 MHz, DMSO-d_6) of EAABC

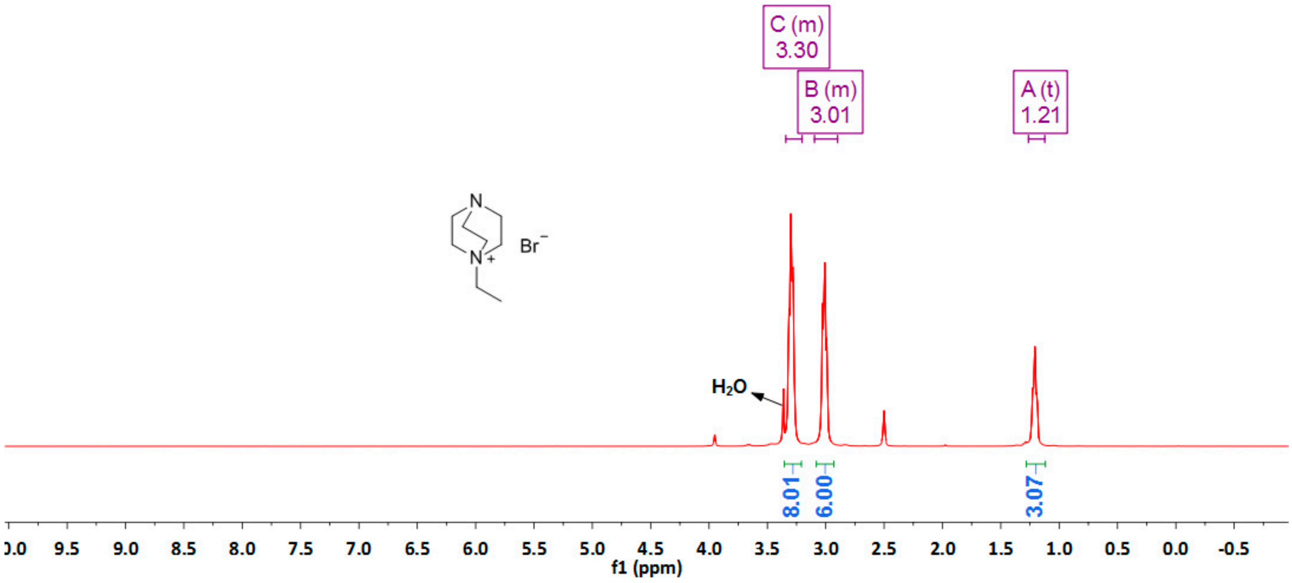

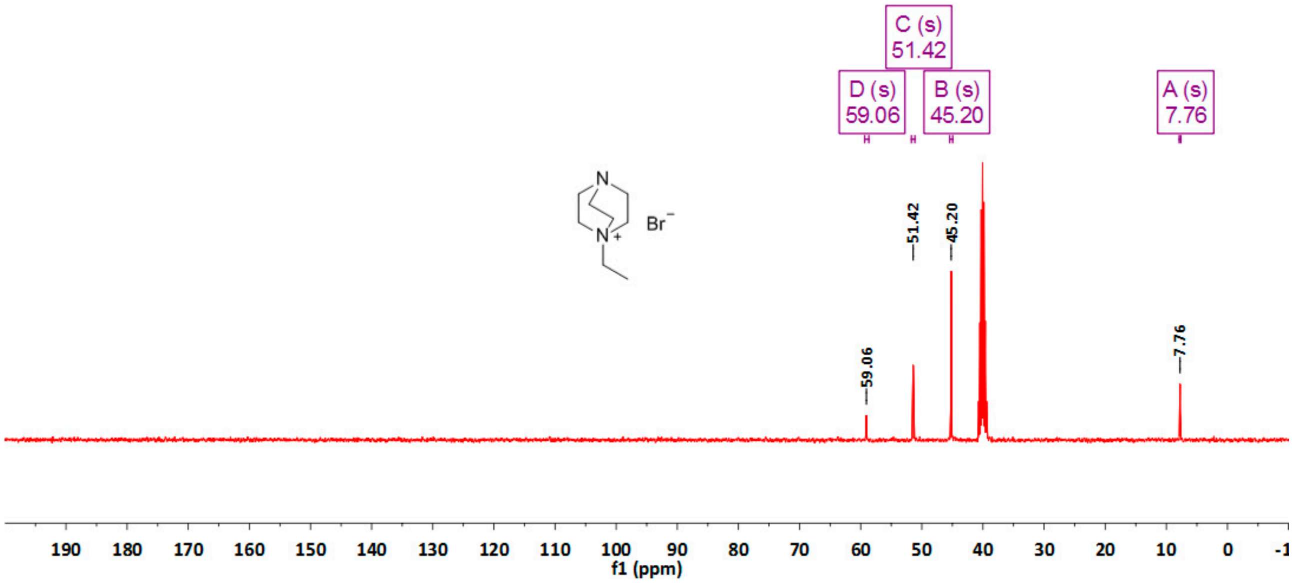

^{1}H NMR spectrum (400 MHz, $CDCl_3$) and ^{13}C NMR spectrum (101 MHz, $CDCl_3$) of compound 3a

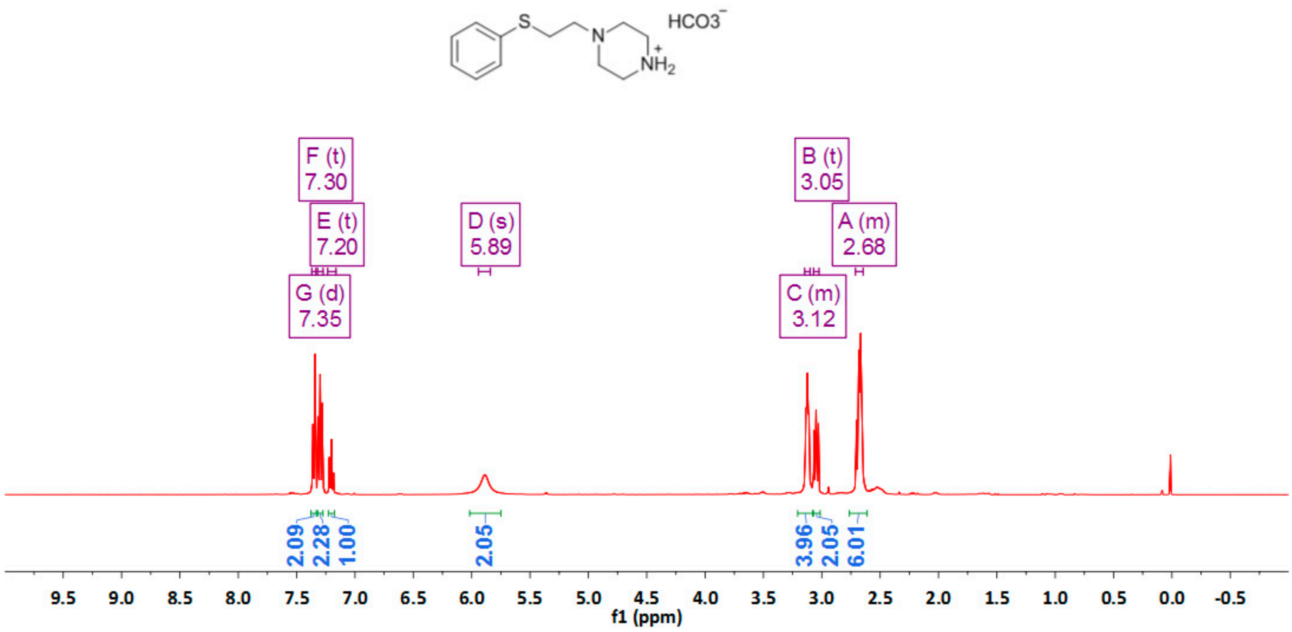

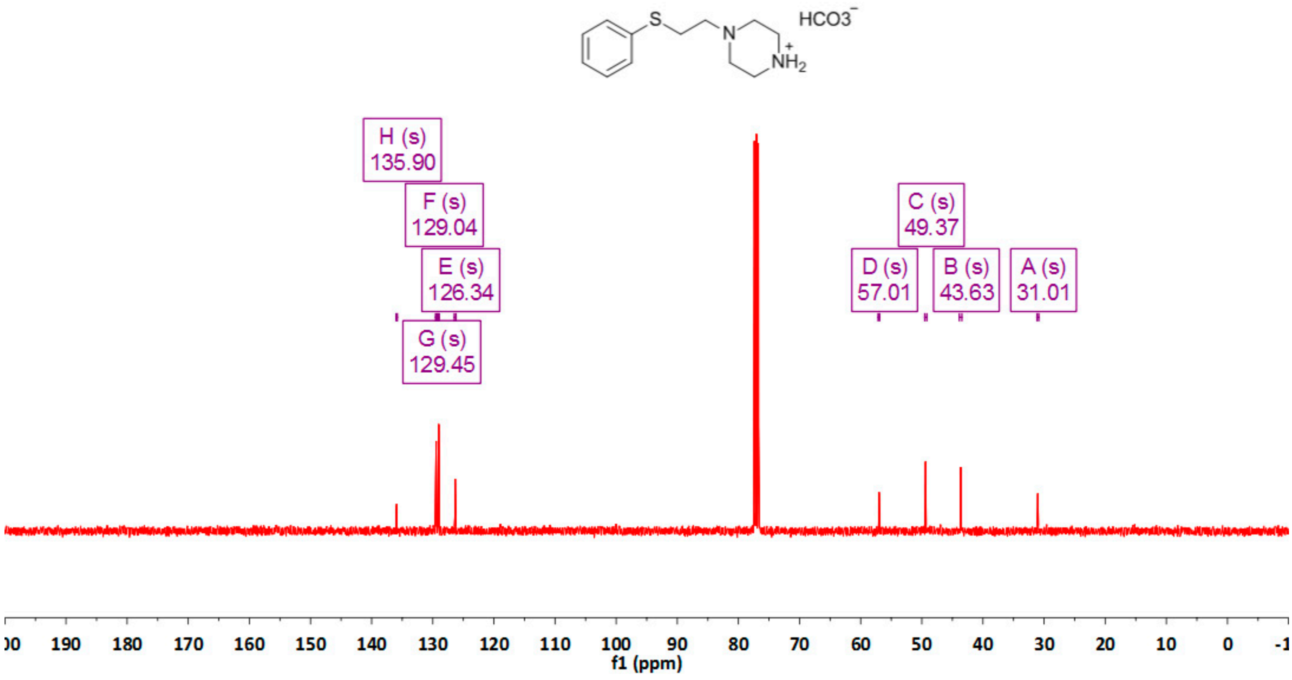

^{1}H NMR spectrum (400 MHz, $CDCl_3$) and ^{13}C NMR spectrum (101 MHz, $CDCl_3$) of compound 4a

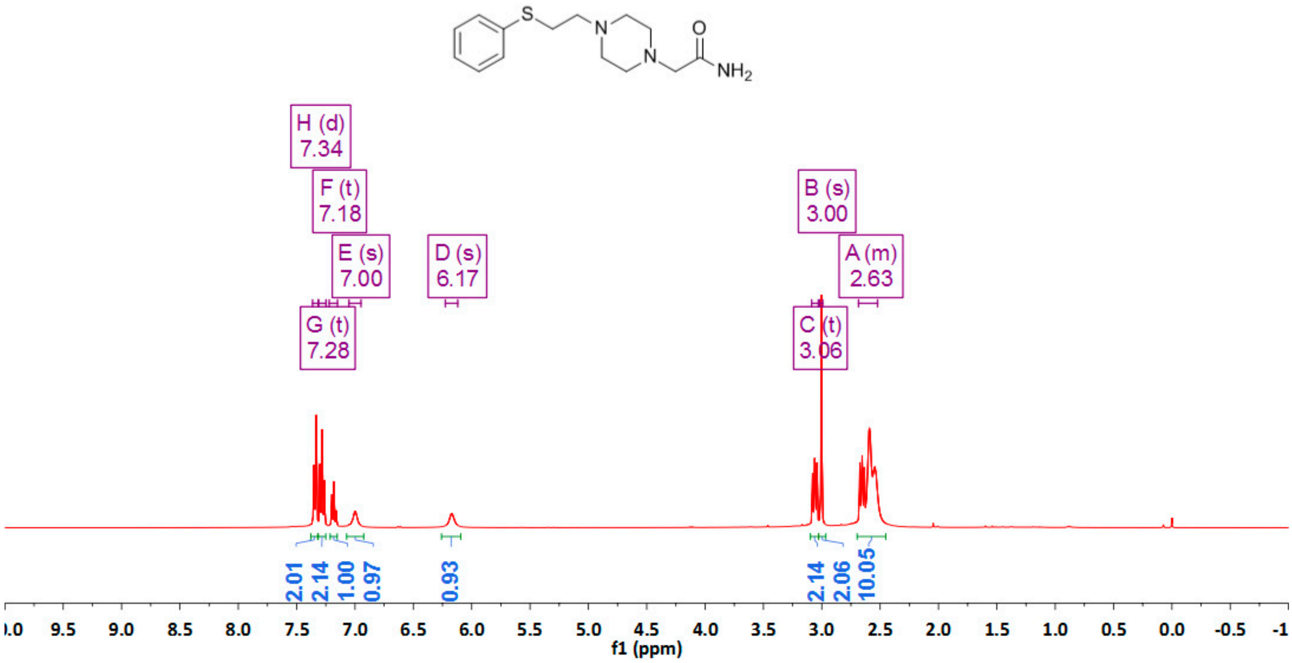

^{1}H NMR spectrum (400 MHz, $CD_3OD/CDCl_3$ (1:1)) of 2-(4-(2-((1H-benzo[*d*]imidazol-2-yl)thio)ethyl)piperazin-1-yl)acetamide (4b)

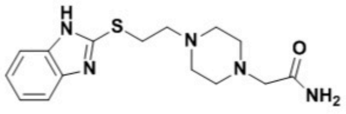

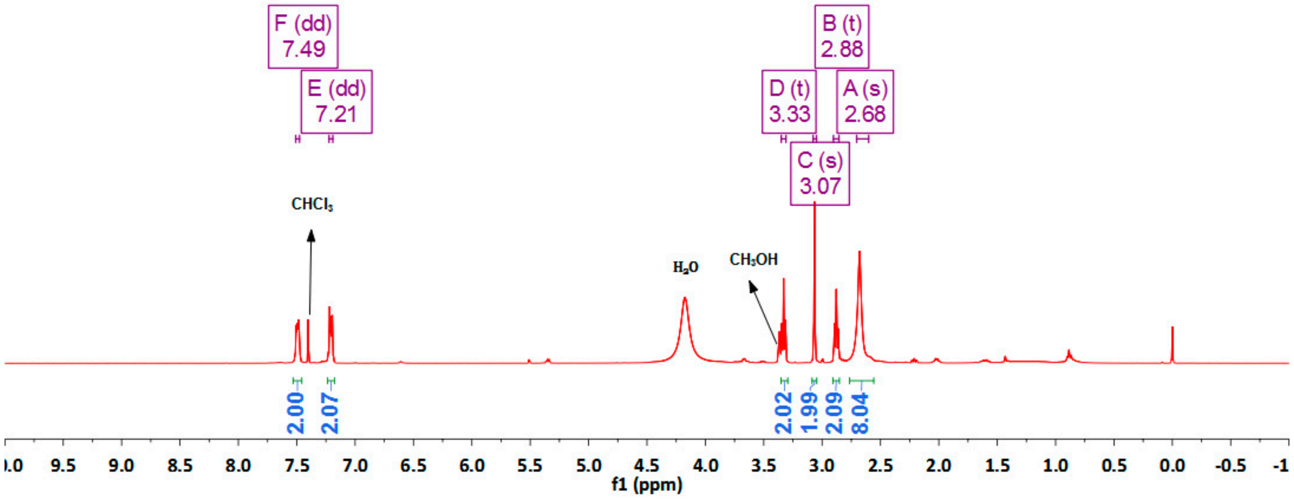

^{1}H NMR spectrum (400 MHz, $CD_3OD/CDCl_3$ (1:1)) and ^{13}C NMR spectrum (101 MHz, $CD_3OD/CDCl_3$ (1:1)) of 1-(2-(phenylthio)ethyl)piperazine (3b)

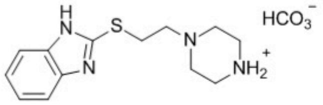

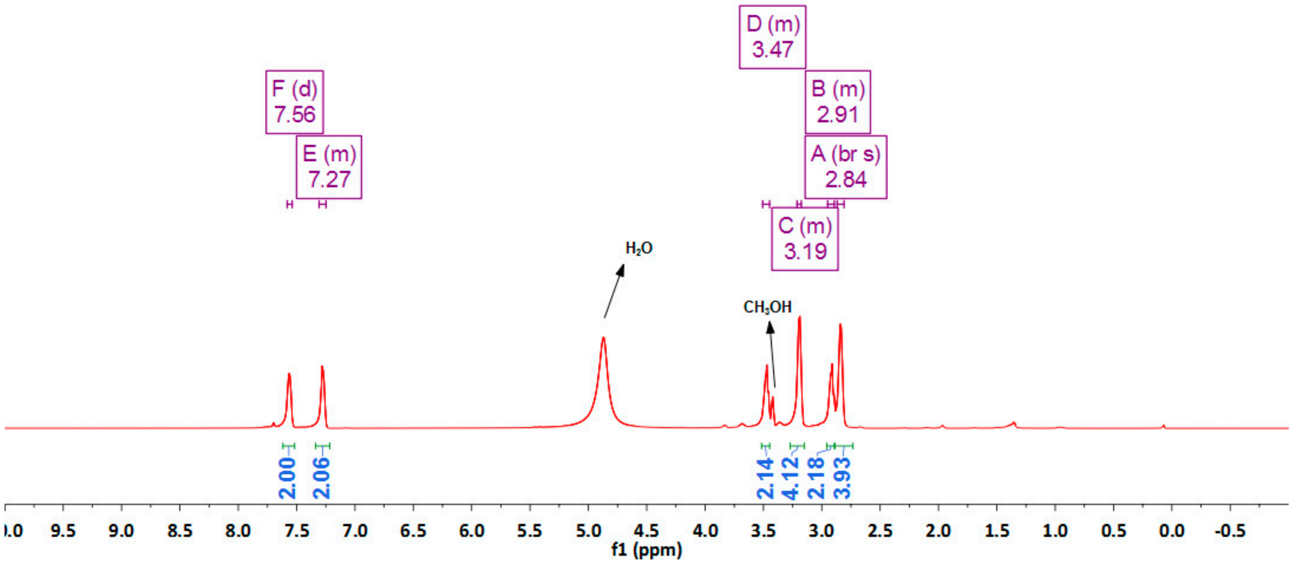

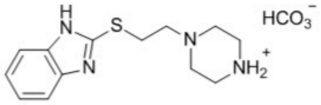

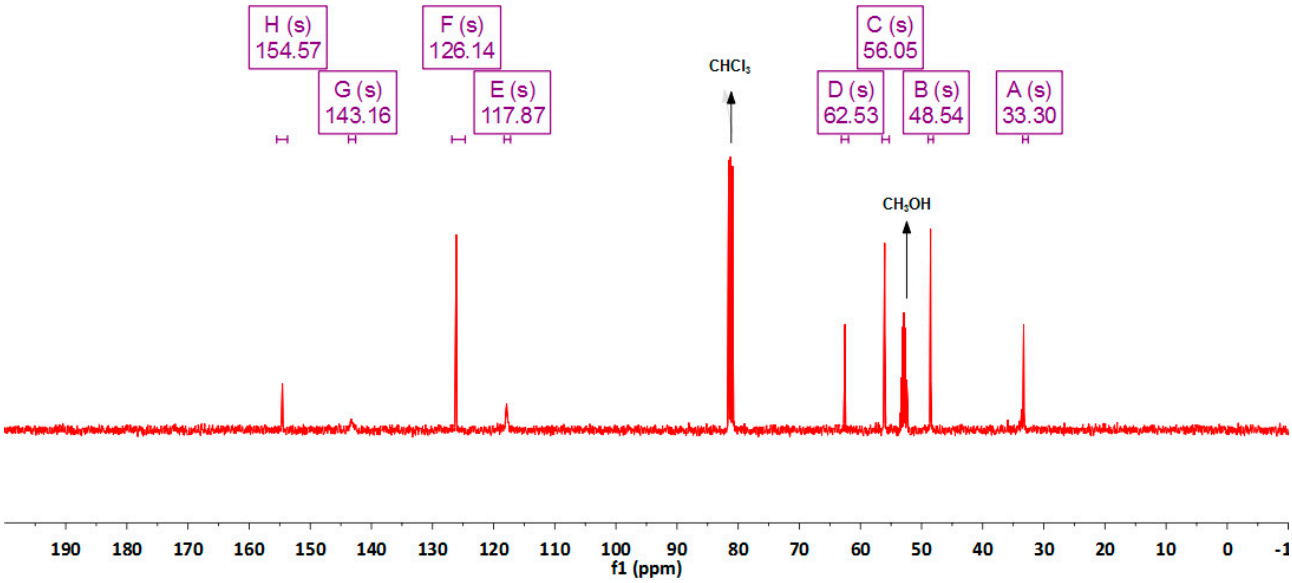

^{1}H NMR spectrum (400 MHz, DMSO-d_6) and ^{13}C NMR spectrum (101 MHz, DMSO-d_6) of 2-[4-[2-(Benzimidazol-2-ylthio)ethyl]piperazin-1-yl]-*N*-(2,6-diisopropylphenyl)acetamide (5a)

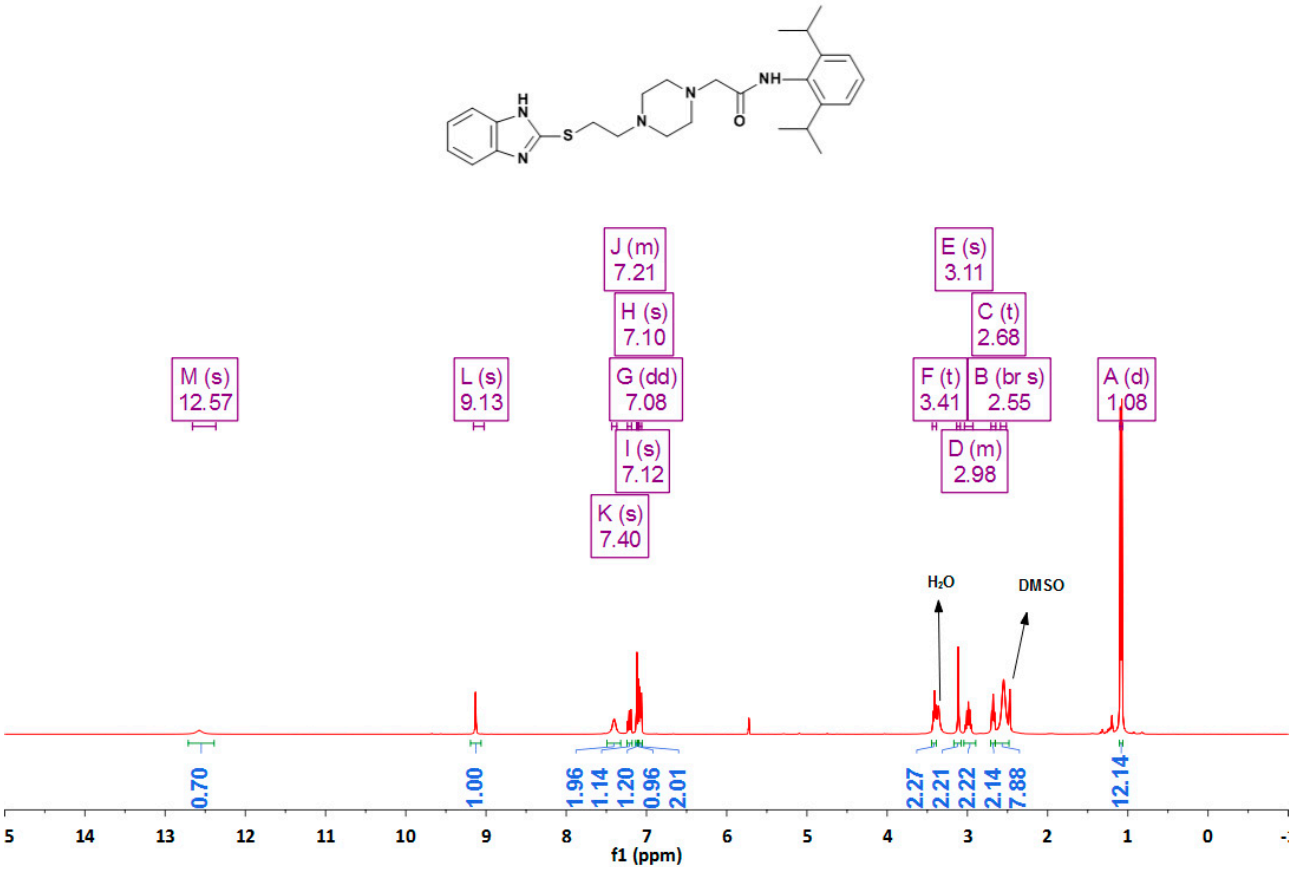

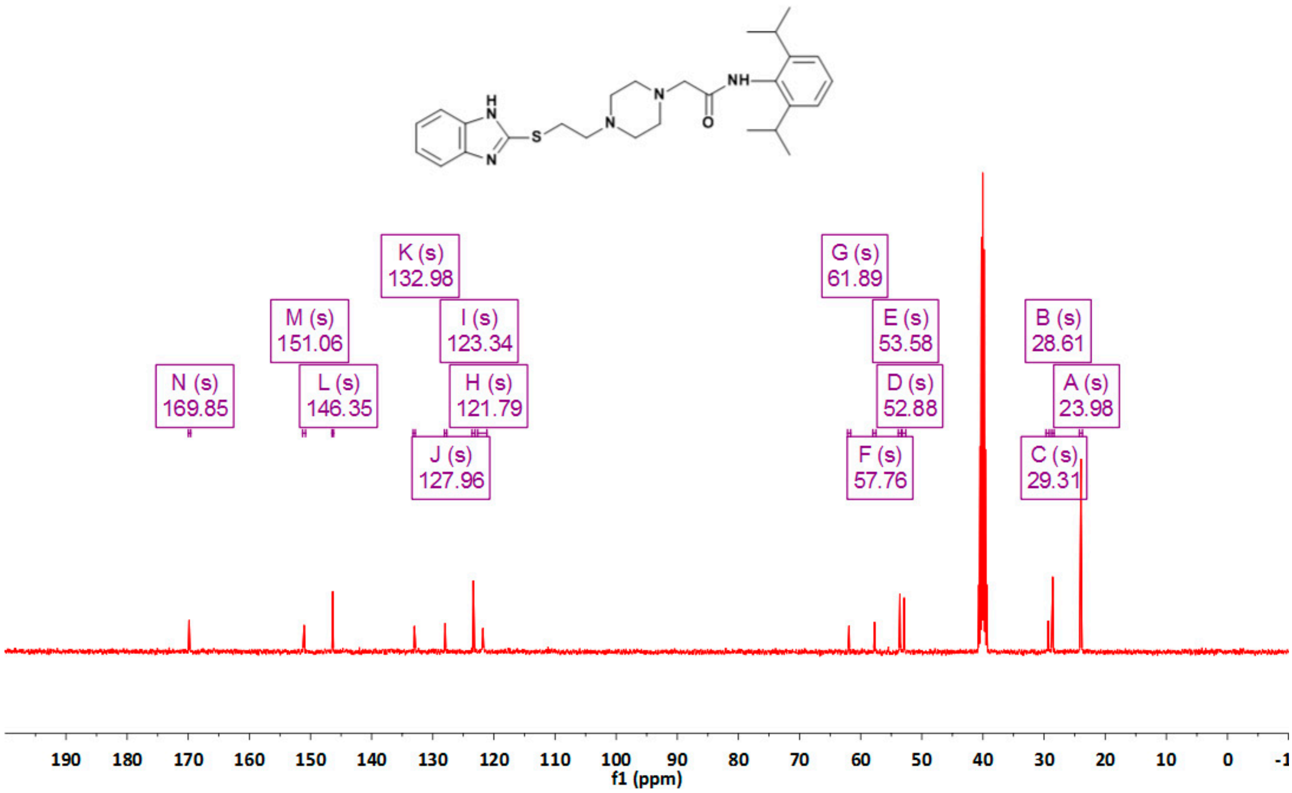

^{1}H NMR spectrum (400 MHz, $CDCl_3$) and ^{13}C NMR spectrum (101 MHz, $CDCl_3$) of 5b

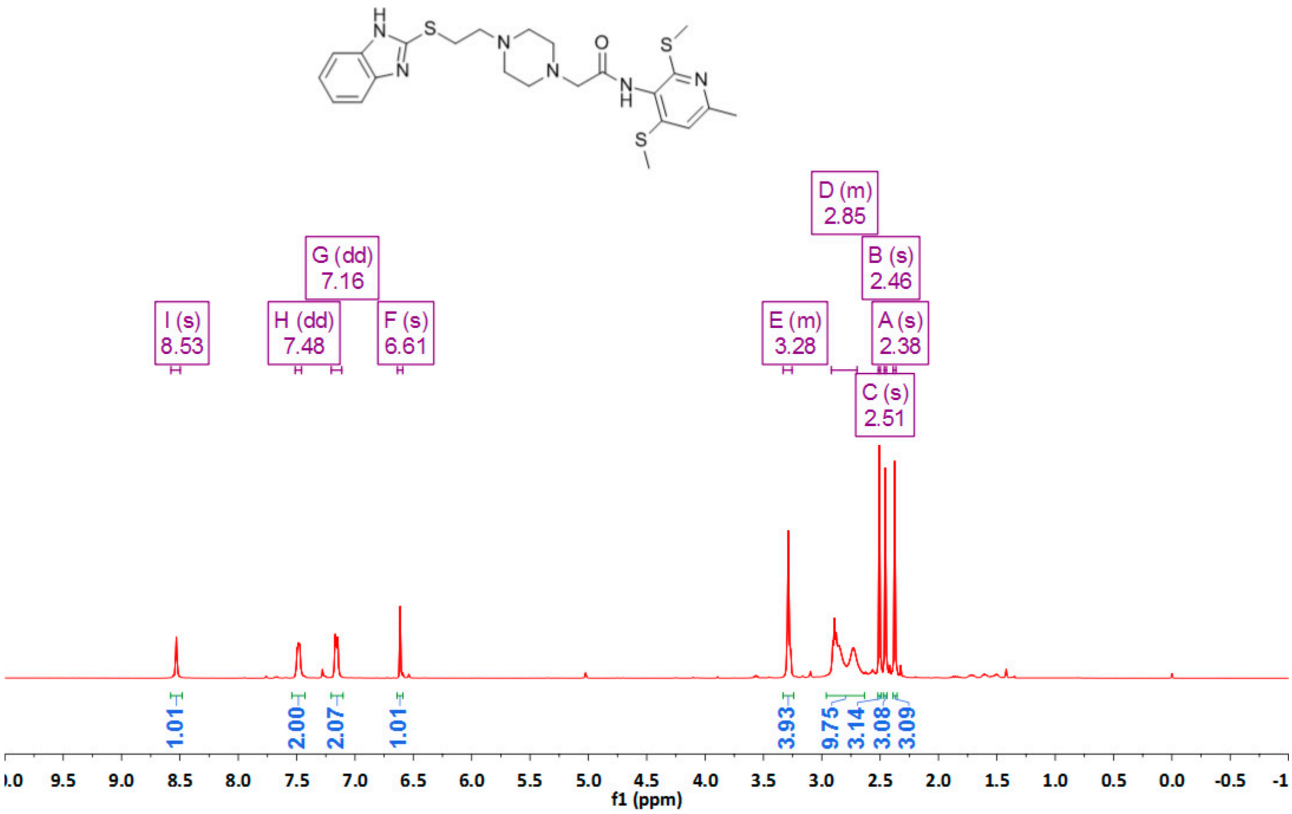

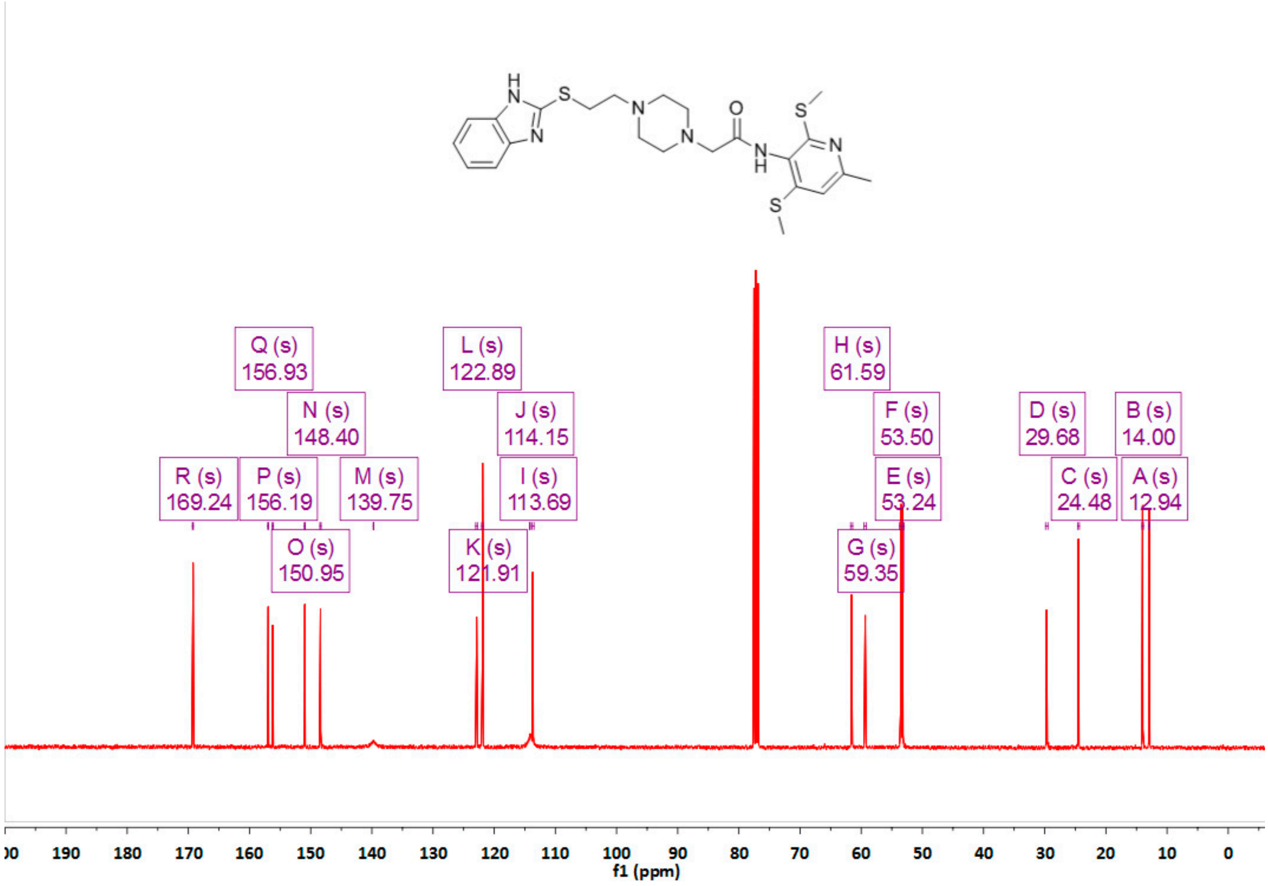

Supplement: Supplementary file 1 [file molecules-29-03723-s001.zip › Supporting Information.pdf]
